# Efficient human-machine control with asymmetric marginal reliability input devices

**DOI:** 10.1371/journal.pone.0233603

**Published:** 2020-06-01

**Authors:** John H. Williamson, Melissa Quek, Iulia Popescu, Andrew Ramsay, Roderick Murray-Smith

**Affiliations:** School of Computing Science, University of Glasgow, Glasgow, Scotland, United Kingdom; Hong Kong University of Science and Technology, HONG KONG

## Abstract

Input devices such as motor-imagery brain-computer interfaces (BCIs) are often unreliable. In theory, channel coding can be used in the human-machine loop to robustly encapsulate intention through noisy input devices but standard feedforward error correction codes cannot be practically applied. We present a practical and general probabilistic user interface for binary input devices with very high noise levels. Our approach allows any level of robustness to be achieved, regardless of noise level, where reliable feedback such as a visual display is available. In particular, we show efficient zooming interfaces based on feedback channel codes for two-class binary problems with noise levels characteristic of modalities such as motor-imagery based BCI, with accuracy <75%. We outline general principles based on separating channel, line and source coding in human-machine loop design. We develop a novel selection mechanism which can achieve arbitrarily reliable selection with a noisy two-state button. We show automatic online adaptation to changing channel statistics, and operation without precise calibration of error rates. A range of visualisations are used to construct user interfaces which implicitly code for these channels in a way that it is transparent to users. We validate our approach with a set of Monte Carlo simulations, and empirical results from a human-in-the-loop experiment showing the approach operates effectively at 50-70% of the theoretical optimum across a range of channel conditions.

## 1 Introduction

Most mainstream devices used for human input are reliable; for example, keyboard typing has a typical error rate of around 6-7% [1]. This has led to interaction models which apply occasional corrective steps, such as backspace, to resolve infrequent errors. However, there are **marginal reliability** input devices, particularly in assistive technology, where errors are sufficiently frequent that this approach fails catastrophically. The classic example is a BCI where error rates in even binary selection exceed 30% for some subjects [2]. The result is interfaces that are susceptible to unrecoverable correction cascades where attempts to rollback previous errors induce even more errors.

In this paper we consider the problem of implementing efficient and transparent **channel coding** in human-machine control, encoding user intention robustly so it can be transferred without error over unreliable channels and without introducing a cognitive or perceptual burden. We apply our ideas to induce robustness to a broad range of transient error sources in human-machine interaction, including mental “slips”, noise in the human motor system, and context-induced disturbances like electrical noise or vibration. Our approach puts designing for error at the heart of the problem, rather than as a corrective step applied after the fact. It makes interactive control with binary classifier accuracies <75% viable and allows for graceful degradation of performance that does not exhibit cliff-edge drops in communication as input reliability drops. We use **reliability** to refer to long term **accuracy**, where **accuracy** is 1-**error rate**. An interface with binary inputs which is “90% reliable” or uses a classifier with “90% accuracy” will result in an input error 10% of the time. We develop a theoretical model for designing for unreliable channels which draws on information theory to map the fundamental steps of entropy coding, channel coding and line coding onto elements of human-machine control. This melds information theory with human factors and interface design. Using this framework we show how closed-loop control in systems which have asymmetry between input and feedback channels can be used to implement capacity-approaching channel codes without the user even being aware of the process. Inspired by our frustration at making electroencephalogram (EEG)-based brain-computer interfaces (BCIs) usable with standard interactions, we specifically focus on the channel coding problem for very noisy binary inputs. We show that posterior matching codes are highly effective and can be adapted to develop control schemes that embed these algorithms in spatial selection tasks.

### 1.1 Contributions

- A theoretical framework to approach design for marginal reliability input devices.
- An adaptation of Horstein’s algorithm [3], to zooming user interfaces for asymmetric interfaces where input is corrupted but high-bandwidth noise-free feedback is available.
- A simple automatic online adaptation algorithm that can cope with varying channel statistics for both biased and unbiased channels.
- Monte Carlo simulation showing the behaviour of this decoder under realistic configurations including channel bias, non-stationarity and mismatched statistics.
- Experimental results with human participants showing that the interface can fuse together binary inputs optimally across a range of reliability and channel bias levels.

### 1.2 Assistive technology channels

As an illustration, many text input systems use backspace as a correction system. Mis-typing a key is relatively common (e.g. around 6% keystrokes are mistakes [1]), but each keystroke communicates a substantial amount of information. Typing is predicated on a model where typists never repeatedly miss backspace and cause a **correction cascade** [4]; an user in danger of doing so will slow down to achieve a tolerable balance between correction and entry. However, there are many interfaces where being more careful is not possible and backspace is provably unusable as a correction modality [5]. This requires a different approach, where the unreliability is not patched up at the end but acknowledged in the design from the start. These high-error channels often occur in assistive technologies, where users’ motor skills are impaired such that they cannot operate standard input devices efficiently [6]. This might arise through underlying motor disorders, or through situational impairment (such as high vibration environments or cumbersome protective clothing). This includes input devices like EMG, single muscle switches, breath sensors or eye-trackers. For example, situationally-induced interaction errors can be observed in pedestrians walking and operating pressure sensors on mobile devices [7] or engaging in touchscreen pointing while carrying objects [8], where pointing at standard button size targets can result in error rates exceeding 30%. Even standard keyboard and mouse interactions can have very high error rates for motor impaired subjects [6]. Even with appropriate sensors, standard input paradigms such as spatial targeting or transient timing can be disrupted by tremor, fatigue or spasticity. The user groups with the most extreme needs are those who have no effective residual motor function; “locked-in patients” [9]. These users rely on a direct neural interface which bypasses the motor system entirely [10–12]. Unfortunately, among those systems which are sufficiently non-invasive to be practical for widespread use, communication rates are low and noise levels are very high. Our work is primarily concerned with making input practical with channels with properties akin to two class motor imagery EEG—effectively a slow, heavily corrupted, non-stationary and biased two state button. The principles generalise to other input devices such as single switch inputs, breath controllers or electromyography (EMG).

### 1.3 Asymmetry and marginal reliability

**Fig 1:**
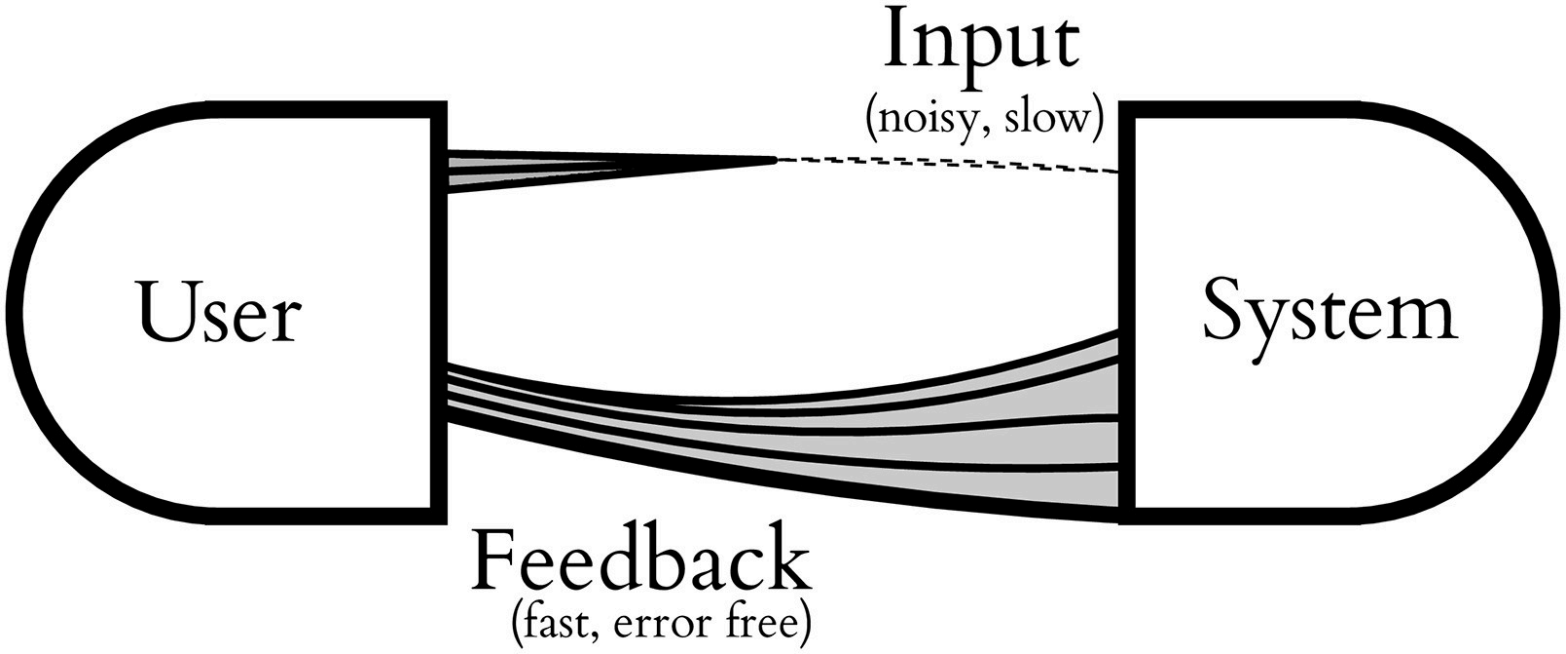
Asymmetric user interface. An asymmetric user interface, where there is slow, unreliable input coupled with a high-capacity, error-free feedback channel; e.g. a noisy switch with a visual display.

We will tackle the problem where we may assume input involves unreliable, low-bandwidth control signals, but there is an essentially perfect (error-free) feedback path. This is typically a visual display where there is negligible error in the perception of the display, and the bandwidth of the display dominates the bandwidth of the limited control path. Such **asymmetric** human computer interfaces require specific design [13] and there are many niches in assistive technology where input is hampered, but perception is not. Fig 1 illustrates this type of interface. We concentrate on constructing interfaces for binary (two-class) systems where the binary accuracy is between 95 and 65% (i.e. bit flip probabilities or bit error rates (BER) are in the range *f* ∈ (0.05, 0.35)). We refer to these as **marginal reliability** channels. In [5], a minimum accuracy of 80% is suggested as a bound for usable interaction. This excludes many input devices.

#### 1.3.1 Example: Motor imagery BCI

Motor imagery (MI) EEG is a widely-used paradigm for non-invasive non-evoked BCI [14–17]. It is a prime exemplar of the **marginal reliability** input device. In this paradigm, users imagine moving different parts of the body and the corresponding event-related rhythm changes in the motor cortex are detected in the electrical signals measured at the scalp. The lateralisation of motor function in the brain leads to a spatial separation of imagined motions which can be classified [18, 19]. Even with modern techniques for feature selection and classification, a motor imagery BCI can typically produce binary decisions at the order of one per second, with accuracies of around 60-90% [2] being typical. One minute of input might produce 60 binary decisions, 10 of which would be flipped.

**Fig 2:**
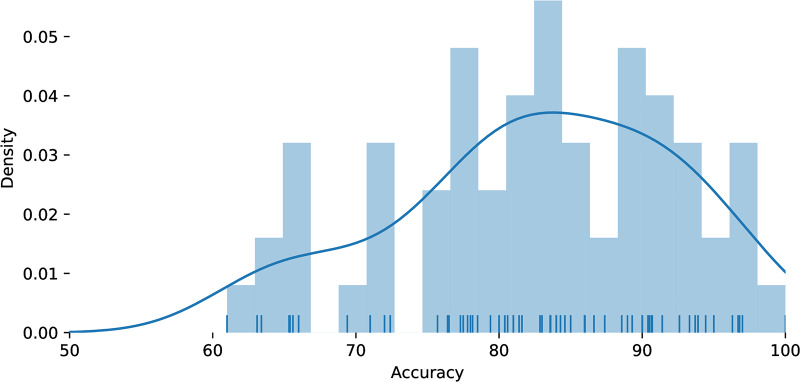
Non-invasive BCI accuracy summary. Summary of per-classification accuracy from many binary non-invasive BCI EEG studies, plotted from the Tables A1-A3 of Lotte et al. [22], including movement intention and mental task imagination BCIs. This figure summarises the accuracy of classifiers used in a large number of brain-computer interfaces, indicating that accuracies vary widely from around 65% to 95% (i.e. there are error rates of 5% to 35%). Where ranges or bounds were given the stated numerical value is used.

Ahn [2] reviews motor imagery BCI systems and and finds error rates reported in the range 35% and above; Ahn [20] illustrates the very high variation in inter-subject error rates with the same classifier from ∼50% to less than ∼5%. Padfield et al. [21] give example error levels from the literature of 9% for visually evoked potentials; 13–31% for event-related potentials; 16% for motor imagery BCI. Lotte [22] reviews per-decision accuracies in the literature for a broad range of non-evoked BCIs; the key results of which are summarised in Fig 2.

There are many otherwise promising technologies which have very high error rates; for example EMG systems with error rates of ∼30% [23]; or 8.1-5.6% [24]. Such high levels of error, combined with frustratingly slow response times make conventional “undo” functionality insufficient (see 4.1.1 for numerical simulation results and the theoretical analysis in [5]). The binary motor imagery channel is an excellent exemplar of the class of niche interaction methods we are interested in, for two reasons: it is a well-known input mechanism for which improved interfaces could offer immediate benefits; and it is an instructive example of designing for extremely challenging input devices. We do not always have the luxury of improving the qualities of channels to be harnessed for input, and it seems likely that current non-invasive EEG-based techniques will have a substantial subset of users for whom two-class motor imagery classification accuracies will be less than 95%. Beyond brain-computer interfaces, marginal reliability channels can be found across systems where input is impaired either physically or situationally and the control akin to a noisy two-state button is the only functionality available.

## 2 Theory

We first develop a theoretical model of error-tolerant user interfaces for marginal reliability channels. We examine the origin of errors and review established error correction techniques in human-computer interaction, and reported error rates of standard user interfaces. We then derive desiderata for widely-applicable interaction mechanisms that can tolerate consistently high error rates. These form the design objectives for our approach. The engineering of error tolerant interfaces is fundamentally a problem in information theory and we present a model from an information-theoretic perspective that maps classical communication concepts of entropy, channel and line coding onto interaction design. We illustrate how conventional user interface elements can be understood from this stance, and how a user in a closed-loop can effectively “code” for the interface channel without mental effort at the cost of becoming tightly bound to feedback.

### 2.1 Error in human computer systems

We will consider an *input error* to be a change of state in a computer system which is incompatible with a user intention; for example a “touch down” event being emitted over a GUI target a user did not want to tap or a key press being registered that did not correspond to text a user was trying to enter. The treatment of errors in a human-computer system is complicated by the hierarchical **layering** of interface functionality and error recovery, as discussed by Nielsen [25]. For example, a mis-click of the mouse is an error at the mouse targeting layer (Nielsen’s *physical layer*), but may not result in an error at some higher layer (like deleting a file, at Nielsen’s *goal layer*), because of an intermediate correction step like a confirmation dialog. Similarly, raw BCI classifiers are not typically “hard-wired” to motor actuators on a wheelchair but instead are mediated by some interpretation or shared control process [26, 27]. This paper sets out a *general* intermediate layer that can be placed between an input device and a higher layer and achieve any desired error rate at that layer with a bounded performance penalty.

There is a well-developed theory of errors in human computer interaction [28–32], and as Wood and Kieras [29] note, “*designing for human error should… be pervasive*”. Key questions to design for error are:

- How do errors arise and what are their causes?
- What strategies exist to mitigate them?
- What typical level of errors are encountered in established interactions?
- What level of error should be designed for?

#### 2.1.1 Classification and origin of error

Avizienenis et al. [28] outline a detailed taxonomy of **errors** (a deviation from intended state) and **faults** (the proximate cause of an error) in the context of safety-critical systems. In this work, we are focused on errors that arise because of natural phenomena, human action, or hardware deficiencies. We do not consider robustness to malicious, deliberate or adversarial actions, or robustness to enduring design and implementation deficiencies in the software itself. We also exclude from consideration enduring cognitive or perceptual issues, such as inability to identify targets or inability to form short-term memories.

In particular, we consider:

- **cognitive errors** such as slips [33, 34], defined by Norman [33] as“*a form of human error defined to be the performance of an action that was not what was intended*”. These are errors in cognition, such as forgotten actions, mis-ordered sequences of action, mode identification errors or incorrectly repeated actions.
- **performance errors** [6]: random variation internal to a human user during the production of motor action, such as poor coordination, or muscle tremor that leads motor action to deviate from intent;
- **environmental disturbances**: unrelated variations external to both a user and a system, such as power fluctuations, lighting variations, or external movement (e.g. vibration inside a vehicle) that pollute control signals; and
- **measurement noise**: distortions originating within an observation system caused by sensing or processing inadequacies, such as the effect of electrical noise, occlusion, quantisation, insufficient classifier training, mis-calibration, etc.

All of these error sources are distinct in nature, but from the perspective of human-machine control similarly lead to deviations between user intention and system state during an interaction. We focus on implementing robustness to *transient* errors caused by essentially random deviations, usually though not necessarily fully independent in time. In particular, we may encounter errors correlated in time in measurement noise (e.g. a sensor getting stuck due to loss of contact) or cognitive errors such as slips which introduce error over several interaction steps (e.g. a user starting a sequence of actions to perform one task, before realising the task was incorrectly chosen).

#### 2.1.2 Mitigation strategies

In the presence of input error, mitigation strategies can be categorised [28]:

- **Rollback** or backward error correction [31]: the system reverts to a previous state; this is the **undo** or **backspace** operation and requires either automatic **error detection** or explicit **correction actuation**. Sometimes this includes larger scale correction strategies [32, 35] such as **cancel/abort** to revert a higher-level task or **stop** to cease execution of a higher-level task.
- **Rollforward** or forward error correction [31]: errors are ignored and state changes anyway. This is appropriate when the cost of an incorrect choice is smaller than the cost of correction. For example, accidentally hitting the volume up control on a music player might change the volume slightly but be of little consequence to the user.
- **Compensation**: the system has sufficient redundancy that errors in input, up to some level of tolerance, do not lead to deviations in internal state. This is the domain of error correcting codes.

Various forms of undo have been extensively studied in human-computer interaction [35–37, 37–40] as a widely implementable way of establishing error tolerance. This typically involves choices about the granularity of undo [38, 40], the structure of undo (linear/branching) [41] and the controls for actuating undo. Other approaches have looked at structuring the finite state machines (FSMs) that define interface behaviour such that they that simply admit fewer errors or are at least harder to drive into erroneous states [42, 43].

Our focus is on the **compensation** strategy via error-correcting codes that introduce exactly enough tolerance to random deviations (for a given input channel) that the internal state remains consistent with user intention. We also examine how to integrate this model with undo-style **rollback** approaches.

#### 2.1.3 Typical error rates in standard interfaces

We will use the term **standard interface** to collect together common, widely used interfaces: mouse pointing on a desktop GUI; typing on a physical keyboard; tapping on a touchscreen GUI; and typing on a virtual keyboard.

Targeting errors in mouse pointing in controlled tasks has been found to be relatively constant around 3% for visual targets from 0.83 to 183mm [44]; studies of mouse pointing in realistic desktop GUI situations found error rates of 3% [45] and between 2-20% [46], with the higher rates for an elderly population. Pointing tasks that can be modelled by Fitts’ law are often assumed to result in a speed-accuracy trade-off that maintains a 4% error rate [47]; a strong predictive model of error rates in pointing tasks is given by Wobbrock et al. [48, 49]. Large scale text entry studies on physical keyboards [1] have suggested fairly stable correction (i.e. backspace) rates of 6% with 1% uncorrected errors remaining, and 1.17 keystrokes/character (from 136M measured keystrokes). A N = 37000 user study on mobile devices with virtual keyboards [50] found uncorrected errors from were around 2% with 1.18 keystrokes/character, suggesting similar level of mis-keying error. Large scale studies of error rates (mis-targeting error) in touch screen tapping have found to be between 10% (15mm targets) to 30%(9mm targets) from 100M touch events on mobile devices [51].

#### 2.1.4 Target error rates

Shannon’s noisy channel theorem [52, 53] indicates that any arbitrary level of transmission error can be achieved over a channel subject to noise, with some bounded cost in pre-encoding the data. Viewing the human input problem as a noisy channel, we can therefore theoretically mitigate any level of noise to achieve any desired level of reliability. Perfect reliability will induce some penalty in communication, and one consideration is the **tolerable error rate** for a user interface.

The studies on typing, pointing and tapping discussed above have error rates typically around 3-10%. This suggests reducing selection error rates to around the 4% error assumed in Fitts’ law-like pointing tasks [47] will give comparable performance to standard interactions. This assumes a similar distribution of options and similar utility/importance per option as in a comparable standard interaction. For reliability-critical systems, we can target much lower error rates and accept slower interaction; for time-sensitive systems like real-time control of a wheelchair, we can target higher error rates and trade-off increased responsiveness for occasional deviations.

### 2.2 Objectives

What properties should a robust interface for marginal reliability input devices have? We consider five key attributes that an error-tolerant interface mechanism ought to have. These form the objectives of our interaction design.

#### 2.2.1 Universality

A widely applicable approach should be able to couple a wide range of noise levels to activities with a spectrum of desired error rates. For example, transforming a noisy pressure sensor into a selection device with error rates comparable with standard mouse GUI interaction (∼4% error rate); or interfacing a BCI with low classification accuracy to a safety-critical function such as neuroprosthesis elbow extension control [54] when pouring boiling water, where error rates of 1 × 10^−6^ may be required.

#### 2.2.2 Predictability

Evaluating designs with users is expensive. We would prefer to know, or at least bound, the performance of a design in advance of building it. This motivates interfaces for which there are strong, parameterised predictive models. Such models would predict performance characteristics, like error level or entry rate, from basic estimates of the properties of an input device. We show that there are rigorous theoretical approaches to achieve this, and devote significant portion of this work to developing theoretical and numerical predictions validated against human behaviour.

#### 2.2.3 Graceful degradation

An interaction method suitable for a variety of input device error rates should not have cliff-edge performance failures. There should be smooth, parameterisable adjustments to the interaction which can cope with increasing error at a proportional performance cost.

#### 2.2.4 Adaptability

Similarly, the interaction should be adaptable to changes in performance, both at design time (i.e. a parameterised and well-understood performance envelope) and online, during interactions, to cope with changing input conditions. Many user interface contexts, especially those encountered in wet-electrode brain-computer interaction, have input properties that vary strongly with time [55, 56] and an interaction model that can cope with these changes by online adaptation will be more widely applicable.

#### 2.2.5 Simplicity and uniformity

Finally, we would like to have an interaction that is conceptually simple for both users (requires little working memory and minimal mental computation) and designers (straightforward, predictable parameterisation of the interaction). To maximise transferability of skills, interfaces developed using interactions should be uniform in their appearance and behaviour, across input devices (e.g. from BCI to pressure sensor), interaction contexts (e.g. from a media player to text entry) and across levels of reliability (e.g. no special handling for high error inputs).

### 2.3 Information theory

**Fig 3:**
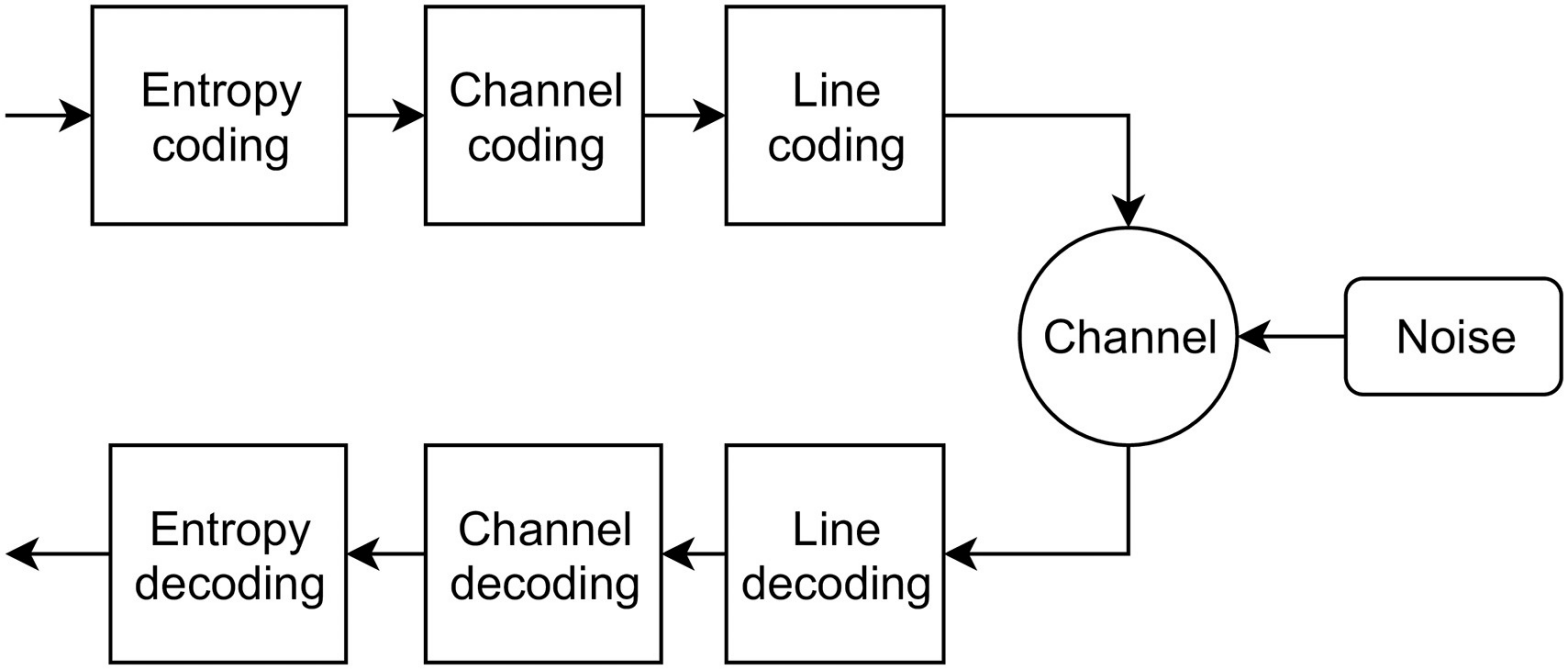
Entropy, channel and line coding. The nested entropy coding, channel coding and line coding stages in a communication channel [60].

The information capacity of a noisy channel is bounded by Shannon’s theorem [52, 57], and there is a vast literature in information theory describing explicit codes for compressing and coding for channels of all types [58, 59]. Optimal transmission on a channel—i.e. passing information through a physical medium—involves three nested stages [60, 61], Fig 3:

- Data to be sent is **compressed** (entropy coded/source coded), exploiting redundant structure to minimise the number of symbols to be transmitted.
- The compressed data is **encoded** (channel coded) such that the effect of noise in the channel can be mitigated.
- The resulting discrete symbols are transformed into an analog channel via a **modulation** (line coding) process.

The reverse process is performed by the receiver, which demodulates, decodes and decompresses the received signal.

#### 2.3.1 Coding in user interfaces

**Fig 4:**
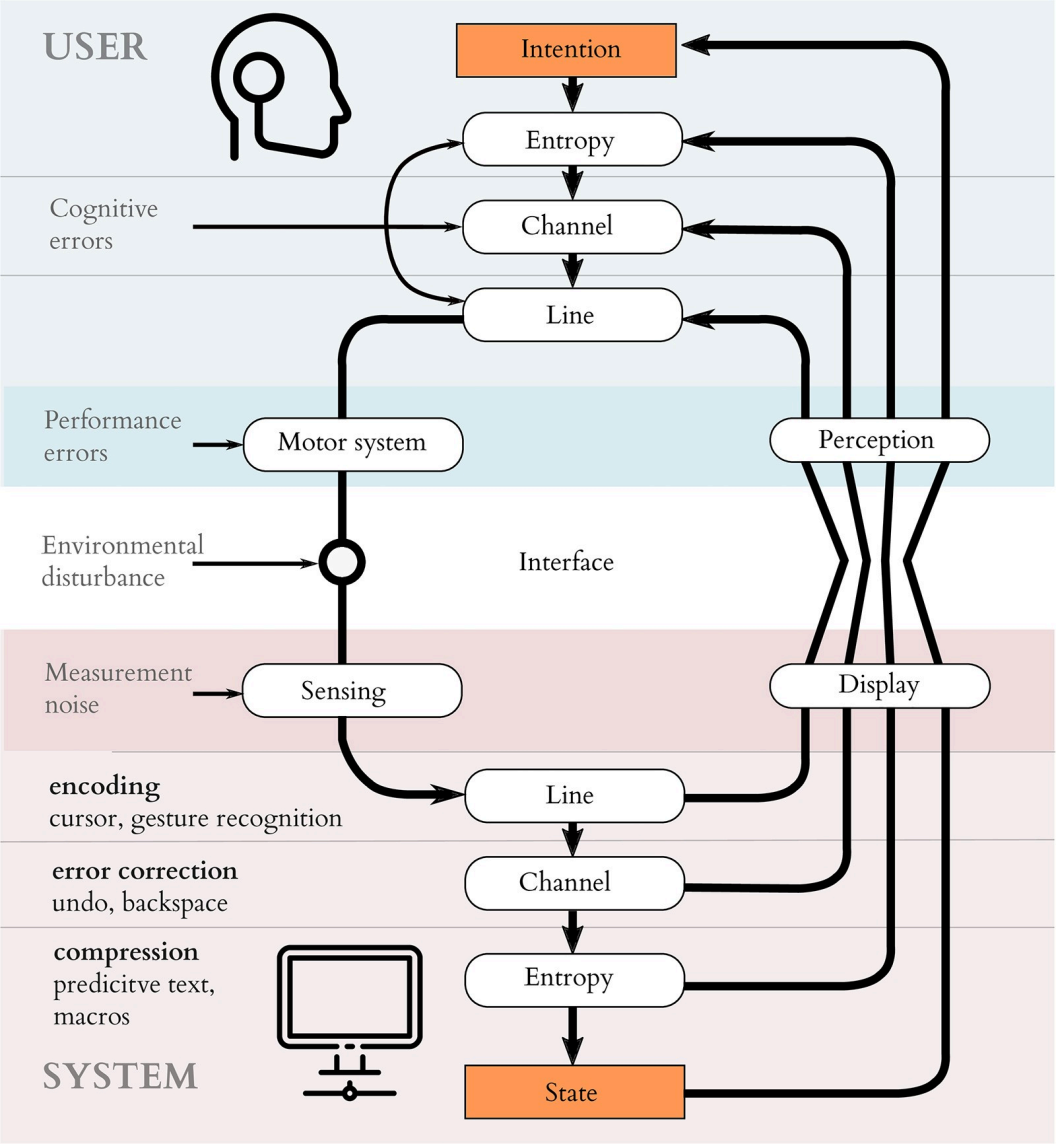
Entropy, channel and line coding in the user interface. The input problem in an asymmetric user interface, viewing the interface communication channel. The diagram shows how the feedback loop allows users to drive the internal state of a system towards their intention, via a nested series of entropy, channel and line coding steps. Sophisticated transport of information across an interface can be implemented by pushing the complexity of the encoding process from the user into the system and relying on feedback to mediate the process.

In the case of a human-computer interface, it is the *user* who must perform the compression, coding and modulation for the channel; the system performs demodulation, decoding and decompression to recover user intention. User interfaces mediate this process by offering feedback which supports the user in these tasks. They can, for example, make the modulation explicit via feedback (as a mouse pointer and a set of targets, for example). They can make the compression explicit (perhaps by offering autocompletions of a partially-entered phrase). Or they can make the error coding process explicit (perhaps by requiring confirmation stages in a sequence of dialogs). Fig 4 illustrates these nested layers of coding for input in asymmetric interfaces; note that the encoding is serial, but the feedback at each level is displayed in parallel via a high-bandwidth noise-free display. We propose to explicitly consider these steps, and their corresponding feedback loops, when designing an interface. Thoughts originating in a users mind must be compressed, by the user, to a small range of state changes available to them. These must be encoded such that they can robustly pass through the noisy processes of the body and the unreliable sensing of the system. They then have to be realised by modulating the physical state of the world; moving a hand, twitching an eyebrow or imagining a foot tapping. The system interpreting those signals must demodulate the sensed physical action, trying to reconstruct the intention the user attempted to signal. This must then be decoded to reliably infer which action was intended; and this decoded action must then be used to optimally select among the many options available according to some probability distribution (decompression).

Designing for human communication is quite different from the issues encountered in traditional communication theory. The physiological and cognitive abilities of humans are very different from those encountered in computer to computer communications and creating user interfaces requires creative engineering to exploit the quirks of human memory and perception. The configuration of joints and muscles, for example, imposes complex ergonomic constraints on the modulation process; even simple spatial targeting has hundreds of variants to optimise the information capacity in different contexts (see Section 2.5).

**The design of a user interface implicitly embodies compression, encoding and modulation**. This is often obscured by the subtle interweaving of these three processes and the layers of metaphor by which interaction designers make interfaces usable, aesthetic and practical to implement, but it is the underlying purpose of a user interface to facilitate communication. The class of interfaces which we are interested in are highly **asymmetric**: rich, high-information-capacity, zero-noise feedback display is available but the input channel is severely restricted.

### 2.4 Assistive technology user interfaces

Many conventional assistive technology interfaces have a relatively high *theoretical* bandwidth (from the Shannon bound), but are very much slower in practice when performing real tasks such as text entry. This is a failing of interface design. Our goal is to enrich the interface by clever feedback design to facilitate efficient extraction of every fraction of a bit of information from the input stream. Our approach to doing this is to explicitly separate these components and to design an interface following a principled, information-theoretic approach. Interface design must satisfy the needs of users. A strong theoretical underpinning to a user-centered design process offers tailoring of interfaces to user needs and capabilities with confidence that the fundamental interaction remains robust and efficient. The aesthetic and metaphorical design considerations of an interaction can be reliably built upon the functional substrate that our approach establishes. The attributes defined in Section 2.2 like predictability and universality can assist designers in efficiently engaging end-users in the design process. We focus on developing interface mechanisms for **channel coding**, which have been less well developed than advances in line and entropy coding.

### 2.5 Line coding

The line coding of a user interface involves transforming physical state changes like body movements or neural activity into state changes within a computer system. This involves both the physical state changes of the human body (e.g. arm motion) and the sensing of these state changes by an electronic device. This coding needs to preserve dynamics compatible with human behaviour, such that the system’s evolution in time is compatible with human perceptual and motor capabilities. A human input line coder usually includes a continuous time feedback loop to the user with suitably damped dynamics (e.g. smoothed cursor position) and emits discrete symbols.

Simple inputs typically have some form of noise-suppression/damping combined with a thresholding operation and to discretise states, such as a leaky-integrate-and-fire unit and some form of hysteresis (see e.g. [62], Fig 4 or the pressure-sensor control schemes of Ramos et al. [63]). This configuration is often placed after a high-frequency noisy classifier output to render a system controllable.

In 2D and 3D, input from a sensor is often mapped down to a point cursor for **pointing input**; for example, mapping from a dense optical flow image to *dx*, *dy* pointer deltas in a conventional optical mouse [64]. Post-processing is used to filter this to make it compatible with human dynamics via transfer functions [65–67] and temporal filtering [68, 69]. Area-based thresholding (e.g. user interface icons) is used to discretise the input, usually in conjunction with a separate actuation channel like a mouse button. There is extensive work in developing efficient line coding for pointing devices by manipulating control-display ratios (the gain between input and cursor feedback displacement), for example as discussed in [70].

Approaches based on feedback matching/**motion coupling**, such as “pointing without a pointer” [71], Pursuits/Orbits [72, 73] and motion-pointing [74] use principles from perceptual control theory [75] to perform line coding. They rely on the user reflecting displayed motion patterns (e.g. by mimicking the movement of a target), and detect correlation between displayed trajectories and observed state changes. This is a wholly-feedback bound approach to line coding. Motion coupling allows flexible, adaptive mapping of input and feedback channels, but cannot easily support learning of motions.

Other approaches to line coding include **gesture** based systems which map discrete symbols (gestures) into trajectory segments via open-loop movement performance [76]. A recogniser [77–79], which is typically some form of classifier trained on exemplars, attempts to segment these symbols in an unbounded time series (spotting [80, 81]) and discriminate the symbols (recognition). This allows a wider range of motions to be used and is usually implemented without formative feedback. This makes users less bound by the feedback, but can lead to problems in revealing or learning gesture sets [82].

### 2.6 Entropy coding

There has been extensive work in producing interactive systems which explicitly address the problem of designing user interfaces to facilitate transparent **entropy coding** by developing probabilistic selection methods with strong priors over outcomes (for example, from predictive language models.) Many of these probabilistic interfaces have been based on a spatial zooming paradigm, starting with Dasher [83]. Dasher used an arithmetic coding approach to subdivide a unit interval, where the interval has area widths allocated according the probability of symbol sequences drawn from some alphabet. In Dasher, these probabilities were derived from language models which predicted subsequent characters given prefixes. These ideas were extended to brain computer interfaces [84], single switch interfaces [85], hybrid speech and zooming based interfaces [86], among others. Similar ideas based on spatial representations of probability distributions were explored in BIGNav [87, 88] which applied Bayesian updating to efficiently zoom in on spatial layouts with a known probability distribution over targets. In cursor-based interfaces, “intelligent pointing” approaches which dynamically manipulate the control-display ratio such as [89, 90]*combine* line coding and entropy coding. By increasing the control-display ratio over regions which are unlikely to have relevant targets, an implicit prior distribution over targets is defined. Information theoretic models for design of interface finite state machines (e.g. hierarchical menus) have also been explored, as in the Huffman-coded menus of [91] which used frequency as a proxy for probability of states. This requires careful design to balance the semantic structure of hierarchies against the information-theoretic optimal design. For example, [5] uses the Hu-Tucker entropy code [92] to preserve lexicographic ordering with a slight penalty in throughput.

All entropy coding interfaces come down to a way of representing a prior probability distribution over options in such a way that the input device available can provide evidence to perform Bayesian updates of the probability distribution as efficiently as possible. This involves a trade-off between the efficiency of the update and the complexity of the interface.

### 2.7 Channel coding

Explicitly designed channel codes, error-correcting codes or error-detecting codes, are not widely used in human computer interaction. Designing for errors is often intermingled with line coding, as some form of post-hoc filtering of sensor inputs. Standard approaches to increase reliability at the line coding level include lowpass filtering or moving averages, and various forms of dynamic thresholding, including hysteresis and dead-zones. Instead, error correction functionality is often included as part of the finite state machine (FSM) that drives system behaviour. This often involves introducing transitions to return to previous states in the state machine (“undo”), transitions to fully or partially reset the state or confirmations before transitions with external consequences. Poor design of FSMs can lead to very suboptimal behaviour in the presence of error (e.g. as discussed in Thimbleby’s analysis of FSM properties in user interfaces [42]). Quek [93] explored Monte Carlo simulation to illustrate how poor menu hierarchy design can have extreme effects on the usability of assistive technology systems.

## 3 Definitions and information-theoretic bounds

**Fig 5:**
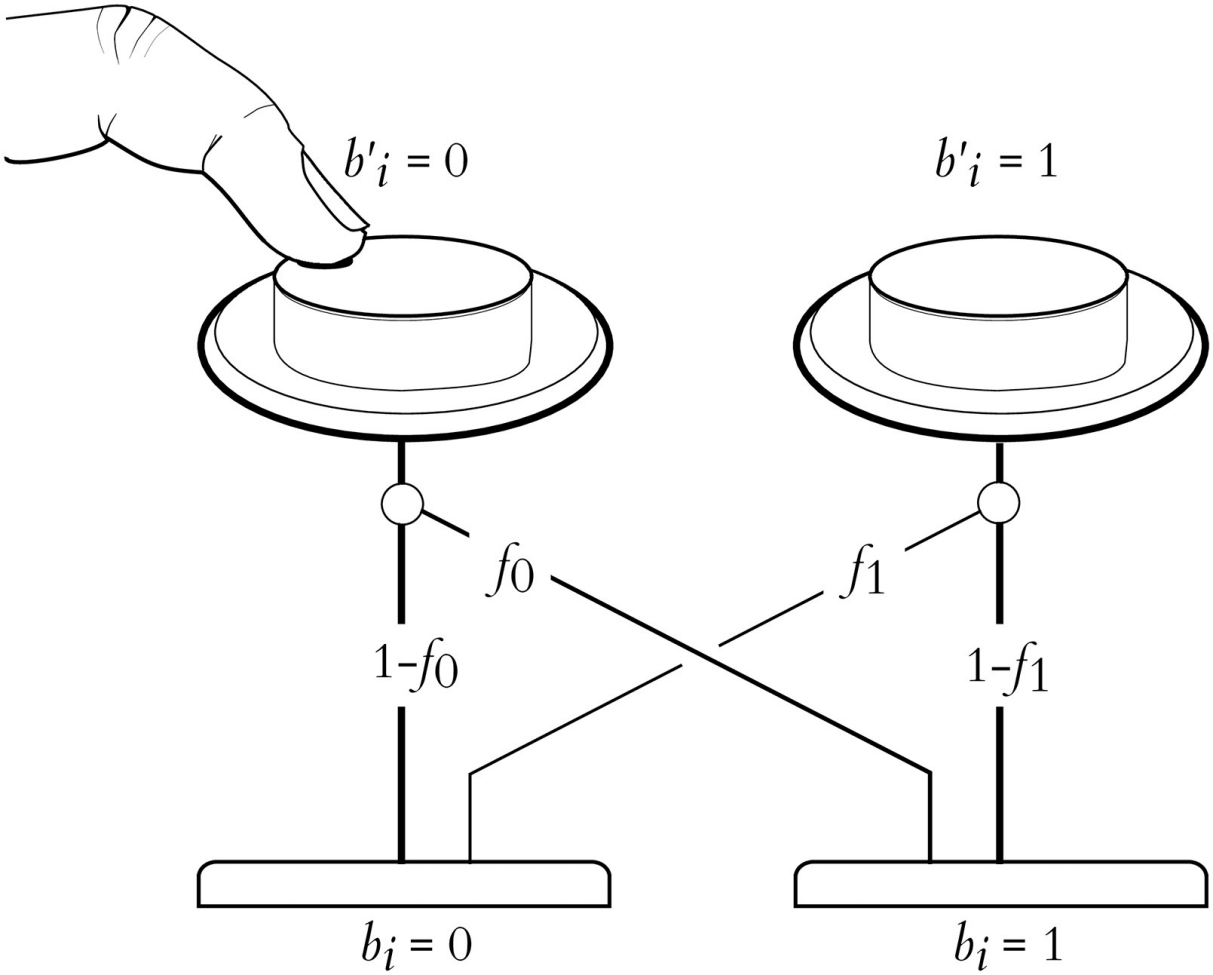
The noisy button model of an interface. An intended input $b_{i}^{\prime }$ is flipped with probability *f*_0_ or *f*_1_, depending on which button was pressed, resulting in the detected input *b*_*i*_.

We now consider the theoretical basis for user interface selection in a **noisy binary channel**. This is a simplified model of an input device where the input is assumed to be restricted to two “buttons” that produce sequences of binary states which are corrupted by random flipping, usually assumed to be independent over time. That is, pressing one of the buttons may result in the signal corresponding to the other button being sensed, and this happens as if from the result of a biased coin flip. The notional buttons may have distinct probabilities of being flipped—one button consistently less reliable than the other—a **biased channel**. This is illustrated in Fig 5.

These “buttons” may be quite abstract: for example, **synchronous** forced-choice controls like classifier outputs from visually evoked brain-computer interfaces [94]; **asynchronous** controls like real physical buttons, or **timing based** mappings like dwell [95], Morse-code style encodings or temporal pointing [96]. The results here can be extended to *q*-ary channels, where *q* buttons are available for input.

### 3.1 Bounds on the noisy binary channel

**Fig 6:**
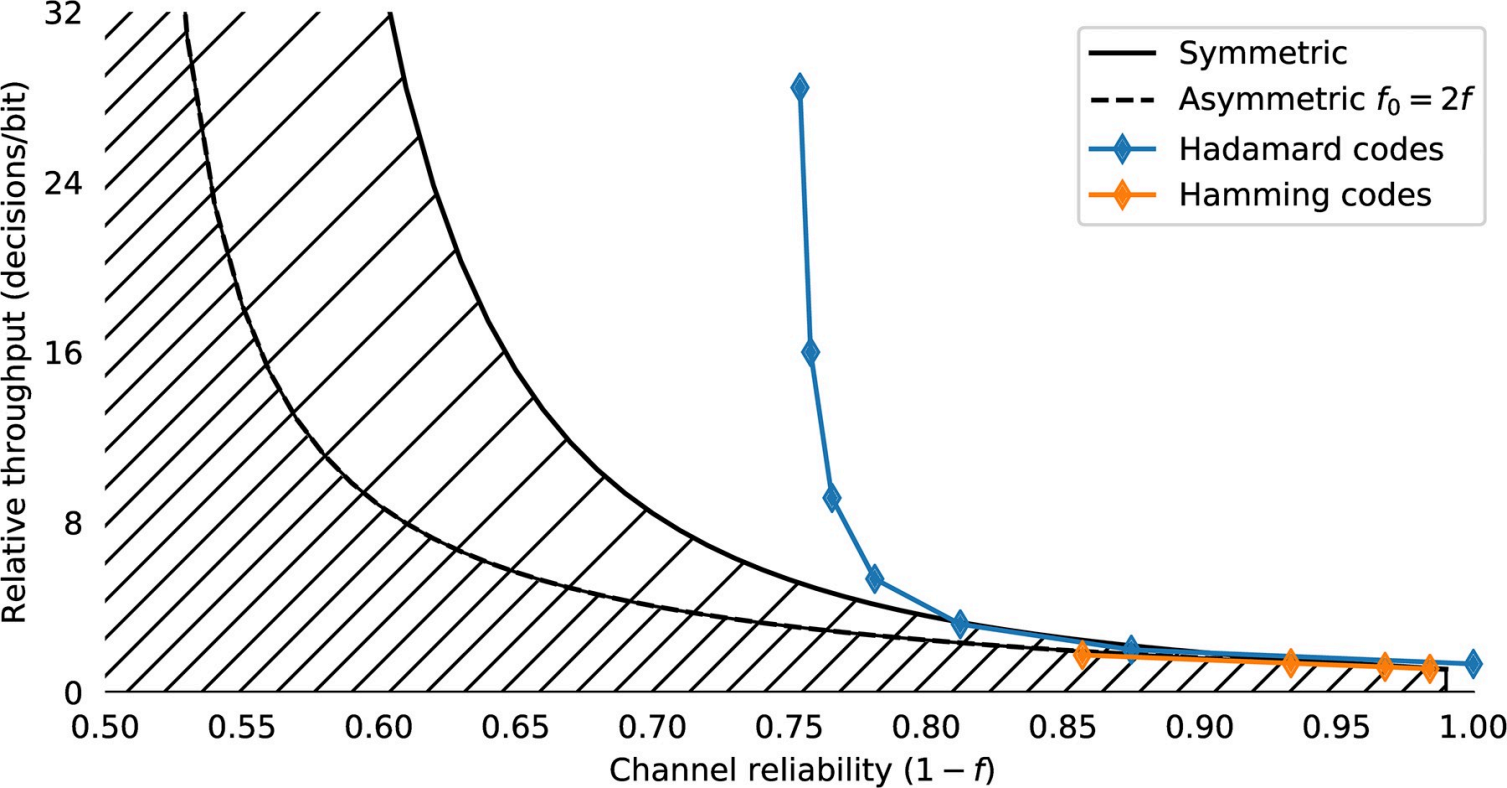
The Shannon bound for the noisy binary channel and classical code performance. The bound is shown in terms of numbers of input decisions/bits per error-free output bit. The upper single hatched region shows the $\bar{R}\left(f\right)$ binary symmetric case *f*_0_ = *f*_1_ = *f*, and the lower double hatched region shows the capacity of the fully biased Z-channel $\bar{R}\left(2f,0\right)$ with the same average error rate (one completely reliable input and one noisy input). Curves for the classic Hamming and Hadamard codes for various word lengths are shown for reference.

**Fig 7:**
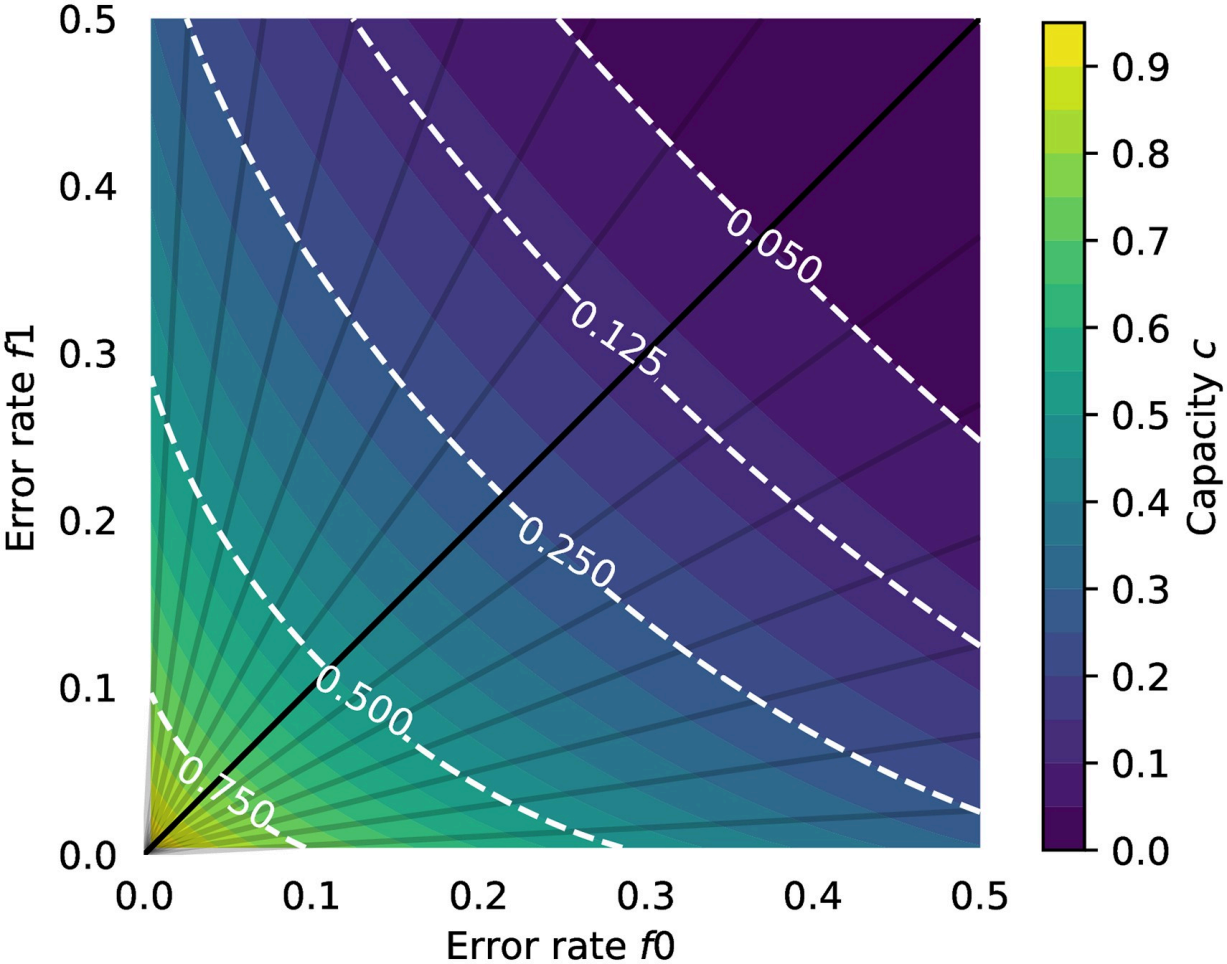
Capacity of the binary asymmetric channel. Different values of error probabilities *f*_0_ and *f*_1_ are plotted with the capacity given by Eq 2. The white contours show lines of constant channel capacity; the dark lines indicate lines of fixed bias *b* ∈ [−1, 1], where *f*_0_ = *f* + *bf*, *f*_1_ = *f* − *bf*.

We begin by deriving the theoretical upper bounds for the **noisy binary channel**. If probabilities of error are equal for both states, this can be modelled as a **binary symmetric channel** (i.e. the input is presented as a sequence of *b*_*i*_ ∈ {0, 1} symbols, and the probability of a 0 → 1 error is equal to a 1 → 0 error), we can find the maximum theoretical capacity of the channel from the binary entropy function given an error probability *f* [52]:

$$\bar{c}\left(f\right)=1-H\left(f\right)=1+f{\text{log}}_{2}\left(f\right)+\left(1-f\right){\text{log}}_{2}\left(1-f\right),$$ (1)

where $\bar{c}\left(f\right)$ is a fraction of the input bits received. However, many real channels are not binary symmetric and exhibit strong bias. For asymmetric binary channels (or Z channel [97]), *P*(0 → 1) ≠ *P*(1 → 0)). The bias can be represented as a term *f*_*δ*_, −1 ≤ *f*_*δ*_ ≤ 1, so that *P*(0 → 1) = *f*_0_ = *f* + *f*_*δ*_, *P*(1 → 0) = *f*_1_ = *f* − *f*_*δ*_, with a constant average error rate *f*. The maximum capacity of the binary asymmetric channel [98] is:

$${\bar{c}}_{\left(f_{0},f_{1}\right)}=(\frac{f_{0}}{f_{\delta }})H\left(f_{1}\right)-(\frac{1-f_{1}}{f_{\delta }})H\left(f_{0}\right)+{\text{log}}_{2}(1+2^{\frac{H\left(f_{0}\right)-H\left(f_{1}\right)}{f_{\delta }}}),$$ (2)

where *H*(*f*) is the binary entropy function, *f*_0_ < *f*_1_, *f*_0_ < 1 − *f*_1_, (which can always be achieved by swapping 0 and 1 as required), and *f*_*δ*_ = 1 − *f*_0_ − *f*_1_. Fig 6 shows the limiting number of input bits per error-free output bit for the symmetric and asymmetric channels for *f* ∈ 0.5, 1.0. The effect of the asymmetry on maximum communication rates is shown in Fig 7. The hatched region of Fig 6 shows the theoretical capacity of a binary symmetric and fully biased channel, in the more user-relevant form of number of input symbols generated per correct bit ($\bar{R}\left(f\right)=\frac{1}{\bar{c}\left(f\right)}$) communicated against the reliability of the channel *r* = 1 − *f*. From a user’s perspective, this is the number of decision processes they need to go through to communicate one binary decision.

We assume a **decoder** which consumes a sequence of input binary symbols [*b*_0_, *b*_1_, …] *b*_*i*_ ∈ {0, 1} randomly corrupted (i.e. a noisy two state button) and produces as output a sequence of *k* bit output symbols [*s*_0_, *s*_1_, …], *s*_*i*_ ∈ *S* from an alphabet *S* consisting of 2^k^ distinct symbols. The decoder receives symbols *b*_*i*_ at a symbol rate of *D*_*b*_ binary symbols per second, and emits decoded symbols *s*_*i*_ at a rate of *D*_*s*_ *k* bits per second. The inputs are assumed to be corrupted by an *independent and identically distributed* (iid) Bernoulli process with a bit flip probability *f* (for symmetric channels) or *f*_0_, *f*_1_ (for asymmetric channels where one button is noisier than the other). The flip probabilities can be estimated empirically $\hat{f_{0}},\hat{f_{1}}$ from some calibration procedure, and a decoder is configured to decode for configured probabilities $f_{0}^{{}^{\prime }},f_{1}^{{}^{\prime }}$. These are typically larger than the expected true probabilities *f*_0_, *f*_1_ to make decoding more robust in varying conditions. The difference between the expected and the configured error rates $f_{h}=f_{0}^{{}^{\prime }}-f_{0}=f_{1}^{{}^{\prime }}=f_{1}$ we call the **headroom** and is typically configured to be positive such that the decoder is pessimistic about the channel noise. We are concerned with the effective capacity of a channel *c*_*j*_(*f*_0_, *f*_1_) with a specific decoder *j*, where the capacity is the fraction of output bits decoded for each input bit. More usefully for user interface design the reciprocal $R_{j}\left(f_{0},f_{1}\right)=\frac{1}{c_{j}\left(f_{0},f_{1}\right)}$ is the **rate** of a specific decoder *j*, the number of input bits to produce one correct bit of output. We write $\bar{c}\left(f_{0},f_{1}\right)/\bar{R}\left(f_{0},f_{1}\right)$ for the theoretical maximum capacity/rate of a channel. The **reliability** of a channel is 1 − *f*, one minus the bit error rate.

- *f*_0_ the probability of a bit flip from 0 to 1, *P*(0 → 1) on a given channel, likewise *f*_1_ for *P*(1 → 0); or just *f* if *f* = *f*_0_ = *f*_1_;
- $\hat{f_{0}},\hat{f_{1}}$ empirically measured flip probabilities (e.g. from a calibration procedure).
- $f_{0}^{\prime },f_{1}^{\prime }$ the flip probabilities a decoder is configured for and *f*_*h*_, the “headroom” $f_{h}=f_{0}^{{}^{\prime }}-f_{0}=f_{1}^{{}^{\prime }}-f_{1}$.
- *b* the relative bias, where *f*_0_ = *f* + *fb*, *f*_1_ = *f* − *fb*.
- *f*_*δ*_ = *fb* the absolute bias, where *f*_0_ = *f* + *f*_*δ*_, *f*_1_ = *f* − *f*_*δ*_.
- *k* the number of bits in a symbol output by a decoder.
- *β* the overhead used by a decoder to confirm a decision (which may be fractional, e.g. *β* = 1.35 bits)
- *b*_*i*_ the *i*th input bit received.
- $b_{i}^{\prime }$ the *i*th input bit intended (i.e. the uncorrupted input).
- *s*_*i*_ the *i*th output symbol of *k* bits from *s*_*i*_ ∈ *S*, the set of output symbols, |*S*| = 2^*k*^.
- *d*(*s*) the function mapping symbols *s* to the unit interval.
- *c* or the capacity of a channel with a specific decoder.
- *e*_*k*_ the error rate, proportion of *k* bit symbols decoded incorrectly.
- *R* the rate of a channel, as number of input bits per decoded bit $R=\frac{1}{c}$
- *R*′ the rate of a channel, after backspace correction to produce error free output
- *D* the input bits/second; and *T* the time for an input bit $T=\frac{1}{D}$.
- *D*_*s*_ *k* bit symbols/second and *T*_*k*_ the time for each output symbol $T_{k}=\frac{k}{D_{s}}$.
- *f*_*i*_(*x*) the probability density function over the unit interval at step *i*; *F*_*i*_(*x*) the cumulative density function
- $f_{i}^{-1}\left(x\right)$ and $F_{i}^{-1}\left(x\right)$ the inverse (cumulative) probability density function
- *m*_*i*_ the median of the probability density function *m*_*i*_ = *F*^−1^(0.5)

### 3.2 Feedback and feedforward

Shannon’s result shows that reliable communication over a noisy channel is possible with only a bounded overhead. It can also be shown [53] that the provision of a feedback channel does not affect the capacity of a noisy channel; there are feedforward codes which achieve just as good performance. However, Shannon’s result is only true as the code block length *k* goes to infinity. It is not feasible for a human to perform actions based on histories of thousands of previous decisions. For practical human-computer interfaces, block lengths need to be very small (on the order of a few bits at most) compared to block lengths required for efficient performance from modern feedforward codes, which might be thousands of bits. Short feedforward codes that are viable for human interfaces have poor performance for channels with *f* > 0.1. For example the classic Hamming [7, 4, 3] code can correct one error in every seven bits (*f* ≈ 0.14) at a cost of 1.75 decisions/bit [99]; the generalisation to Hadamard codes of the family [2^*k*−1^, *k*, 2^*k*−2^]_2_ have large overheads but can correct errors up to *f* ≈ 0.25, though at severe throughput penalty (e.g. Hadamard code [32, 6, 16] used on Mariner 9 achieves a fixed 5.33 decisions/bit). These classic forward error correction (FEC) codes are shown in Fig 6. Modern FEC codes, like turbo codes, LDPC or Reed-Solomon codes [58] have block lengths that are impractical for user interface purposes.

Although the availability of a feedback channel does not increase the capacity of the forward channel, it does dramatically reduce the block length required for efficient communication. If the feedback channel is noise-free (or effectively so) then there are feedback codes which closely approach the Shannon bound with very short block lengths. The combination of a very unreliable forward channel with a high-capacity feedback channel is unusual, but assistive technology interfaces have just these characteristics. Feedback codes allow the coding process to become transparent to the user, without requiring any memory or mental computation on a user’s part, because the state of the decoder can be updated incrementally during code entry.

## 4 Feedback coding

There are few hardware communication channels which have a very low-speed, high noise, feedforward and a high-capacity (almost) noise-free feedback. However, in some interface domains, such as brain-computer interaction, there is often a massive asymmetry in the feedforward and feedback channels [13]. Even in non-assistive contexts, the information capacity of the visual system at the level of consciousness is estimated at 100-1000 bits/s [100, 101] while the capacity of the hand is estimated at 15-25 bits/second [102]; an upper bound of 150 b/s for whole hand all-finger gesturing is suggested in [103]. A visual display can transmit a large quantity of information very quickly, with potentially negligible error, and we can in practice treat it as a noise-free feedback channel.

### 4.1 Backspace and undo

The simplest feedback error-correction approach is to introduce a “backspace” symbol which undoes or removes the previous symbol. This is a feedback error correcting code, and we can easily simulate its performance. The backspace channel works well until the probability of accidentally removing an intended symbol, or emitting a symbol instead of backspace dominates the entry process and a correction cascade occurs. The numerical simulations shown in Sec. 4.1.1 illustrate why backspace or undo-like actions are ineffective at higher error rates. Many systems *only* support this mode of error correction, which explains their cliff-edge performance drops when binary symbol reliability drops significantly below 90%. Given the typically encountered error rates of 3-10% (Section 2.1) in standard interfaces this form of correction is well suited and extremely efficient (e.g. with a keyboard-like input with 63 options + backspace, backspace correction is extremely close to the Shannon bound until error rates increase above 6%, at which point it catastrophically fails).

In systems like hierarchical menus, there may be multiple types of undo (for example, “go back” versus “reset to start”); similarly, text correction may offer single character, single word or whole entry removal via different commands. The application of these correction approaches to brain-computer interaction is discussed in [93], which illustrates how poor choices can lead even relatively reliable input to frequent uncorrectable error cascades.

#### 4.1.1 Simulating backspace

**Fig 8:**
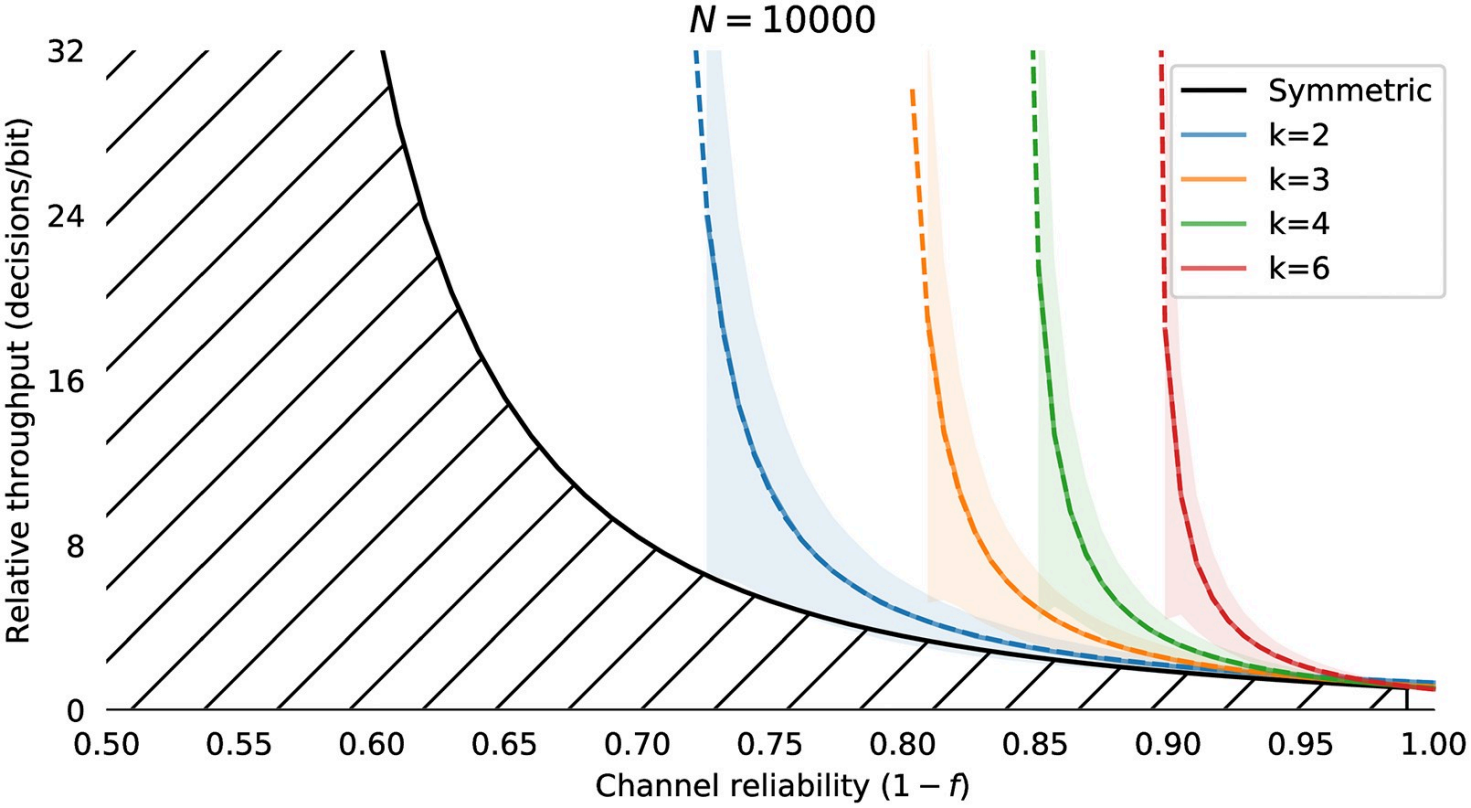
Capacity of the binary symmetric channel with backspace *R*_*b*_(*k*, *f*). Capacity of backspace for alphabets with *k* = 2, 3, 4, 6 for *N* = 10000 simulated entries of a *n* = 32 symbol sequence. Throughput is shown as the number of input bits per correct bit *R*; solid lines shows the mean throughput, and the shaded region shows the standard deviation. The hatched region is the Shannon bound for the binary symmetric channel. Even with *k* = 2 bit symbols, capacity goes to zero as the reliability drops below 75%. Dashed lines show the fit of Eq 3.

We consider the problem of entering a sequence of *n* symbols, using an alphabet *S* of size 2^k^, where *S* = *s*_1_, *s*_2_, …, *s*_←_. One symbol is backspace *s*_←_, and 2^*k*^−1 are unique terminal symbols. Decoding the backspace symbol “undoes” the previous symbol decoded. The performance of this backspace channel can be characterised as the number of bits required to perfectly enter a string of *n* symbols for a given alphabet size 2^k^. The number of binary decisions required from the user to produce one correct bit with backspace coding on a binary symmetric channel is *R*_*b*_(*k*, *f*) with bit flip probability *f* and residual error *e*_*b*_(*f*, *k*) = 0. Empirical results for simulated backspace-entry from *N* = 10000 random trials for various values of *f* and *k* are shown in Fig 8. The decisions per correct bit for the backspace channel *R*_*b*_(*k*, *f*) is approximated by a Gamma function (Fig 8):

$$R_{b}\left(k,f\right)\approx \delta_{k}\left(\Gamma \left(2p_{k}-1\right)+1-p_{k}\right),$$ (3)

where $\delta_{k}=\frac{2^{k}}{2^{k}-1}$, the cost of assigning one of the symbols to backspace and *p*_*k*_ = (1 − *f*)^*k*^, the probability of correctly entering one *k* bit symbol correctly given a bit error rate of *f*. In regimes where the backspace decoder does not function this formula gives negative values which we treat as infinite. From numerical simulation, the mean absolute error of this approximation is approximately 3% for *f* ∈ [0.0, 0.5], *k* ∈ [2, 16]. It is quite clear that although introducing a backspace character makes entry on an unreliable channel *possible*, the performance is very far from optimal, and works very poorly indeed for *f* > 0.1. Even if we settle with entering from four symbol alphabet *S* = {*A*, *B*, *C*, ←}, channels with error rates *f* > 0.25 have effectively zero capacity. This is on top of the mental effort the user must apply to remap those three symbols onto the desired input. Unfortunately, this is often the only error correction available in many assistive technology systems, either as a literal backspace in text entry, or an undo functionality in a more general user interface context. Our problem is to create an input metaphor with a rate *R*(*k*, *f*)>*R*_*b*_(*k*, *f*) for *f* > 0.2, and thus make usable interfaces for channels with reliabilities in the 60%–80% range. As well as the strictly limited range of *f* for which a coding with backspace is useful there are several other issues which make backspace a restrictive error correction technique:

- There is no obvious way to deal with Z channels, with *f*_0_ ≠ *f*_1_.
- Users must alternate between entering symbols and editing to correct errors. These are distinct tasks which require separate mental attention and can become frustrating as errors increase.
- For low-reliability channels (*f* < 0.1), the only effective control has three symbols plus backspace. In most cases users have to concatenate two codes: first a mapping to three symbol+backspace and then onto some higher level symbols such as characters.

#### 4.1.2 Predicting error-free rates from non-zero *e*_*k*_

If we devise a new channel code with some residual uncorrected error rate *e*_*k*_, we can always augment it by adding backspace to reduce the error to zero, if *e*_*k*_ is low enough. We concatenate the inner code with the backspace code. We can predict the number of bits/error-free symbol for this concatenated decoder using Eq 3:

$$R^{\prime }\left(k,f\right)=R\left(k,f\right)\delta_{k}(\Gamma \left(2\left(1-e_{k}\right)-1\right)+e_{k}).$$ (4)

### 4.2 Horstein’s algorithm

Horstein [3] showed a simple and efficient error correcting code for binary channels where a noise-free feedback channel is available. In [104], a discretization of this code was developed, creating “back off” trees for undoing previous steps. This code is much more amenable to static analysis, but is less efficient than Horstein’s original code. A code very similar to Horstein’s is described in [105], and a generalisation to an entire class of codes including Horstein’s, termed **posterior matching feedback schemes** is given in [106] and also proves that Horstein’s code is optimal for discrete memoryless channels—no other code can exceed the rate of Horstein’s code where there is an unlimited noise-free feedback channel. These posterior matching feedback schemes are the fundamental basis of our interfaces.

#### 4.2.1 Optimal noisy bisection

From the point of view of a user, Horstein’s code is a generalisation of bisection to noisy inputs. Bisection is the optimal way to identify a point (within some tolerance) on a bounded interval with noise-free binary input. Options—target symbols that a user might select—are laid out on the interval [0, 1], and there is a “cursor” which divides the interval into two, initially placed at 0.5. Input is sequential, where the user indicates via the input device if the symbol they wish to input is left or right of the cursor. The same approach is used in Horstein’s algorithm, but by accumulating inputs over a whole sequence, the process will reliably converge to an intended target in the fewest possible inputs even when the input is corrupted by random flipping, if the algorithm is configured with knowledge of the true error rate.

#### 4.2.2 Algorithm

A Horstein decoder maintains a continuous probability density over the unit interval [0, 1]. For each step *i* we define:

- *p*_*i*_(*x*) = *P*(*x* = *θ*) The probability distribution for possible values of the unknown target *θ*;
- *f*_*i*_(*x*) the probability density function (PDF) and *F*_*i*_(*x*) the cumulative distribution function (CDF) that define *p*_*i*_(*x*);
- $F_{i}^{-1}\left(x\right)$ the inverse cumulative distribution function.

*F*_*i*_ is stored as a piecewise linear function, and so the probability density $f_{i}\left(x\right)=\frac{dF_{i}\left(x\right)}{dx}$ is a mixture of uniforms.

Typically we begin the process with a uniform prior *p*_0_(*x*)∼*U*(0, 1) but any other prior could be used instead. Algorithm 1 shows the complete algorithm.

**Algorithm 1** Horstein’s algorithm.

1: **function** horstein(*k*, *β*, *f*_0_, *f*_1_)

2:  *p* ← (1 − *f*_0_)/((1 − *f*_0_) + *f*_1_)

3:  *q* ← (1 − *f*_1_)/((1 − *f*_1_) + *f*_0_)

4:  CDF *F*_0_(*x*) ← line segment [(0, 0), (1, 1)]

5:  **while** *H*(*f*_*i*_(*x*)) < (*k* + *β*) **do**

6:   $m_{i}\leftarrow F_{i}^{-1}\left(0.5\right)$ (median from inverse CDF)

7:   Display *m*_*i*_

8:   Receive *b*_*i*_ from input device

9:   **if** *b*_*i*_ = 0 **then**

10:    *F*_*i*+1_[0: *m*_*i*_] ← *pF*_*i*_[0: *m*_*i*_]

11:    *F*_*i*+1_[*m*_*i*_:] ← (1 − *p*)*F*_*i*_[*m*_*i*_:]

12:   **else**

13:    *F*_*i*+1_[0: *m*_*i*_] ← (1 − *q*)*F*_*i*_[0: *m*_*i*_]

14:    *F*_*i*+1_[*m*_*i*_:] ← *qF*_*i*_[*m*_*i*_:]

15:   **end if**

16:  **end while**

17:  **return** *m*_*i*_

18: **end function**

#### 4.2.3 Horstein’s algorithm as a Bayesian update

Horstein’s algorithm process is simply the recursive Bayesian updates of a probability distribution *p*_*i*+1_(*x*|*b*_*i*_) given an noisy input *b*_*i*_ that indicates whether the target *θ* < *m*_*i*_. The median *m*_*i*_, is defined such that $\int_{0}^{m_{i}}p_{i}\left(x\right)=0.5=F^{-1}\left(0.5\right)$. Then we use the distribution at the previous step *p*_*i*_(*x*) as a prior, and the posterior is given by:

$$p_{i+1}\left(x|b_{i}\right)=\{\begin{array}{cc} q_{i}p_{i}\left(x\right) & \;\text{if}\;x<m_{i}\\ \left(1-q_{i}\right)p_{i}\left(x\right) & \;\text{if}\;x\geq m_{i} \end{array}$$ (5)

(Alg. 1 9-15), where

$$q_{i}=\{\begin{array}{cc} P(\theta <m_{i}|b_{i}=0) & \;\text{if}\;b_{i}=0\\ P(\theta <m_{i}|b_{i}=1) & \;\text{if}\;b_{i}=1 \end{array}$$ (6)

(Alg. 1 2-3).

Because we always divide at the median *m*_*i*_, we can assume that there is an equal probability of *θ* < *m*_*i*_ at any step *i*; then the prior *P*(*θ* < *m*_*i*_) = *P*(*θ* ≥ *m*_*i*_) = 0.5.

$$\begin{array}{c} P\left(\theta <m_{i}|b_{i}=0\right)=\frac{P\left(b_{i}=0|\theta <m_{i}\right)P\left(\theta <m_{i}\right)}{P\left(b_{i}=0|\theta <m_{i}\right)P\left(\theta <m_{i}\right)+P\left(b_{i}=0|\theta \geq m_{i}\right)P\left(b_{i}=0\right)}\\ =\frac{(1-f_{0})0.5}{\left(1-f_{0}\right)0.5+f_{1}0.5}\\ =\frac{1-f_{0}}{\left(1-f_{0}\right)+f_{1}} \end{array}$$ (7)

and by symmetry:

$$P\left(\theta <m_{i}|b_{i}=1\right)=1-\frac{1-f_{1}}{\left(1-f_{1}\right)+f_{0}}.$$ (8)

#### 4.2.4 Horstein decoding in the human-machine loop

**Fig 9:**
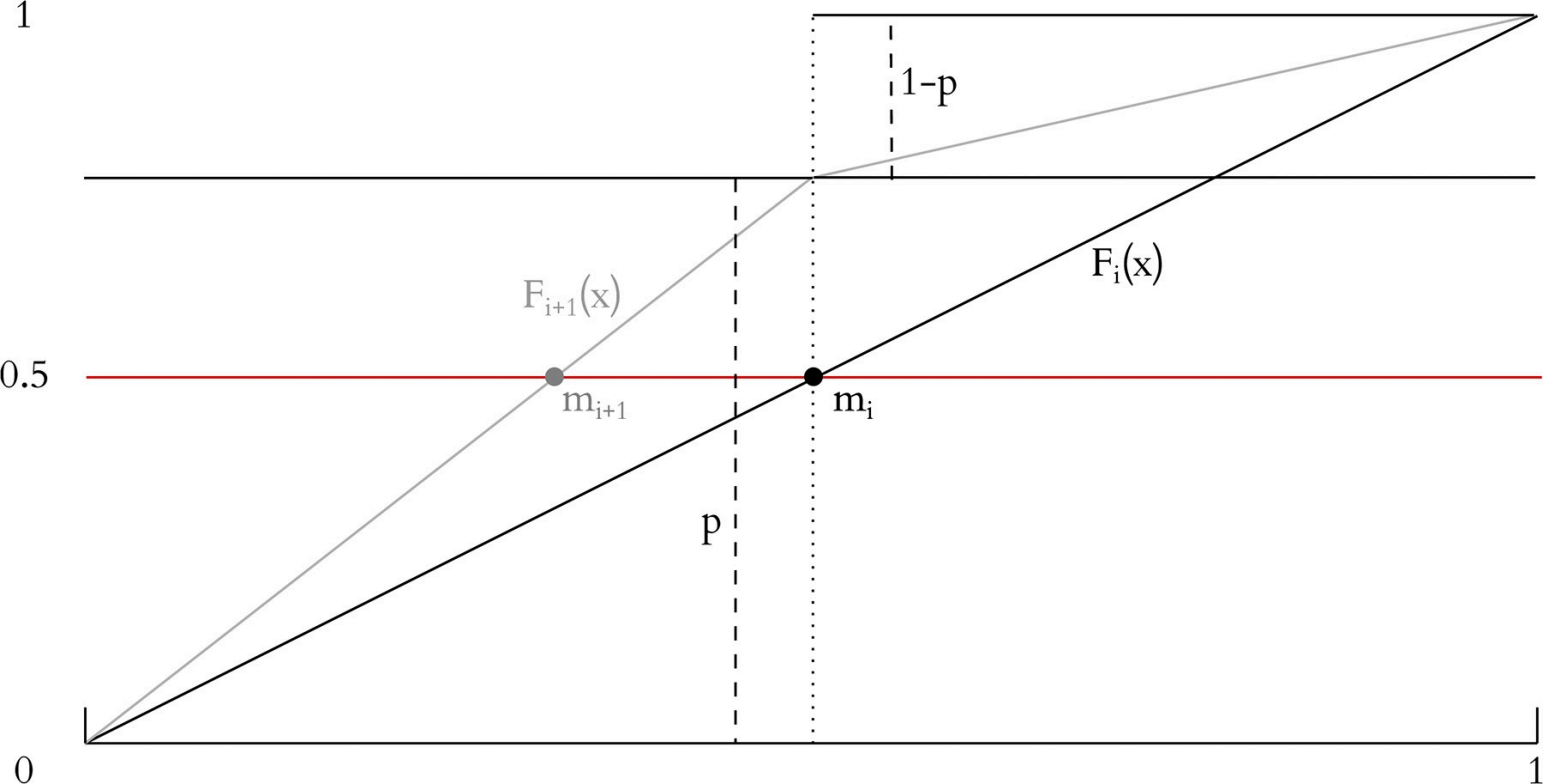
The key step of the Horstein algorithm: Distorting the CDF. The CDF transition is shown for the case where the initial transition is *b*_0_ = 0. The CDF *F*_*i*_(*x*) is initially a line segment with gradient 1. It is partitioned at the point *m*_*i*_ where *F*_*i*_(*x*) = 0.5 and the left and right gradients are scaled by factors *p* and 1 − *p*. In the case *b*_1_ = 1, the respective factors would be 1 − *q* and *q*.

The algorithm elicits a “left” (*b*_*i*_ = 0) or “right” (*b*_*i*_ = 1) decision from the user for each input step, by sending the targets and the current median *m*_*i*_ of the cumulative density function (CDF). The user inputs a “left” (*b*_*i*_ = 0) if the desired target is less than the median, and right (*b*_*i*_ = 1) otherwise. *F*_*i*_(*x*) is then distorted according to how reliable the input is regarded as being. These distortion steps gradually steepen the cumulative density function *F*_*i*_(*x*), or equivalently, concentrate the probability density. Fig 9 illustrates the key update step of the algorithm.

#### 4.2.5 Block coding

**Fig 10:**
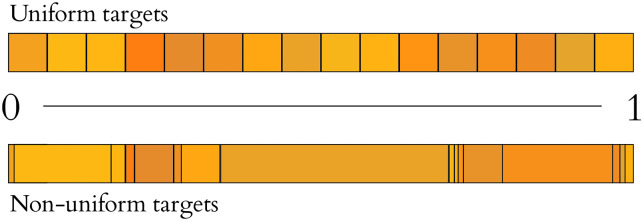
Uniform and nonuniform targets. Subdivision of the unit interval into discrete targets for selection can be performed uniformly (top, corresponding to a flat prior over targets), or according to some known prior distribution *π*(*s*_*i*_) (bottom).

We present a slight modification of Horstein’s original stream code, using fixed length symbols (though in practice we can relax this to variable length codes to accommodate arbitrary priors over targets). To use this code, we choose a symbol length *k*, and an adjustable confidence level *β* (measured in bits). We then map each of the 2^k^ symbols onto an interval in [0, 1] of length 2^−*k*^, i.e. each codeword onto a subsection the unit interval. We can of course introduce a non-uniform prior over outcomes (e.g. as in arithmetic coded interfaces), such that the codewords are then assigned to non-equal sub-divisions of the unit interval (Fig 10).

Because of this change, our termination condition differs slightly from the original given by Horstein, which terminates when a region around the median becomes sufficiently dense. We instead continue until the entropy of the distribution over the interval drops by a set level:

$$H_{i}\left(x\right)-H_{0}\left(x\right)>k+\beta$$ (9)

**Fig 11:**
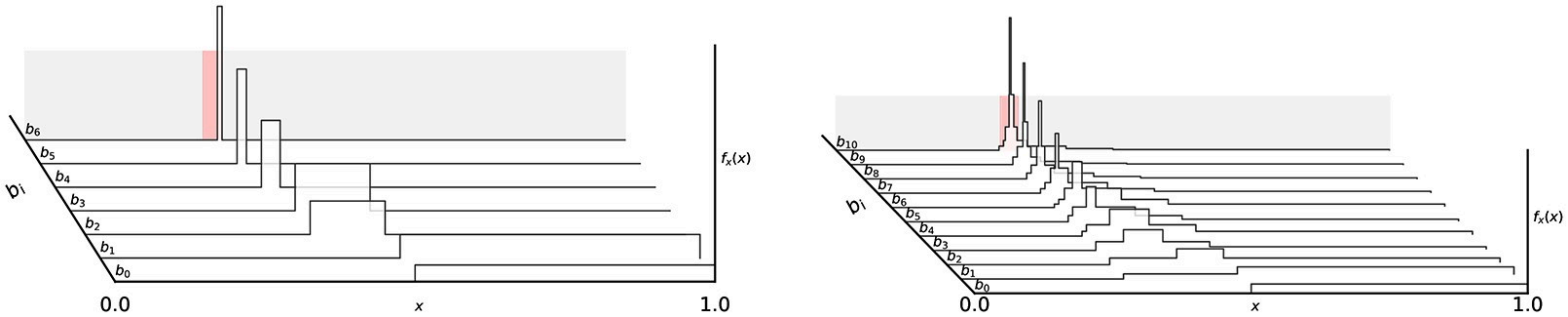
Ridge-plots of the PDFs from the Horstein algorithm. PDFs are plotted following each input bi applying Horstein algorithm to select the the 5 bit symbol 01010 (mapped to the interval around *θ* = 0.3125, highlighted in red), for the noise-free case (left, *f* = 0) and with simulated noise (right, *f* = 0.15), with k = 5, *β* = 2. Sharpening of the PDF is gentler in the case with noise.

At the termination, we now have a new distribution over the unit interval, and consequently over the symbol set. This transformed into a symbol by choosing the symbol whose interval which contains the median *m*_*i*_ at termination. Under a uniform subdivision, given a target symbol *s*_*i*_ of length *k*, we compute *s*_*i*_ = [2^*k*^ *m*_*i*_]. Fig 11 shows an illustration of evolution of the probability density function (PDF) and cumulative density function under the Horstein algorithm with *k* = 5, *β* = 0 for the noise-free and noisy cases. An illustration of how the updating inverse PDF at each step of the Horstein algorithm can be used to remap the unit interval to “stretch out” areas of higher density is shown in the sequence of steps in Fig 13.

The Horstein decoder is only optimal for discrete memoryless channels *where the channel statistics are known*. Section 6.1 presents empirical results which show how the decoder throughput varies against the mismatch between the expected and actual channel statistics.

#### 4.2.6 Biased channels

**Fig 12:**
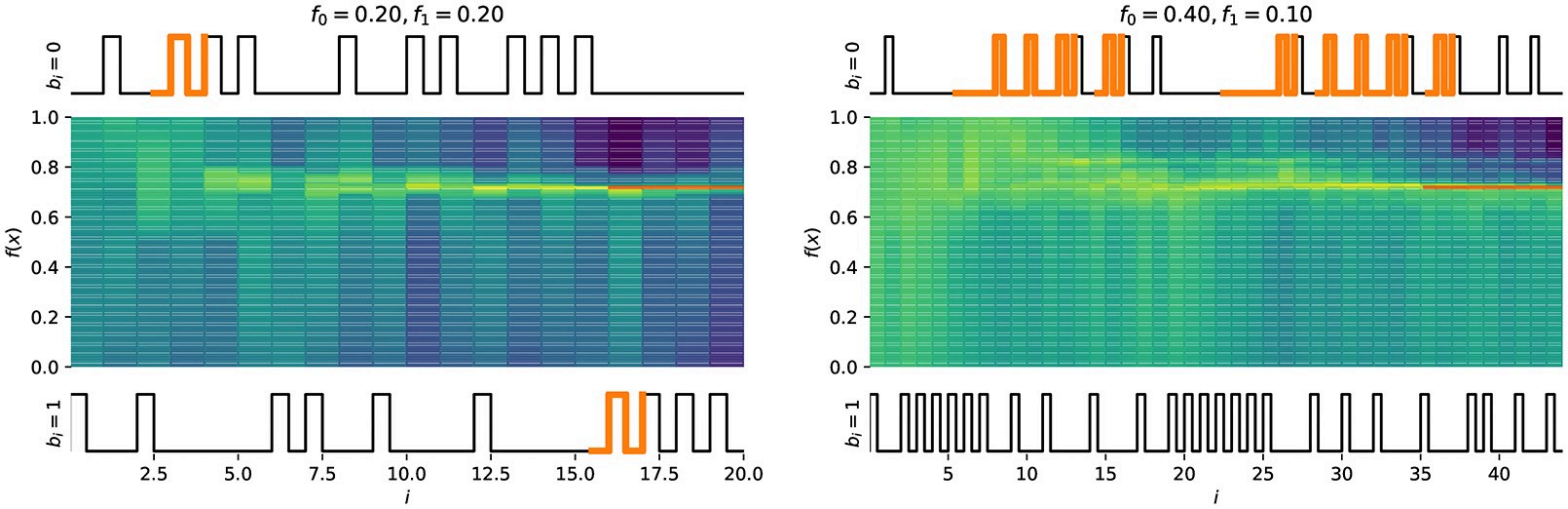
Example simulation of the Horstein algorithm with noisy inputs. *k* = 8, *β* = 0 selecting a target *θ* = 0.71875, with symmetric (left) and biased (right) noise. The pulse traces show the inputs for *b*_*i*_ = 0 and *b*_*i*_ = 1 respectively; highlighted sections indicate erroneous inputs. The centre plot shows the log PDF log*f*_*i*_(*x*) at each step.

If the channel under consideration is not symmetric but is **biased** (i.e. *f*_0_ = *P*(0 → 1) ≠ *f*_1_ = *P*(1 → 0)) the performance will clearly be affected. The bias can be represented as a term *δ*, −1 ≤ *δ* ≤ 1, so that *P*(0 → 1) = *f*_0_ = *f* + *f*_*δ*_, *P*(1 → 0) = *f*_1_ = *f* − *f*_*δ*_, which maintains a constant average error rate *f*. The Horstein algorithm can deal optimally with biased channels. Fig 12 shows simulations illustrating the evolution of the density function for biased and unbiased errors. A general Horstein decoder is fully parameterised by the tuple (*k*, *β*, *f*_0_, *f*_1_).

#### 4.2.7 Headroom

Since we have an imperfect knowledge of the true channel statistics *f*_0_ and *f*_1_, and there is a steep penalty for under-estimating the error rate (see Section 6.3) it is prudent to add some tolerance to the expected channel statistics when setting the decoder’s configured rates $f_{0}^{\prime }$ and $f_{1}^{\prime }$. This **headroom** *f*_*h*_ introduces a penalty in reduced communication rate but in return offers protection against uncorrectable error cascades when the uncorrected error rate *e*_*k*_ slips above the rate that a concatenated backspace decoder can recover from.

#### 4.2.8 Trisection and *q*-ary inputs

In some use cases it is easier to imagine an interface splitting a set into an inner and outer part, rather than bisecting on a central point (for example, consider an interface requiring a motion towards or away from a screen). This can be implemented with the Horstein decoder by trisecting the CDF *F*_*i*_(*x*) at the 25% and 75% percentiles instead of the median, and using the input *b*_*i*_ to either scale the first and fourth quartile or the second and third quartile.

The Horstein decoder extends naturally to *q*-ary channels. Instead of splitting at the median *m*_*i*_ at each step, the splitting is perfomed at each quantile *m*_0_…*m*_*q*_, dividing the CDF *F*_*i*_(*x*) into *q* units. Given a new *q*-ary symbol *b*_*i*_, the slopes of each quantile segment *F*_*i*_[*j*], *j* ≠ *b*_*i*_ are multiplied by 1 − *f*_*b*_ and the slope of *F*_*i*_[*b*_*i*_] is multiplied by *f*_*b*_, where *f*_*b*_ is the expected probability of error for input symbol *f*_*b*_.

#### 4.2.9 Entropy coding

It is straightforward to combine the Horstein algorithm with entropy coded data using arithmetic coding. In this case, we have a non-uniform prior over targets, which is represented as distribution over the unit interval *π*(*x*). We simply continue with the Horstein code in *k* length chunks then output any completed symbols pending.

#### 4.2.10 Decision quality metrics

It has been assumed that only a fixed, unvarying estimate of the channel statistics is available, for example from calibration. Some input devices can report reliability on a per-decision basis (e.g. from a probabilistic classifier), instead of a discrete binary value. The reliability measure can be used to dynamically estimate *f*_0_ and *f*_1_ at each step. In the simplest case, the classifier emits probabilities directly which are used as *f*_0_ and *f*_1_. Other methods (e.g. support vector machine-based classification) may report distance measures from which an (approximate) probability can be derived.

#### 4.2.11 Adaptation

In the simplest case, a calibration procedure with known targets can be used to estimate $\hat{f_{0}}$ and $\hat{f_{1}}$; however, this requires the user to spend time performing this calibration task, or a strong prior model of the channel to be known. In cases where the channel statistics may be unknown, or may change over time, it is possible to adapt the decoder online. This can be done by counting the number of inputs *n* actually required to reduce the entropy to *k* + *β* for each symbol, and compare with the expected inputs for the configured channel statistics using Eq 2, $n_{p}=\frac{1}{{\bar{c}}_{(}f_{0},f_{1})}$. This leads to the adaptive update rule where

$$\begin{array}{c} f_{i+1}=\{\begin{array}{cc} f_{i}+\delta_{n} & \text{if}\;\frac{n-n_{p}}{n_{p}}>\epsilon_{n}\\ f_{i} & \text{if}\;-\epsilon_{n}<\frac{n-n_{p}}{n_{p}}<\epsilon_{n}\\ f_{i}-\delta_{n} & \text{if}\;\frac{n-n_{p}}{n_{p}}<-\epsilon_{n}, \end{array} \end{array}$$ (10)

for some threshold *ϵ*_*n*_, and a small fixed quantity *δ*_*n*_. This provides a simple way to adapt the decoder online for symmetric channels. See Section 6.4.2 for an online adaptation algorithm suitable for biased channels.

### 4.3 Limitations

The Horstein coder is optimal for memoryless channels with known statistics [3]. However, like all error-correcting codes it achieves optimality only asymptotically as the code length *k* increases. The performance of the Horstein code is very good for small *k* < 12, particularly compared to feedforward error correction, and reasonable performance requires *k* > 4 at the least. This means that it must make sense for the user interface to bundle up a sequence of decisions into a choice from a large number of symbols. For tasks such as text entry (where symbols can be letters or words or relatively unbounded sequences, as in Dasher [83]) or spatial selection (where symbols are (*x*, *y*) co-ordinates on a dense grid), or even future trajectory planning (where symbols are sequences of movement commands) this is often straightforward. For tasks requiring real-time intervention or control (such as steering a vehicle in a changing environment), an error-correcting code is less useful, as there are often a small number of options available, and they must be activated at predictable times, a structure which does not lend itself well to channel coding.

## 5 Interface design

**Fig 13:**
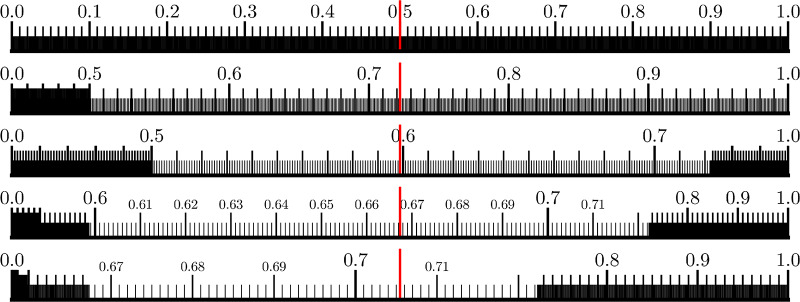
Zooming into the number line. The Horstein algorithm being applied to approach *θ* = 0.71875, with an assumed *f* = 0.1, shown as the inverse PDF *f*′(*x*) distorting a ruler spanning the unit interval. The median mi is shown as a red line.

The inverse probability density function $f_{i}^{-1}\left(x\right)$ expands around the median *m*_*i*_ for each step (this is how the algorithm is presented in Horstein’s original paper). The problem of selecting can be transformed into one of binary control, where the user decides if a target area (representing a codeword) lies to the left or right of *m*_*i*_ on a number line distorted by $f_{i}^{-1}\left(x\right)$ (Fig 13) and produces a decision *b*_*i*_. This decision is fed to a Horstein decoder, which computes a new $f_{i+1}^{-1}\left(x\right)$ and this can be displayed to elicit the subsequent decision *b*_*i*+1_.

This gives rise to an obvious implementation as a (non-uniform) distortion of space. This can be implemented as a type of **zooming user interface** [107–110], where *f*^−1^(*x*) is used to directly distort the display, expanding regions of high probability. Alternatively, non-uniform distortion can be hidden and an interval of fixed density (e.g. the 50% highest density posterior interval (HDPI)), to produce a linear zooming interface.

The zooming approach for the entropy coding was successfully applied in Dasher [83] and is particularly well-suited to building interactions with variable length codes and with hierarchical or sequential decisions. Longer codes imply deeper zooming and there is a particularly elegant representation of entropy indicated the current displayed zoom level; as the decoder becomes more certain, zoom increases; as it becomes less certain, the view backs out. Dependent sequential decisions (coding steps) are visually related to each other through the hierarchy of visual scales. A zooming interface can proportionally dedicate screen space to decisions a user must immediately make, while preserving spatial context about prior decisions and an indication to help a user predict future actions. Animated interpolation between zoom levels can be used to strengthen this spatial context by minimising sudden changes in display.

The result is an interface which gradually zooms in on the region of interest, even when the input is subject to bit flips. The code effectively creates a type of continuous undo which through the memory of previous decisions (accumulated in the CDF at each step) can recover from errors. The zooming effect ensures that the visual resolution varies according to the certainty of the intervals, so that more certain regions have higher resolution.

### 5.1 2D mapping

The coding technique requires that we have relatively long codewords to obtain good performance. In other words, there must be a large number of available options for each decision “bundle”. To design a usable interface using the Horstein decoder mechanism, it must be possible for a target user to be able to identify each of the options available for selection, so that they can decide which side of the median their desired option lies and produce the appropriate binary symbol by flipping a switch, invoking a motor imagination sequence or actuating whatever other input means are available. With a simple 1D display the number of options that are visible is very limited, and is only practical where a user can interpolate between options sensibly (e.g. if the options are ordered numerically or lexicographically). For the more general case, a 2D grid layout provides a much greater display area, but introduces complexities in mapping from the symbol space (and any associated probability distribution) to 2D geometry and in the mapping of a single two-state switch to 2D navigation. We discuss solutions to these problems below.

#### 5.1.1 Multiple dimensions

The decoder can be extended to *N* dimensions by maintaining *N* independent Horstein decoders *C*^0^…*C*^*N*^, each representing a marginal probability distribution $p_{i}^{j}\left(x\right),0\leq j<N$ at decision *i*. As these are independent, we can compute the joint distribution on the *N* dimensional space simply as $p\left(x_{0},x_{1},\ldots ,x_{N}\right)=\prod_{j=0}^{N}p_{i}^{j}\left(x\right).$ Simply cycling through decoders in round robin order is inefficient, because the random distribution of errors may have one decoder almost certain, while others are far from convergence. We propose a more intelligent selection of decoder at each decision, as the feedback channel gives us the freedom to elicit information from dimensions on an arbitrary schedule by changing the display. To select the next decoder to update, we compute the entropies *H*^*j*^(*x*) for each decoder *C*^*j*^, and request input for the dimension with the largest entropy. This entropy-based scheduling can also be extended to multiple input modalities (e.g. hybrid BCI).

#### 5.1.2 Mapping controls: Diagonal split interfaces

**Fig 14:**
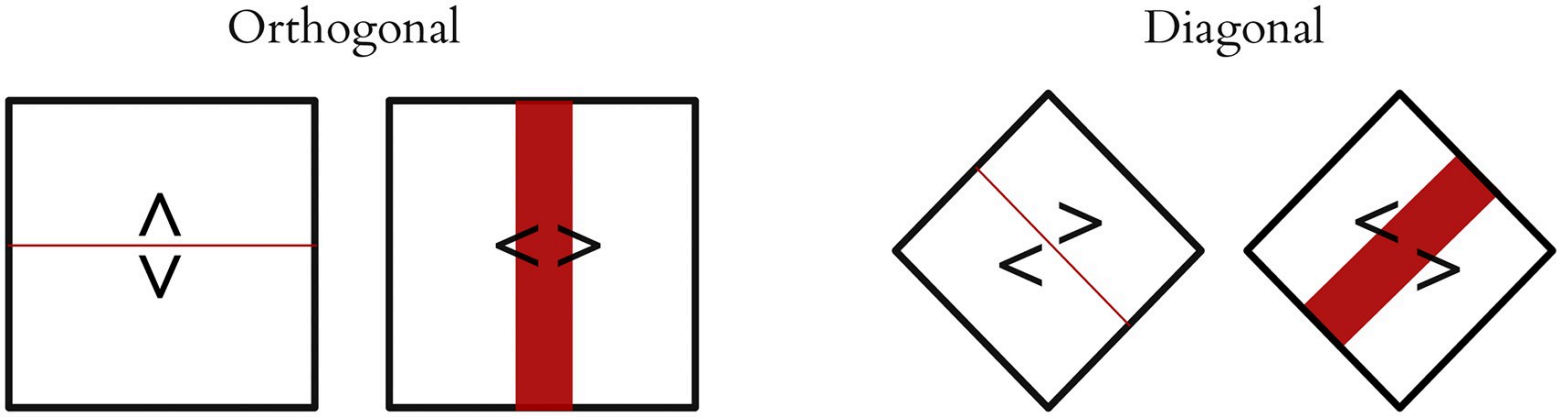
Diagonal split interfaces. Rotating the plane 45 degrees allows partition on both axes with only left-right decisions.

This approach provides a straightforward decoding process for 2D grids (although it is perfectly possible to perform the selection in 3 or more dimensions, a 2D grid is the most practical to display). Multiple options can be laid out on a 2^*k*^ × 2^*k*^ grid, and the system can request input from the appropriate decoder dimension by drawing a median line on appropriate axis. From a user’s point of view, a binary input device provides two options, normally with a strongly associated directional component (e.g. left hand versus right hand). Scheduling dimensions to distinct decoders according to entropy is theoretically efficient, but it requires changing the mapping from the input device to the display every time the decoder switches (switching left vs. right to up vs. down, for example). It is quite challenging to deal with an input device whose interpretation changes regularly, especially for input channels like motor imagery, where the input classes might be left hand imagination and right hand imagination; rapidly switching between a left hand being interpreted as “select the left side” and meaning “select the upper side” makes the input task much more difficult, as we observed in initial early prototypes of our interface. A simple solution in 2D is to rotate the displayed grid 45°, so that every decision is always between left and right, even as the axes alternate (Fig 14). This allows packing options onto a 2D grid, but requiring only left/right decisions which map precisely to the visual display.

### 5.2 Non-linear versus linear visualisation

**Fig 15:**
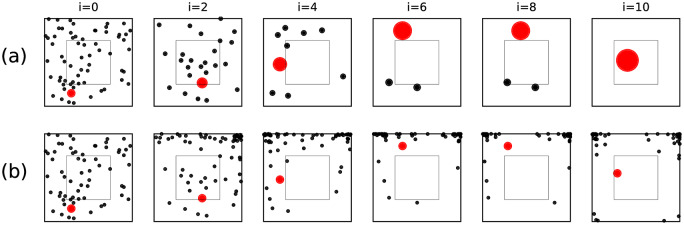
Linear and nonlinear zooming. A 2D Horstein-zooming interface using linear and non-linear zooming displays (every second step shown). Targets are shown as black points, with the intended target shown as a larger red marker. (a) Linear zooming, where the geometry of points is fixed, and the view spans an interval of constant density. (b) Non-linear zooming, where the inverse PDF is directly applied to points in a 2D space, pushing unlikely points to the edges of space, always showing the entire unit interval.

We use a form of zooming interface to represent the state of the decoder. User interfaces based on zooming have a long history in human computer interaction [107, 108] and are a natural fit for the Horstein decoder process applied to 2D selection. There are two ways to represent this display to the user as a zooming user interface. **Linear zooming** computes the highest density posterior interval (HDPI) at some threshold on both axes, and then centres and scales the display around fit this interval into view, maintaining the aspect ratio (e.g. by scaling by the reciprocal of the maximum of the HDPI across both axes). This results in a interface where points initially laid out in the unit interval have unchanging geometry, but the “camera view” gradually homes in on the region being selected. This approach is shown in Fig 15(a). This has the advantage of having minimal visual distortion and having familiar zooming behaviour, simulating the appearance of a camera approaching a plane along the plane normal. However, the displayed region does not completely reflect the state of the decoder and hides some context.

Alternatively, **nonlinear zooming** uses the inverse PDF $f_{i}^{-1}\left(x\right)$ directly to warp the (*x*, *y*) coordinates of each point in the unit interval. This keeps the entire geometry over all options inside a box, but gradually stretches out the regions with highest density, squashing unlikely regions towards the edges of the space. This is particularly appropriate where the density function may be multi-modal and a user may want track of the entire space to track multiple hypotheses. This approach is shown in Fig 15(b). However, it has higher visual complexity and is less familiar than straightforward linear zooming approach. For some data display types, the aspect ratio of targets must be preserved (e.g. images) and pure nonlinear zooming is not suitable.

#### 5.2.1 Implementation

**Fig 16:**
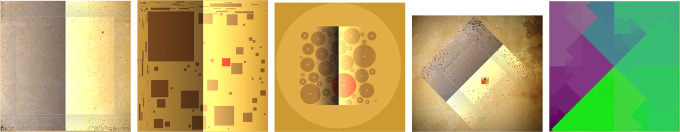
Screenshots of prototype Horstein decoder interfaces. Left-to-right: (a) Simple 2D point based non-linear zooming (b) Nonlinear zooming with rectangular area targets (c) Linear zooming with a randomised circle-packing (d) Diagonal-split interface which only requires left-right decisions (e) An interface using Jigsaw space-filling curves [111] to layout blocks of ordered targets.

We implemented a number of variants of the 2D Horstein decoder, including linear and non-linear zooming, point targets, rectangular targets, circle-packed targets, space-filling curve models, diagonal split interfaces and trisection interfaces. Images of these implementations are shown in Fig 16.

### 5.3 Packing and target identification

The use of a 2D layout expands the symbol space that can be displayed, which is essential for efficient decoding, but makes it more challenging to lay out and label items. The location of targets corresponding to symbols must be visible to users for closed-loop selection. This can either be by explicit labelling, or by implicit structure (e.g. ordering may allow interpolation). Without strong prior structure, randomly ordered items on the plane will incur significant visual search time and mental effort. We can consider the problem one of assigning each symbol *s*_*i*_ to a unique contiguous region of the unit square $X_{i}\subset R_{⊮}^{2}$, such that the area of *X*_*i*_, *A*(*X*_*i*_) ≈ *π*(*s*_*i*_), ideally such that the boundaries of *X*_*i*_ are simple.

The data that the symbols represent introduce additional constraints. For example *S* may be an ordered set of symbols (an alphabetic contacts list); it may have hierarchical grouping (filesystem paths); it may have an underlying 2D geometry (map navigation). These implicit structures reduce the dependence on explicit labelling, but there is a trade-off between preserving of structure of *S* and approximating the underlying probability distribution. In some cases, there is a natural mapping of the underlying data space to the 2D unit plane and the 2D zooming interface is trivial to apply, such as geographical map or a 2D scatterplot. Selecting a specific region is simply a matter of specifying the precision of the selection needed and running the selection process until the probability mass is sufficiently concentrated. In other cases, there is weaker structure on *S*, and a partitioning the 2D plane into areas corresponding to each *s*_*i*_ must be devised that preserves a relationship between area and *π*(*s*_*i*_).

#### 5.3.1 Space filling curves

Space filling curves, like the classical Hilbert or Peano curves, or modern compact curves like the Jigsaw curve [111] or the Balanced GP curve [112] provide a natural way to wind a 1D sequence onto a 2D unit square. This provides a straightforward mapping for ordered data into a 2D Horstein decoder. In particular, curves like the Balanced GP curve which optimise for bounding-box optimality result in subdivisions that are reasonable to select with this interface design. Space-filling curve approaches are suitable for 2D selection of ordered data types.

#### 5.3.2 Packings and tilings

In cases where ordering is not the primary organisational cue, some form of packing may be used to allocated targets to the 2D space, maintaining a probability to area relationship. Packing of rectangular or circular targets via randomised algorithms gives a straightforward way to construct an interface. This will necessarily leave gaps in the unit square, which is suboptimal from a coding point of view. This “dead space” between packed targets, however, can be reclaimed as a natural way to include a backspace control; selection of the gap area actuates backspace. Packing structures make most sense for unordered data types or for data types where a 2D layout is approximately known. For example, an image collection in a photo browser application might be laid out by some form of dimensional reduction to establish approximate 2D locations; a prior probability over images could be defined to determine target areas; and a packing algorithm used to place appropriately sized targets.

#### 5.3.3 Hierarchies

Hierarchies of symbols are easily accommodated using the strategy applied by Dasher [83] which nests sub-symbols, leading to a spatial representation of the arithmetic coding of the symbol sequence. There are three competing factors in a hierarchical 2D layout: good aspect ratio for subdivisions to maintain visibility so that labels remain clear; visual representation of the hierarchy; accurate representation of the underlying probability of each subdivision as its visual area. In 2D the treemap/squarified treemap approach [113, 114] gives a suitable algorithm for the case where the subdivisions are orthogonal to the axes. Alternative variants can subdivide the plane non-orthogonally and retain better aspect ratios [115]. This style of layout is natural for many interaction problems like navigating hierarchical file systems or hierarchical menus. Some hierarchical layout algorithms can become irregular as symbols nest deeply. Sub-optimal layouts like circle or square packings provide a simpler navigation experience, at the cost of null space.

### 5.4 Unreliable undo channels

In assistive technology contexts, it is often the case that there are multiple channels available with different reliabilities. Many of these can only be activated sporadically (e.g. because they require very significant effort to engage) and cannot be used for regular communication. These channels might take the form of muscle-activated single switches for users with limited residual motor function, electromyography to detect muscular activity [116] or a BCI **error potential** [117]. Although these channels cannot generally be used for input directly, because of their limited frequency of activation, they can usefully be used as occasional undo or backspace inputs, where they will only be required occasionally. We will term such input channels **infrequent reversal** channels, and the only symbol they can communicate is an impulse which is interpreted to undo a previous action.

Perdikis et al. [5], for example demonstrate a hybrid BCI text entry application which uses motor imagery for text input, but with an undo command activated by EMG. In a BCI context, the error potential [117–119], evoked when a subject observes that they have committed an error, is a very natural signal to trigger undo. However, the potential is only evoked if errors are relatively rare. It is not feasible to use the error potential to correct mistakes when they account for more than 10% or so of the decisions executed. Similarly, physiological changes in grip can be detected in pointing tasks in mobile devices when occasional errors occur [120], which can provide an implicit infrequent reversal channel.

There are two problems with using these infrequent reversal channels: probability of error must be limited; and the reversal channel itself is often uncertain. The first problem is easily solved as the probability of decoded error *e*_*k*_ can be precisely controlled using the Horstein decoder described above, and any arbitrary error rate can be achieved. There is also a simple solution to the problem of uncertainty in the reversal channel. Each reversal command carries a certain information value, which depends on the certainty with which it is issued and the domain to which it is expected to be applied. In a text entry example, the domain of the reversal command might be a single binary decision, a single character, a word or an entire sentence. In the Horstein scheme, an undo can be applied *within* the domain of one symbol of *k* bits. If we know the information content of the reversal channel in advance, we can apply it as follows:

- Store the CDF *F*_*i*_(*x*) at each step *i*, along with its entropy *H*_*i*_(*x*);
- If a reversal is received with information content *H*(*r*), we go back to the most recent step *j* where *H*_*j*_(*x*)≤*H*_*i*_(*x*) − *H*(*r*).

In other words, we undo as close to *H*(*r*) bits of input as possible. This could be fractional, for example undoing the last 1.7 bits of input. We can, if required, *partially* undo a single input decision, with a “partial reverse Horstein step”, rescaling both sides of the last partition of the CDF to bring it some factor closer to uniform. Lenman and Roberts discuss the importance of having multiple layers of granularity in undo [38] in human interfaces; this form of decoding allows for *continuous* undo with arbitrary granularity.

### 5.5 Non-stationary noise and diffused decoders

The Horstein decoding process assumes that errors are iid. distributed. Human input channels such are often non-stationary, as cognitive factors such as stress or exhaustion, or external physical factors in sensor configuration such as impedance changes due to electrode drying cause error rates to vary over time. One way to mitigate such effects and reduce autocorrelation in errors is to re-distribute the errors so that they are closer to iid using *n* independent decoders. Elicited input is then randomly diffused among them by interleaving inputs from the user for different subtasks.

For example, in a text entry system a sequence of letters in a word could be laid out as blanks (as in a game of Hangman), and the system randomly alternate between letters to “work on”. With each letter having its own independent decoder, bursts of errors would be diffused among the decoders, bringing the error seen by each decoder closer to an iid source and so mitigating the effect on the decoding. Doing so necessarily adds some complexity, in terms of the mental model the user must have of how their next input will affect the whole sequence, but could increase robustness. This type of diffusion can mitigate relatively short-term correlated variations in signal quality, while longer term non-independence requires online adaptation.

## 6 Monte Carlo simulation

Evaluating the performance of low-capacity interfaces by running live tests is expensive, so prior to evaluating a system with humans-in-the-loop we developed Monte Carlo numerical simulations to establish predicted performance levels. This follows the thinking of [121] and [122] who illustrated how simulator-based models could be used to iterate effective interface designs for assistive technology contexts. We present results which characterise the performance of the Horstein decoder as a function *k*, *β*, bias *b*, non-stationarity and channel mismatch. This simulator always makes perfect choices, but inputs are passed through a channel emulator which randomly introduces Bernoulli noise.

This simulation model does not account for the memory or cognitive constraints of a human controller. To investigate this *cognitive* impact of our interface, we followed this with an experimental trial with human users. This used an input device simulator which takes reliable keyboard input and injects noise to simulate different levels of signal corruption, focusing on noise levels which are at the extremes of usability for standard techniques. The human-in-the-loop addresses the question of usability of the Horstein decoder approach and validates the predictions of the Monte Carlo simulations.

### 6.1 Simulator

We constructed an offline Monte Carlo simulator in Python to evaluate the performance of the Horstein decoder with various parameter settings. We explore two cases: the performance of the Horstein decoder (as described in Algorithm 1) for various parameterisations when the channel statistics are assumed to be perfectly known and the simulator makes perfect choices; and the properties of the decoder when these assumption are violated. This includes the effect of mismatched channel statistics, where the decoder is configured for channel statistics that do not match the real bit flip probabilities; where the noise is not memoryless and there is correlation in errors over time; and where the assumption that the user has a single known target is violated.

#### 6.1.1 Concatenating with the backspace code

A real interface would apply a backspace correction to the output of the Horstein decoder, concatenating these two error correcting codes. This will reduce the uncorrected error rate to zero at a cost given by Eq 3, where *e*(*k*, *f*_0_, *f*_1_) is the uncorrected error rate of the Horstein decoder. Each set of simulation results presented shows three plots: input bits/*uncorrected* output bit *R*; the uncorrected error rate of *k* bit symbols *e*_*k*_; the predicted input bits/error free output bit *R*′ using the backspace decoder approximation of Equation 4.1.2.

### 6.2 Perfectly known channel statistics

For each configuration, the simulator executed *N* = 10000 identically parameterised random simulations. The experiments varied word lengths *k*, true simulated error rates *f*_0_ and *f*_1_, configured error rates $f_{0}^{{}^{\prime }}$ and $f_{1}^{{}^{\prime }}$ and margins *β*. The key metrics are the number of input bits the decoder must consume for each output bit produced *R*(*k*, *f*_0_, *f*_1_), and the fraction of uncorrected errors *e*_*k*_ that remain; that is the fraction of *k*-bit output symbols which are incorrectly decoded. As we are operating under perfect feedback conditions, we assume that any practical decoder interface would be concatenated with the backspace/undo decoder to make any uncorrected errors recoverable, but there is a significant penalty attached to increasing uncorrected errors and we would hope to approach small error probability with *e*_*k*_ < *ϵ* for some small *ϵ* (e.g. the 4% rate described in Section 2.1).

#### 6.2.1 Effect of *β* and *k*

**Fig 17:**
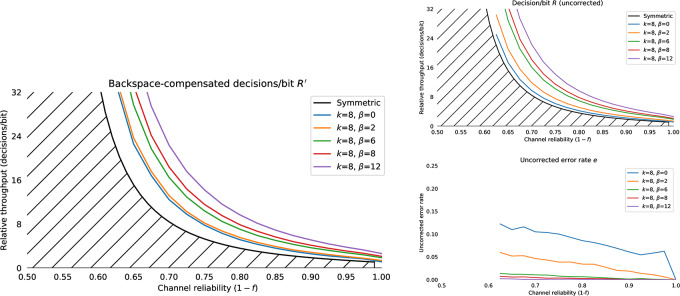
Decoder performance for varying confirmation *β*. *k* = 8 in all trials. (Left) input bits per output bit *R*, (Centre) uncorrected *k* bit symbol errors *e*_*k*_ (Right) backspace corrected rate R′(*k*; *f*). As *β* increases *e*_*k*_ → 0.

**Fig 18:**
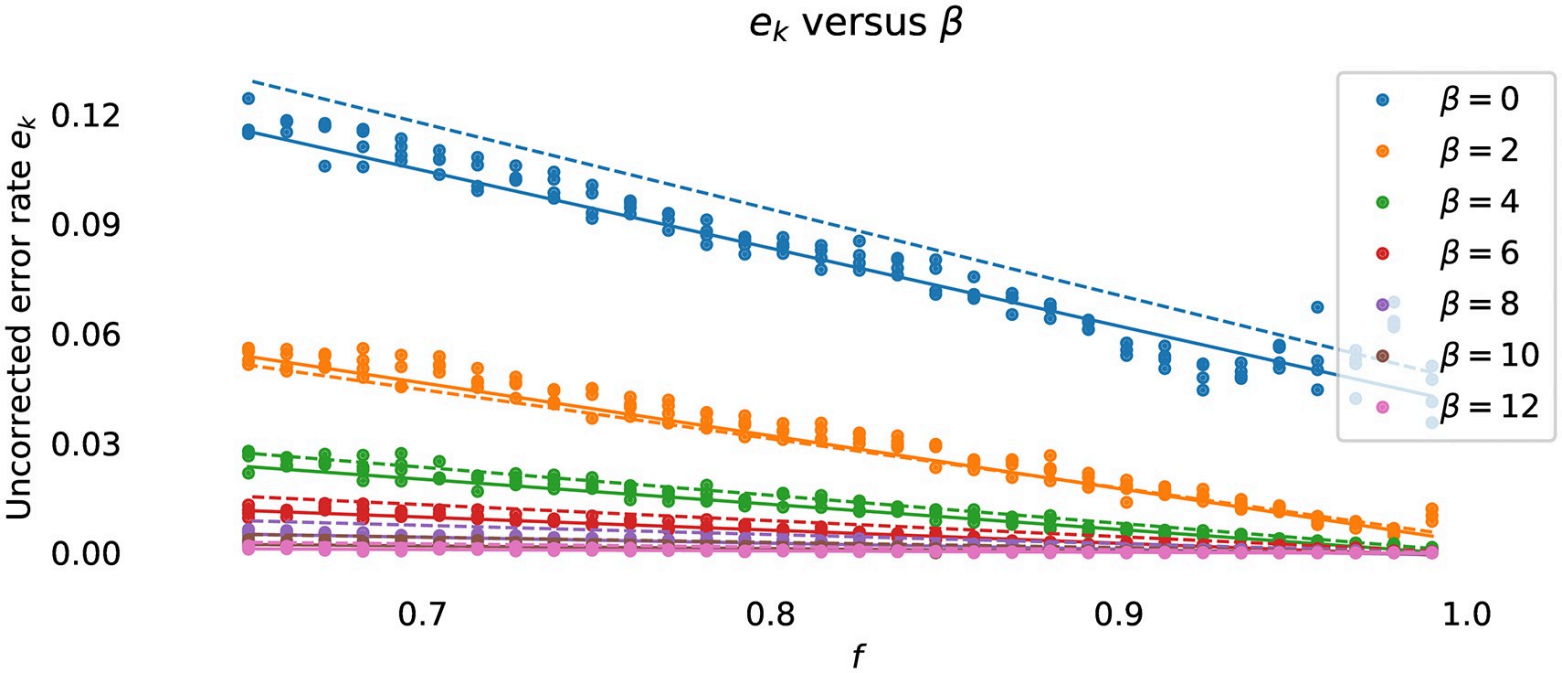
Relation of *β*, *f* and *e*_*k*_. Simulated uncorrected error rates ek from N = 10000 repetitions, for *k* ∈ 4, 6, 8, 10 and various different *β*. There is a very strong linear relation between the uncorrected error rate and *β*, which does not depend on *k*. The linear fit is shown as a dashed line. The gradient and offset of the line reduce exponentially with increasing *β*. The dashed line shows the approximated error rate *e*_*k*_(*f*) using Eq 11.

**Fig 19:**
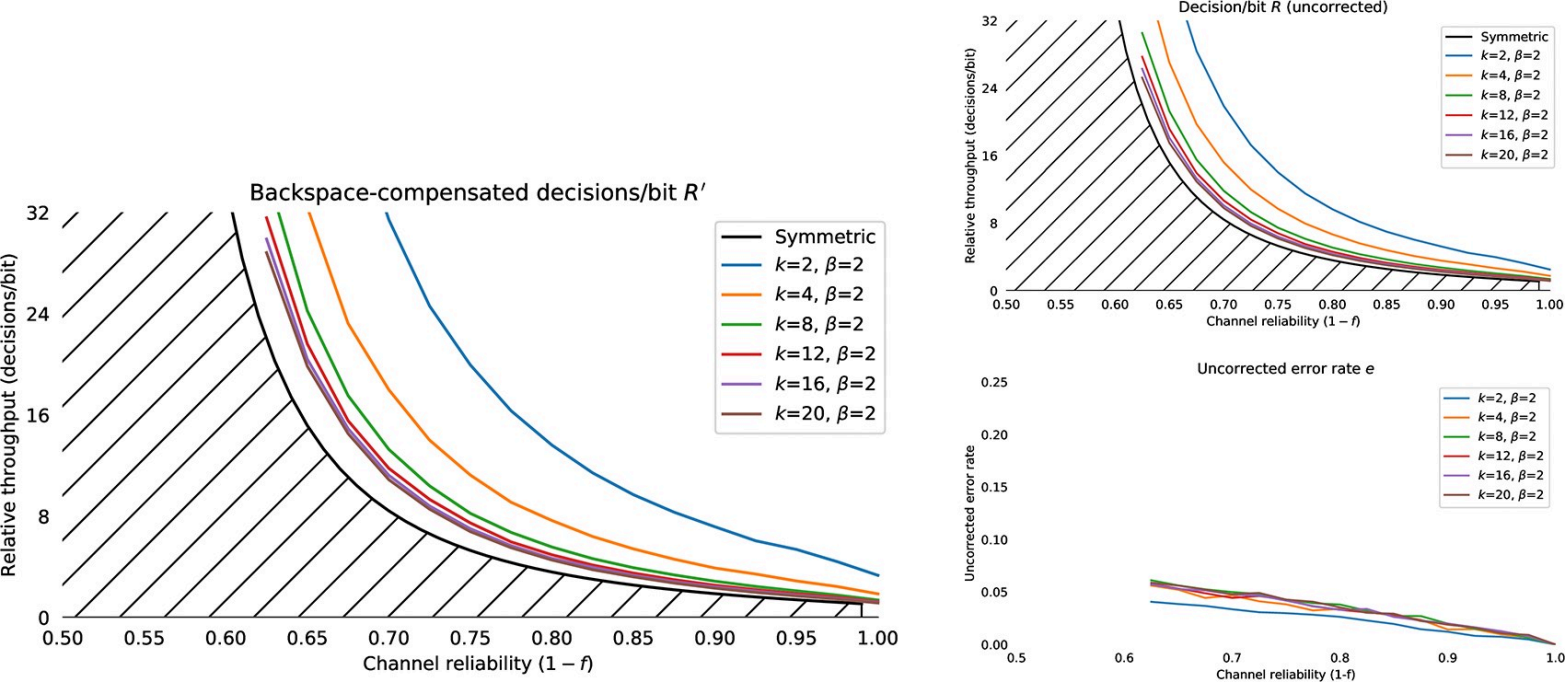
Decoder performance for varying symbol size *k*. *β* = 2. Larger *k* has improved capacity.

As *k* increases, the performance of the decoder approaches the Shannon bound (Fig 19). Shorter codes have poorer performance, as expected. As the confirmation factor *β* increases the probability of uncorrected errors decreases, at a corresponding increase in the number bits per correct symbol (Fig 17). Fig 18 shows that, for a fixed *β*, there is a very strong linear relationship between *f* and the uncorrected error level *e*_*k*_ that does *not* depend on *k*. The gradient and offset of this line reducing approximately exponentially with increasing *β*. An empirical log-linear fit to the error rate for a given bit flip probability *e*_*k*_(*f*) gives the approximation (shown in dashed lines in Fig 17):

$$e_{k}\left(f\right)=e^{-0.28(\beta +5.17)}f+e^{-1.19(\beta +2.57)}.$$ (11)

This simple closed-form approximation for *e*_*k*_(*f*) means that a desired residual error level can easily be optimised for when implementing a decoder for an input device; for example, targeting the 4% error rate discussed in Section 2.1.

The total number of bits for one correct entry with a perfectly matched decoder *j* is:

$$R\left(k,f_{0},f_{1}\right)=(\frac{k}{k+\beta })\frac{m}{{\bar{c}}_{(}f_{0},f_{1})},$$ (12)

for some constant *m*. The probability of uncorrected error depends directly on *β* (see Fig 19), so larger *k* is more efficient both because of a better approximation to the Shannon bound and because of a reduced influence of *β* on the total overhead. For larger *k* (e.g. *k* = 16), the Horstein code approaches the Shannon bound very closely. Increasing *β* > 0 typically degrades performance when combined with the backspace decoder, because it is more efficient to a small number of residual correct errors via backspace than to eliminate all errors in the Horstein stage; however, this becomes less straightforward when the channel statistics are imprecisely known. In some cases, it is important that symbols be selected accurately the first time (e.g. if they have real-world consequences that cannot be corrected after the fact, or to minimise user interface complexity). In this case, increasing *β* gives a way of reducing the error level to any arbitrary level without requiring a separate undo stage. Additionally, it should be noted that *β* can be fractional, allowing any level of confirmation to be achieved.

#### 6.2.2 Effect of bias *f*_*δ*_

**Fig 20:**
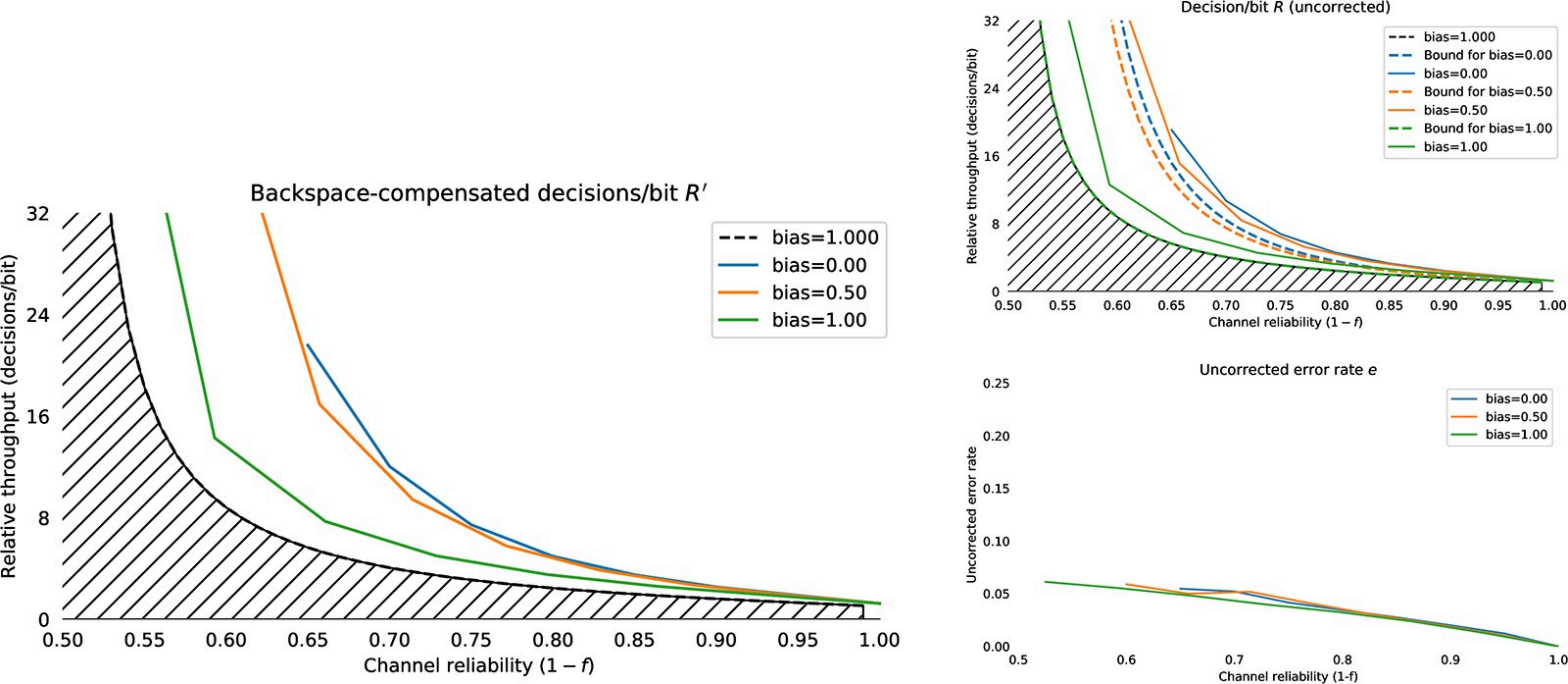
Decoder performance for varying relative bias *b*. *k* = 12, *β* = 2, *f*_*δ*_ = bf where *f*_0_ = *f* + *bf*, *f*_1_ = *f* − *bf*. Dashed lines show the theoretical bound for the given bias, and the solid lines show the mean simulated results from the Horstein decoder. The decoder approaches the bound for any level of bias.

The Horstein decoder is efficient with biased channels and can take advantage of known biases, as shown from the simulation results of Fig 20. The bias of a channel has no strong effect on the uncorrected error rate.

### 6.3 Mismatched statistics *f*′ ≠ *f*

**Fig 21:**
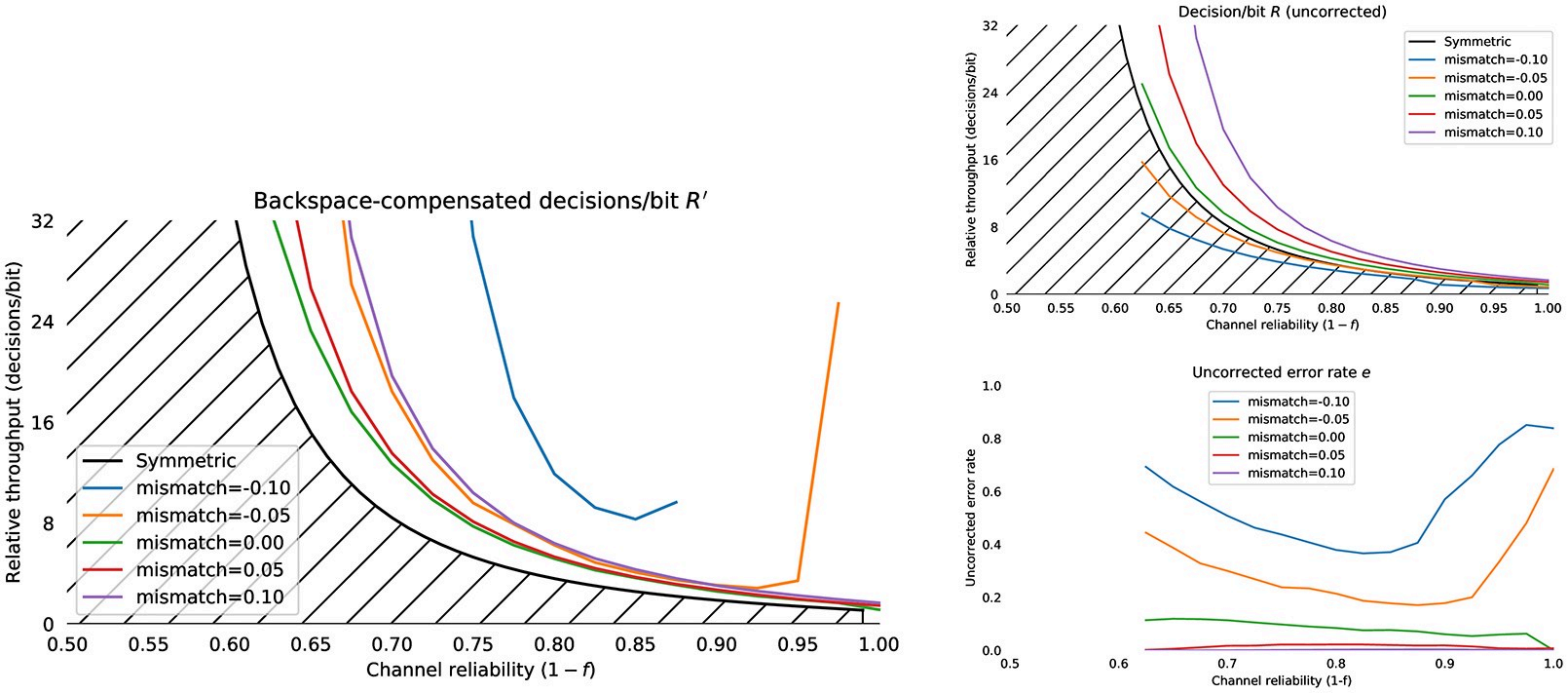
Decoder performance for mismatch between decoder and simulated statistics. *f*′ = *f* + *f*_*h*_, *k* = 8 and *β* = 0. When *f*_*h*_ < 0, the decoder is optimistic and uncorrected error rates rapidly rise. When *f*_*h*_ > 0, the decoder is pessimistic and induces a penalty to the rate while decreasing uncorrected errors.

**Fig 22:**
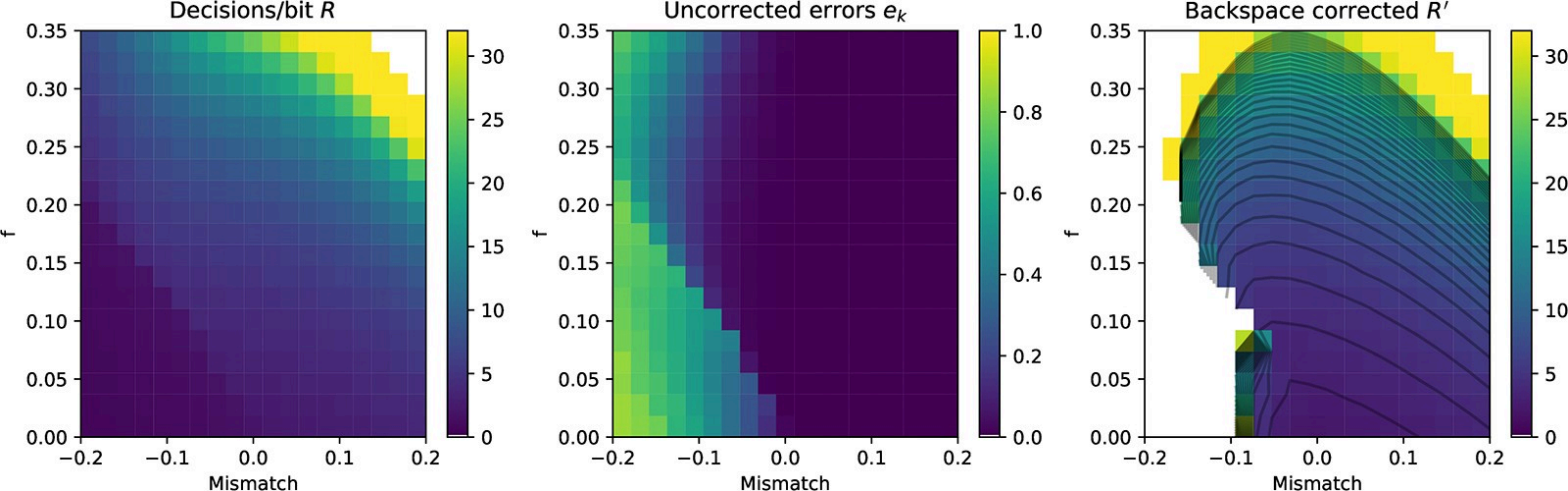
Decoder performance with mismatched statistics. Decoder performance is shown for *k* = 8 and *β* = 8, as a function of *f*_*h*_ and *f*. There is a complex trade off between capacity and the pessimism/optimism of the decoder. The mean rate (left) uncorrected error rate (centre) and backspace-corrected rate (right) are shown. White spaces indicate regions where throughput is zero due to error cascades. Each contour line indicates one additional input bit per error-free output bit.

Optimal coding for a channel requires knowledge of the reliability of that channel. An excessively robust code wastes capacity just as a insufficiently tolerant code introduces errors that must be corrected. Unfortunately, we cannot in general know the reliability of the channel precisely, and in BCI, where noise properties tend to the very non-stationary, this is a particular issue. Thus when considering the performance of our interface, we must account for the uncertainty of our estimate of the channel reliability. We performed simulations with the Horstein code which controlled both the true error probability *f* and the error probability used by the decoder *f*′, to evaluate how mismatch between true and expected error rates affects the performance of the algorithm. The results are summarised in Fig 21 and shown for a fine-grained grid of parameterisations in Fig 22. It is clear that the performance is best when *f*′ = *f*, as expected, but that the behaviour is highly asymmetric. If *f*′ < *f* there is a cliff-edge drop off in performance, reducing to nearly zero effective capacity due to rapidly increasing uncorrected error rates for even small deviations; for *f*′ > *f* there is a more gentle loss in performance where there is a gradual increase in the number of inputs required to terminate the decision for one symbol.

#### 6.3.1 Bursty channels and non-stationarity

The Horstein decoder (in the binary case) assumes corruption by memoryless iid Bernoulli noise. However, many real assistive technology channels do not have independent white noise distributions. There are often strong slowly-varying time varying components to the noise introduced, for example from classifier drift in BCI [123], electrode drying in EMG [124] or illumination changes on vision-based systems.

**Fig 23:**
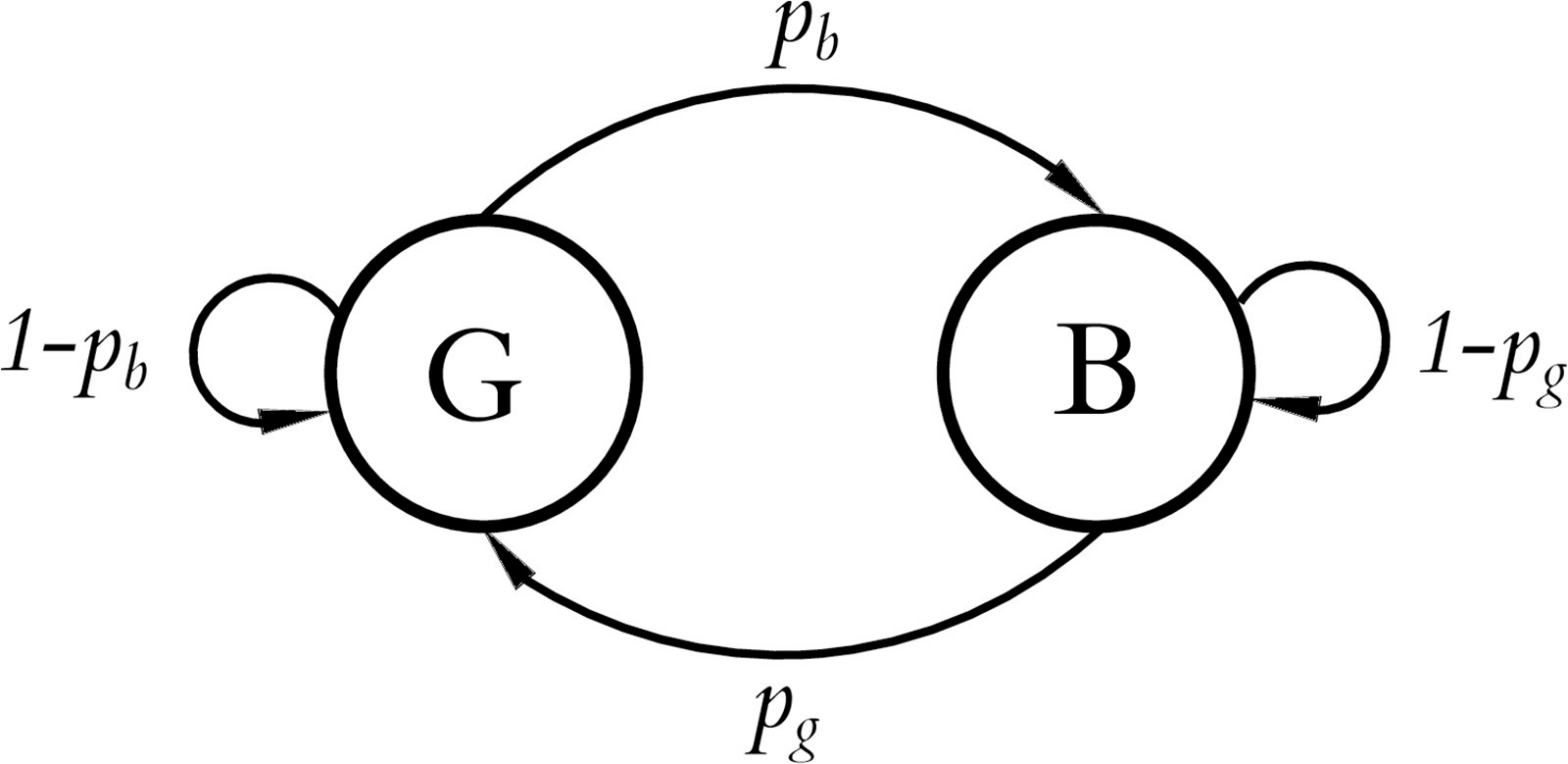
The Gilbert-Elliot Markov chain for a bursty channel. The Markov chain randomly transitions between a good state *G* and bad state *B*.

A simple but versatile model of non-iid noise in a binary channel is the **Gilbert-Elliot** bursty channel model [125, 126], which is widely used in modelling bursty packet loss on networking systems (e.g. packet-based network channels subject to varying congestion [127]). The two state Gilbert model is constructed around a binary Markov chain switching between a good state *G* with error probability *f*_*G*_ and a bad state *B* with error probability *f*_*B*_ (Fig 23). The Markov chain has transition matrix:

$$\begin{array}{c} T=[\begin{array}{cc} 1-p_{b} & p_{b}\\ p_{g} & 1-p_{g} \end{array}] \end{array}$$ (13)

and stationary distribution:

$$\pi_{G}=\frac{p_{g}}{p_{g}+p_{b}},\;\pi_{B}=\frac{p_{b}}{p_{g}+p_{b}}$$ (14)

We can apply this model to simulate the effect of non-stationarity on the Horstein decoder. Assuming that the good state *G* is perfect with no error *f*_*G*_ = 0, and the bad state *B* is always flipped *f*_*B*_ = 1, we can parameterise a Gilbert-Elliot model in terms of expected flip probability (average error rate) *f* and a “burstiness” *t*, *t* > 1. We can set:

$$\begin{array}{c} p_{b}=\frac{1}{t},\;p_{g}=\frac{\left(-fp_{b}\right)}{f-1} \end{array}$$ (15)

**Fig 24:**
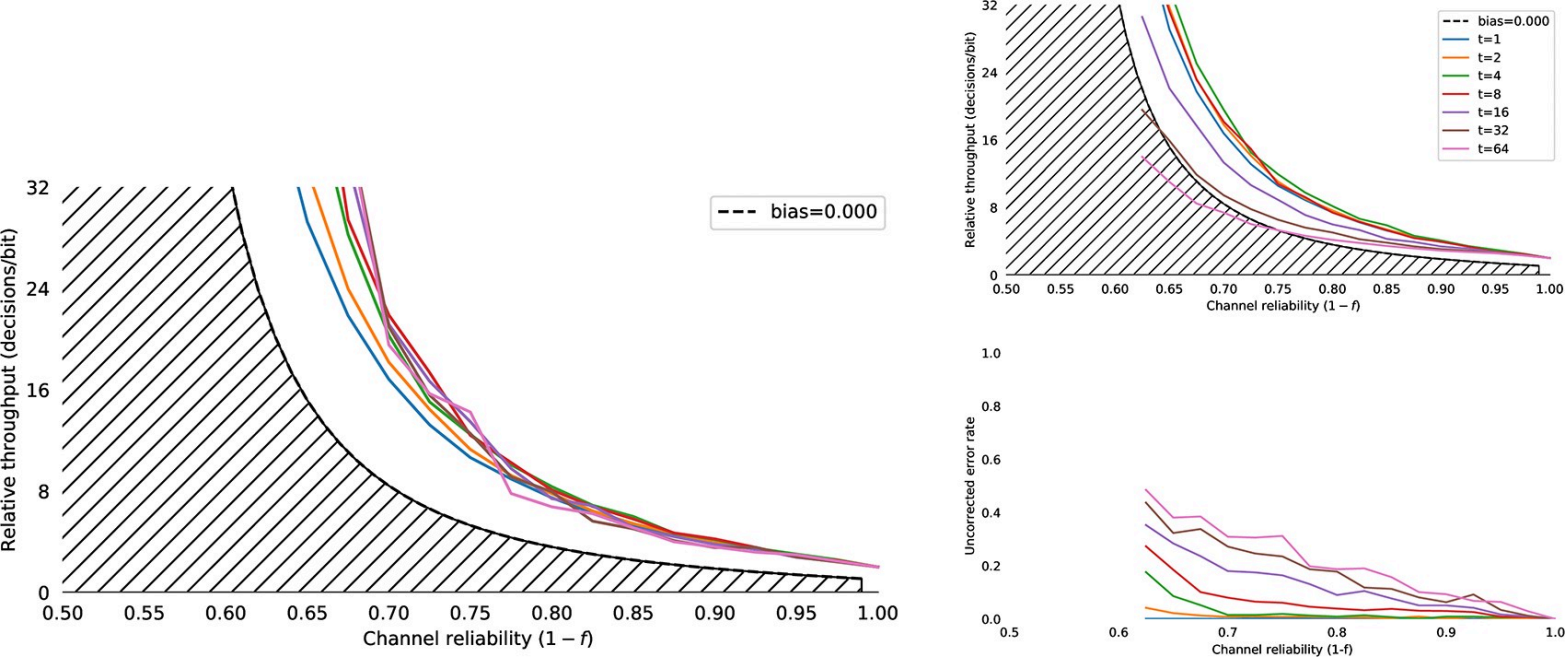
Effect of burstiness on decoding performance. The plots show the effects of varying burstiness t and average error rate f on theHorstein decoder with *k* = 8, *β* = 8 from *N* = 10000 random simulations. Increasing burstiness leads to increased uncorrected error rates but a decrease in the decisions/bit. Consequently, the backspace-corrected entry rate is largely unaffected by burstiness.

Fig 24 illustrates the effect of increasing burstiness on the Horstein decoder. As the channel becomes less iid the uncorrected error rate goes up, but the number of decisions per bit decreases because the errors become more predictable. We conclude that non-iid noise—perhaps surprisingly given that the code is only optimal for memoryless channels—does not significantly affect the performance of the Horstein decoder if we consider the backspace-corrected rate *R*′. Increasing burstiness *t* *decreases* the raw decisions/bit *R* while uncorrected error rate *e*_*k*_ increases; these effects nearly perfectly cancel out.

The Gilbert-Elliot Markov model can be generalised to good/bad states with other error probabilities and to multi-state non-stationary biased channel models where the one state may be “burstier” than the other and/or bias varies in good and bad states. We do not consider these extensions here.

#### 6.3.2 Change of heart analysis

The decoder is modelled with the assumption that the user has a specific, fixed intention for a target symbol *s* and then consistently produces inputs to drive the decoder towards that state until termination according to Equation 4.2.5. However, sometimes a user may start down a path of selecting some target *s*_*a*_, but decide they really wanted to select another target *s*_*b*_. This could be the result of an initial mistake in identifying targets, or a change in circumstances during the selection process. If the decoding process is long, completing a selection of the “wrong target”, undoing, then selecting the correct target will be frustrating. This can be seen as an extreme form of non-stationarity in error distribution.

The Horstein decoder is not designed for switches of intention, but sufficient level of tolerance *β* allows for some level of initially incorrect selection to be accommodated. We conducted a **change of heart** analysis with our simulator to quantify this performance, where we simulate users switching from intending *s*_*a*_ to *s*_*b*_ *partway through selection*, without completing the selection of *s*_*a*_.

To analyse the effect of this we run simulations where we control:

- *x*_Δ_ the **target separation**, *x*_Δ_ = |*d*(*s*_*a*_)−*d*(*s*_*b*_)|, where *d*(*s*) is the function that maps target symbol centres to the unit interval [0, 1]. Larger *x*_Δ_ indicates the decoder must make a more radical change in the probability density to select *s*_*b*_.
- λ The **switchpoint**λ, 0 ≤ λ ≤ 1 controls when the change of heart is initiated. The simulator switches targets when decoder entropy *H*(*X*)<*H*_λ_, where *H*_λ_ = −λ(*k* + *β*). For example, when λ = 0.5 the switch happens when the decoder is halfway to completion in terms of information accumulated.

**Fig 25:**
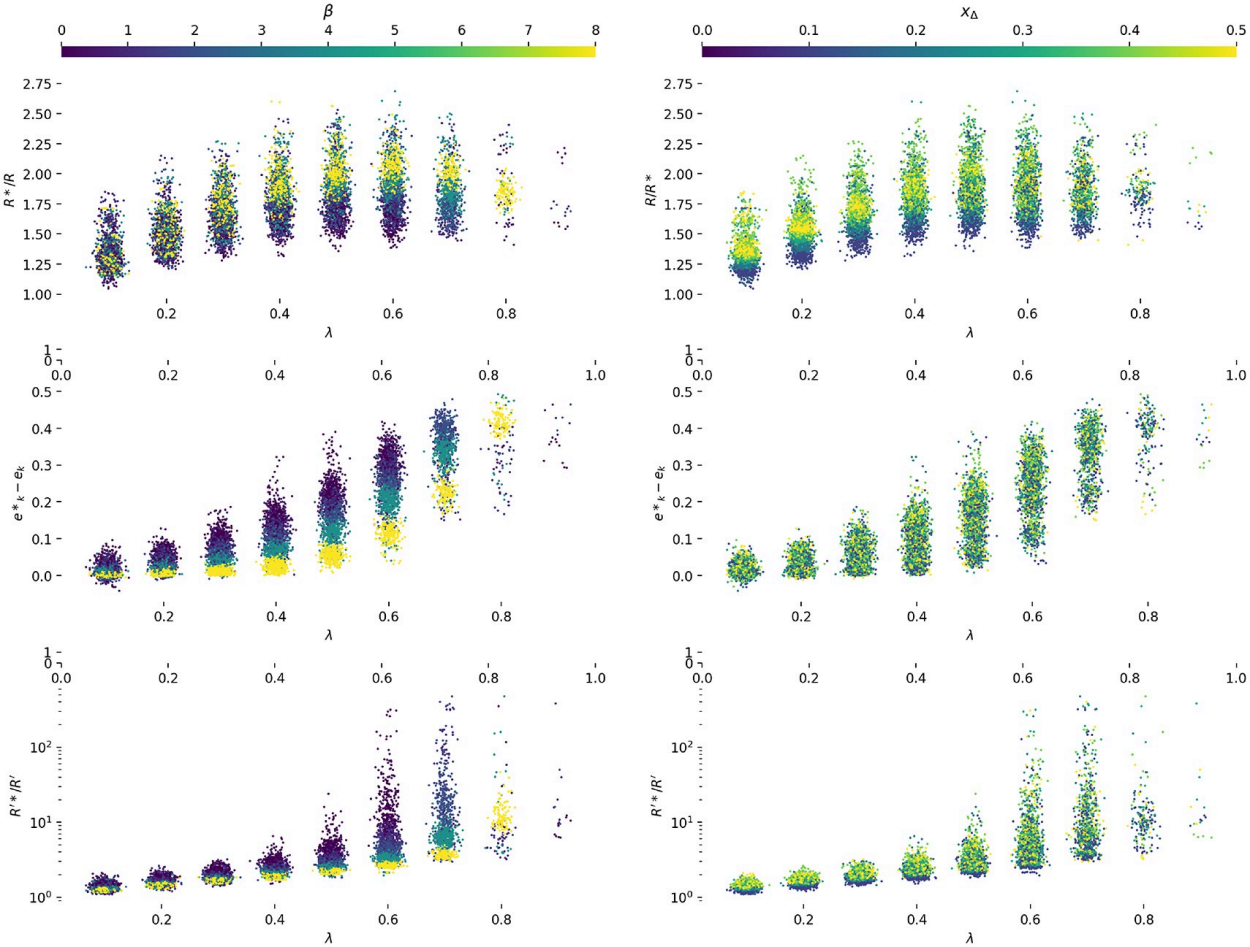
Example entropy time series with a change of heart. Change of heart occurs at λ = 0.5; *x*_Δ_ = 0.25(*H*_λ_ = −5). The decoder is configured with *k* = 8, *β* = 2; *f* = 0.15; *f*_*h*_ = 0.1, *N* = 250 trials, mean curve shown in blue. The simulation changes target when *H*_λ_ ≤ −5 to a target with separation *x*_Δ_ = 0.25. 99.2% of trials acquired the changed target sb correctly. There is a marked v shape to the curve as the decoder entropy increases after input starts to become incompatible with the original target *s*_*a*_, and then decreases as sb is approached.

**Fig 26:**
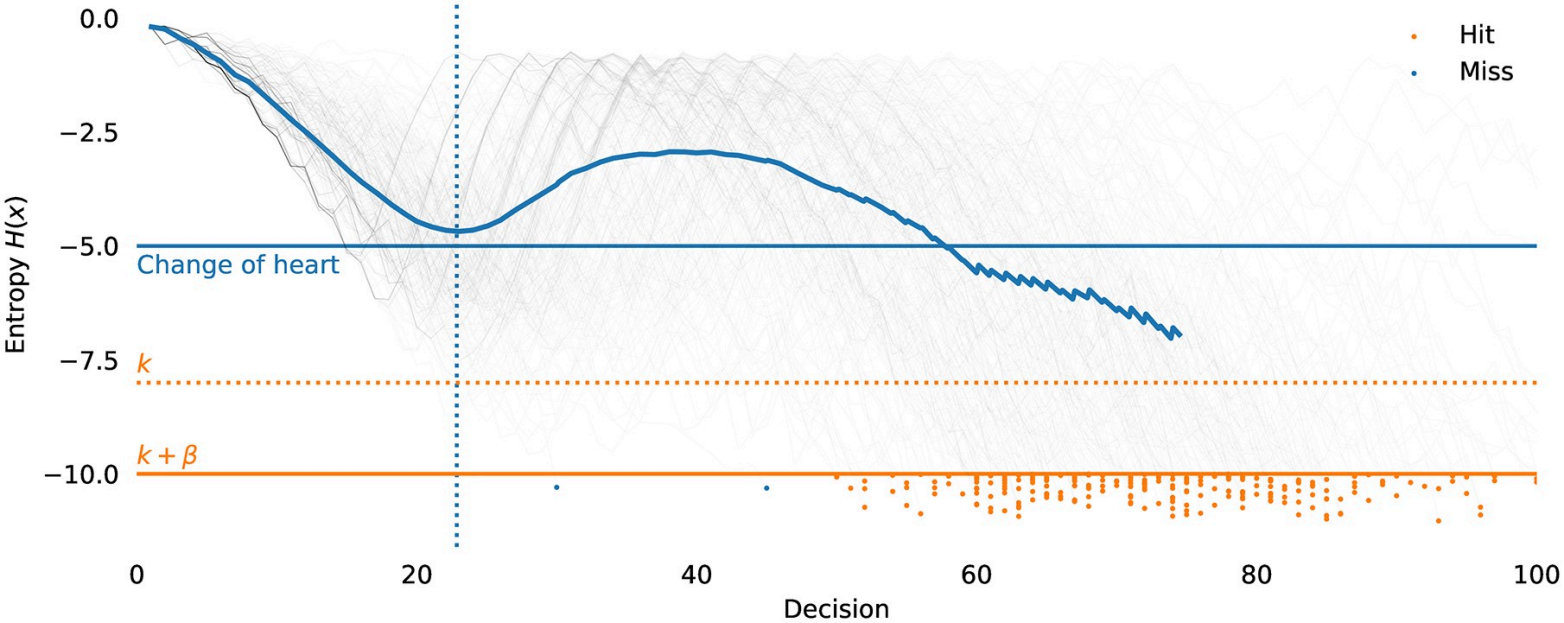
Change of heart analysis. This plot shows the effect of a sudden change of intention during selection. Coloured by *β* (left) and by *x*_*δ*_ (right). Simulations run with *k* = 8, *f* ∈ [0.0, 0.3], *β* ∈ {0, 1, 2, 4, 8}, *f*_*h*_ ∈ [0.0, 0.2], *f*′ = *f* + *f*_*h*_, *x*_Δ_ ∈ [0.0, 1.0], λ ∈ [0.0, 1.0]. Each point represents the mean of *N* = 500 trials. λ represents the proportion of the selection at which the target intention changes (in terms of decoder entropy). *x*_Δ_ is the distance between the originally intended and final targets in the unit interval. Artificial jitter added to x values to separate points.

Fig 25 illustrates the Monte Carlo simulations of the entropy decoder for a change of heart for *H*_λ_ = 0.5, *x*_Δ_ = 0.25. Following the change of heart, the decoder’s uncertainty gradually increases as inputs indicate a changed intention, then decreases as the new target becomes more certain. It is clear that the decoder can cope with changing targets, as long as *β* is sufficient. Fig 26 shows results of Monte Carlo simulations for a wide range of noise/decoder configurations. The ratio of inputs/bit from the “no change of heart” case R*/*R*, is shown, along with the absolute difference in error $e_{k}^{*}-e_{k}$ and the backspace-corrected ratio $R^{{*}^{\prime }}/R$. The bit error rate *f* and the headroom *f*_*h*_ have no noticeable effect on the ability to recover from a change of heart, but λ, *β* and *x*_Δ_ affect the recovery. In the left panel, there is a clear decrease in the uncorrected error rate *e*_*k*_ as *β* increases (brighter colours), and this is sufficiently large that even with the additional overhead increased *β* implies, the backspace-corrected ratio $R^{{*}^{\prime }}/R$ improves for larger *β*, particularly when very late corrections are made (larger λ). There is a smaller effect for *x*_Δ_ (right panel)—smaller deviations are more easily tolerated because they reduce R*/*R* but have almost no effect on the uncorrected error rate *e*_*k*_. As might be expected, small changes made early are easier to cope with, and a larger *β* can absorb more errors.

### 6.4 Adaptation

#### 6.4.1 Online adaptation for symmetric channels

**Fig 27:**
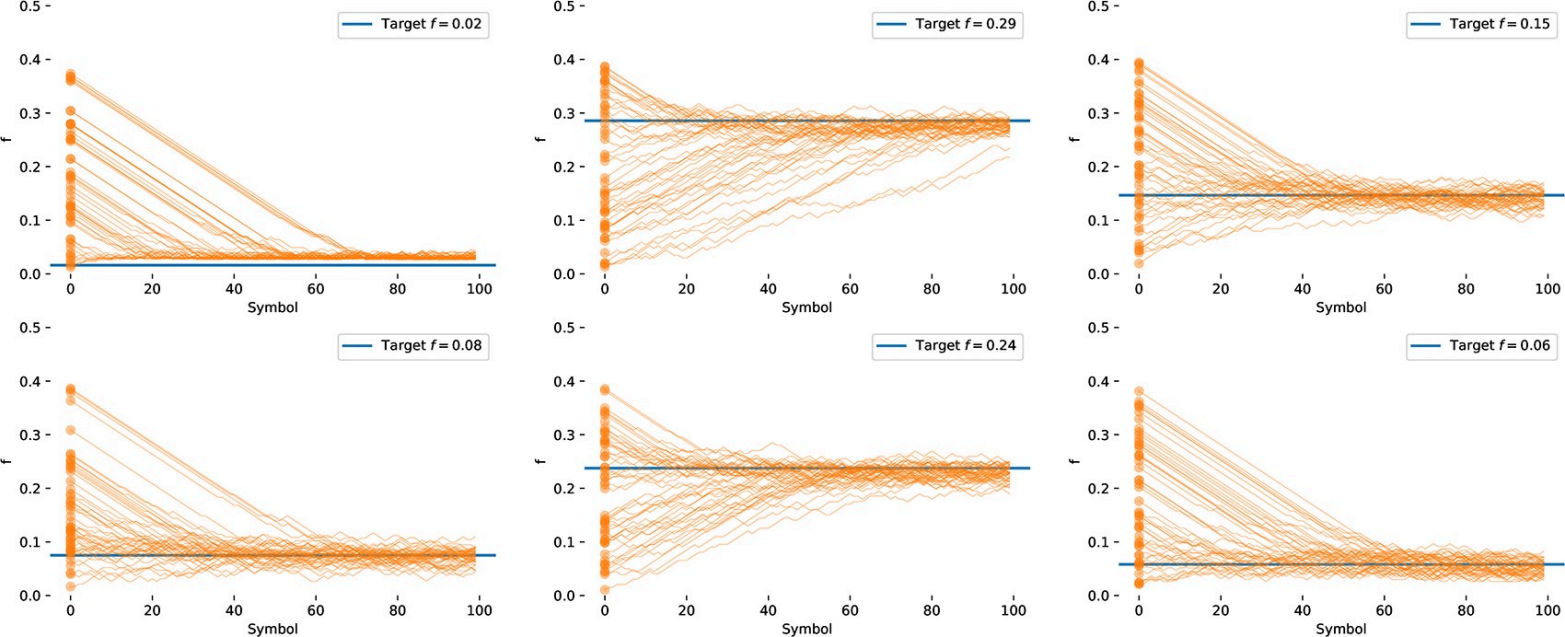
Online adaptation of the channel statistics. A decoder with *k* = 8, *β* = 8 is used for selection. In each panel, the simulated error level *f* is held fixed, and the configured expected error level *f*′ is adapted online. Each panel shows 50 replications with random initial starting values for *f*′ (shown as circles at left), showing the decoder will converge regardless of the initialisation.

Section 4.2.11 introduced an adaptive algorithm to adapt channel statistics online. We ran numerical simulations, adapting *f*′ to match an unknown (randomly selected) true error rate *f*. The simulations used *ϵ*_*n*_ = 0.01 and *δ*_*n*_ = 0.005. Fig 27 summarises the results, showing sequential runs of *k* = 8, *β* = 8 decoding with *f*_0_ = *f*_1_ = *f* with random starting *f*′ ∈ [0.01, 0.4] and fixed random target *f* in each panel. Adaptation is relatively slow, taking around 100 symbols to converge for these parameters, but this would often be sufficient for slowly-varying channels.

#### 6.4.2 Online adaptation for biased channels

**Fig 28:**
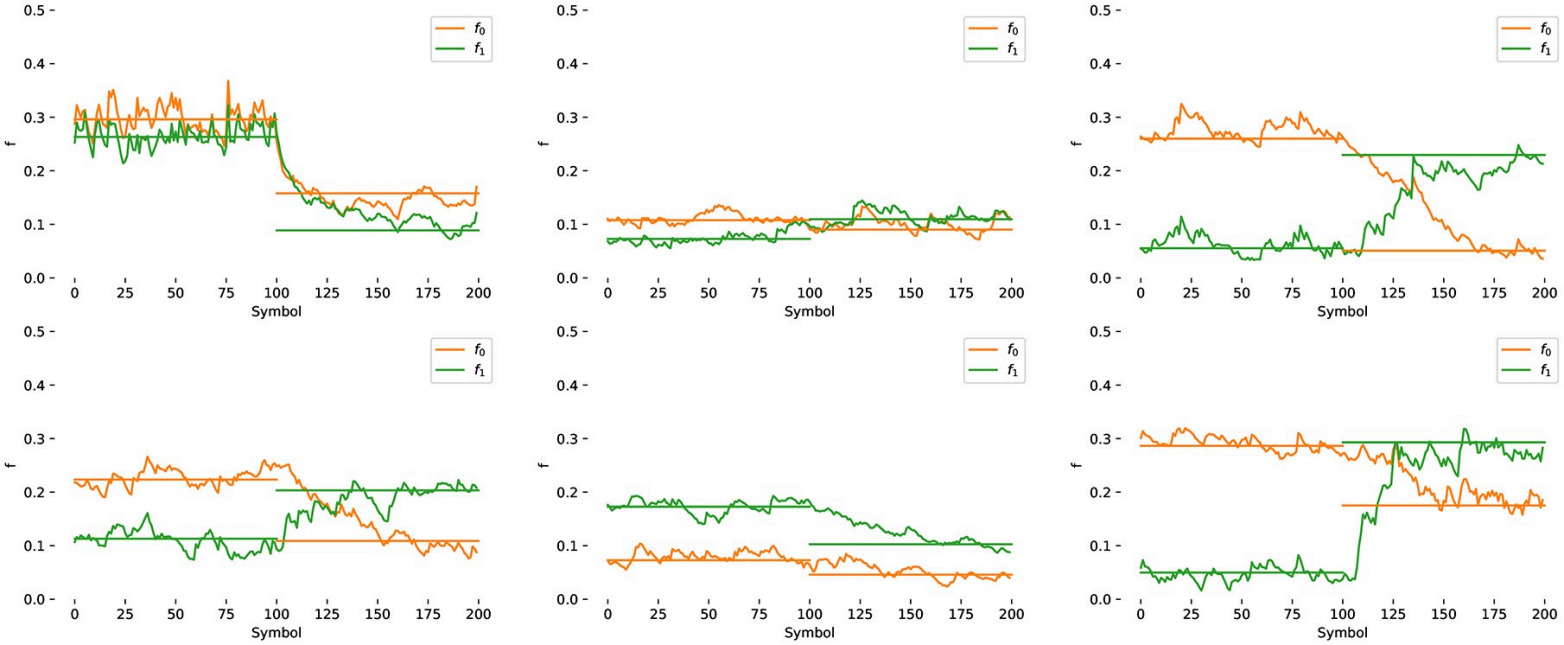
Online adaptation of the channel statistics for a biased channel. A decoder with *k* = 8, *β* = 8 is used for selection. In each panel, the decoder is initially run with ${f^{\prime }}_{0}=f_{0}$ and ${f^{\prime }}_{1}=f_{1}$ (both randomly chosen in the range [0.0, 0.35]). At symbol 100, *f*_0_ and *f*_1_ are changed to random values, and ${f^{\prime }}_{0}$ and ${f^{\prime }}_{1}$ are automatically adapted using Eq 16.

Adapting to biased channels is slightly trickier. We need to adjust the $f_{0}^{\prime }$ and $f_{1}^{\prime }$ based on the count of input bits *b*_*i*_ = 0 and *b*_*i*_ = 1, but these obviously depend on the specific target being acquired and there is not a convenient closed-form formula. However, we found a simple heuristic that can adapt to biased channels online. During selection, we record the count of *b*_*i*_ = 0, *n*_0_ and the count of *b*_*i*_ = 1, *n*_1_ received during the selection. Then, we use the numerical simulator to simulate selection of the same target—but with the current configured parameters as the simulated noise level—and count the number of 0s and 1s in this simulation, $n_{0}^{\prime },n_{1}^{\prime }$. This replicate simulation can be run multiple times and the results averaged to reduce variance. Then we update $f_{0}^{\prime }$ and $f_{1}^{\prime }$ as follows:

$$\begin{array}{c} f_{0}^{\prime }\left(j+1\right)=f_{0}^{\prime }\left(j\right)+\frac{\epsilon_{n}\left(n_{0}-n_{0}^{\prime }\right)}{k},\\ f_{1}^{\prime }\left(j+1\right)=f_{1}^{\prime }\left(j\right)+\frac{\epsilon_{n}\left(n_{1}-n_{1}^{\prime }\right)}{k}, \end{array}$$ (16)

where *ϵ*_*n*_ is a small constant. Fig 28 shows examples of online adaptation to a step change in channel statistics, using *ϵ*_*n*_ = 0.0005, with a *k* = 8, *β* = 8 decoder. This simulated change is an extreme example of channel condition variations and a real user interface would typically adapt to more slowly varying components.

### 6.5 Predicting performance in hypothetical designs

The availability of an offline numerical simulator makes it possible to thoroughly evaluate potential designs before prototype implementation and human trials. Section 7 will establish that the simulator is a viable predictor of human-in-the-loop performance with the zooming Horstein-style decoder. This section illustrates, via a set of design vignettes, how applying the Monte Carlo simulator can help explore designs and establish performance limits as part of a user-centered design process.

The expected user-sensor characteristics for a new device can be used to configure the simulator to predict a range of performance metrics, specifically uncorrected error rate, entry time and latency. This can be used to select among technologies and explore design consequences (for example, is it worth adding extra controls to a BCI-operated wheelchair?) before expensive human-in-the-loop trials. It can, for example, bound the risks in terms of task performance conditioned on of poorly known sensor characteristics like BCI classifier accuracy. While the specific task performance achieved will depend on the details of the final interface, quantitative predictions can help minimise risk in user-centred design. We illustrate this use of the simulator to predict performance in three hypothetical design scenarios:

#### 6.5.1 Scenario A: Wheelchair controls

A system is being developed for a wheelchair with four directional controls |*S*| = 4 = 2^2^; therefore *k* = 2. Errors obviously cannot be directly corrected after movement has happened, so the uncorrected error rate is taken to be 1 in 100; *e*_*k*_ < 0.01. The input is a BCI which is known to be heavily biased and expected to have *f*_0_ = 0.01 and *f*_1_ = 0.3. This estimate of accuracy is expected to be within a tolerance of *f*_*h*_ = 0.05, so a decoder is configured with $f_{0}^{\prime }=0.06$ and $f_{1}^{\prime }=0.35$. Each binary classification takes 300ms, *t*_*i*_ = 0.3.

- **Simulated performance (a)** *β* is selected via bisection to achieve *e*_*k*_ = 0.01 with *β* = 2.3. *R* = 7.64 decisions/bit. Each wheelchair command will take *T*_*k*_ = 7.64 × 2 × 0.3 = 4.61s.
- **Simulated performance (b)** If we imagine that subsequent experiments are performed which suggest that the accuracy was mis-estimated, and the real channel is *f*_0_ = 0.1, *f*_1_ = 0.4; 10% of “left” inputs are flipped and 40% of “right” inputs are flipped. With the same decoder, we get *R* = 9.48, *T*_*k*_ = 5.69s but *e*_*k*_ = 0.08 (8% error rate).

#### 6.5.2 Scenario B: Word selection

A communication support system is being built which allows users to select one word at a time from a set of *N* = 1000 common requests, so *k* = 10 ≈ log_2_(1000). Errors can be corrected by undoing the last word. The input is a eyebrow-switch which has *f* = 0.2, accurately estimated from extensive calibration. Each classification takes 500ms, *t*_*i*_ = 0.5.

- **Simulated performance (a)** With *β* = 0, *R* = 4.07, *e*_*k*_ = 0.08. The backspace-corrected rate is *R*′ = 4.99 decisions/correct bit. Each correct word will take *T*_*k*_ = 24.96s.
- **Simulated performance (b)** After a (hypothetical) trial, our imagined users indicate that they find backspace frustrating. We can use the simulator to model a reduced reliance on backspace by setting *β* = 7. This reduces the error rate to *e*_*k*_ = 0.003 at a cost of increasing *R* to 6.63. Each correct word will take *T*_*k*_ = 33.45s, and backspace will be required less than 0.3% of the time.

#### 6.5.3 Scenario C: Classifier choice

An interface to let users select a personal contact to phone from a list of |*S*| = 200 is being created. *k* = 8 ≈ log_2_(200) and we assume a tolerable error level of *e*_*k*_ = 0.01 (one misdial every 100 calls); undo is not meaningful. Three classifiers for an assistive technology sensor have been developed. In all cases, a headroom of *f*_*h*_ = 0.03 is assumed to account for mis-calibration of classifier accuracy.

- (a) Fast, unreliable, biased *f*_0_ = 0.15, *f*_1_ = 0.4, *t*_*i*_ = 0.15
- (b) Moderate, some error *f*_0_ = 0.1, *f*_1_ = 0.1, *t*_*i*_ = 0.3
- (c) Slow, reliable *f*_0_ = 0.01, *f*_1_ = 0.06, *t*_*i*_ = 1.5

**Simulated performance**

- (a) *β* = 3, *e*_*k*_ = 0.007, *R* = 10.60. *T*_*k*_ = 12.72s/dial.
- (b) *β* = 2, *e*_*k*_ = 0.007, *R* = 2.93. *T*_*k*_ = 7.03s/dial.
- (c) *β* = 1, *e*_*k*_ = 0.002, *R* = 1.58, *T*_*k*_ = 18.95s/dial.

Classifier (b) will be significantly faster for this application.

## 7 Simulation with humans-in-the-loop

We ran an experiment with human users to validate the decoder as a practical user interface for noisy binary channels. We investigated control across a range of channel reliabilities, and with both symmetric and asymmetric bit flip probabilities. The channel properties were treated as known, and the interface was configured to expect channel properties matching that of those introduced by the simulator plus some headroom to accommodate cognitive errors. To establish the usability of interface we used a simulation environment, using keyboard input and visual display, with the noisy input created by artificially randomly flipping keyboard inputs according to pre-set channel flip probabilities. These flips generated were independently distributed random samples from a Bernoulli process generated by a pseudo-random number generator. The experiment involved a participant selecting a target of a specified information capacity (12 bits, split across two six bit decoders for two spatial axes) using the diagonal split-based 2D zooming interface using binary inputs (left, right). Performance in acquiring the target was evaluated in four noisy channel conditions, along with a control noise-free condition.

### 7.1 Study design

Our study has two purposes, each of which has specific questions that are addressed:

- *Simulator validation*: Does the Monte Carlo simulator accurately predict human performance?
  - **Do users introduce errors above and beyond simulated noise?** A poorly designed interface might introduce cognitive errors in addition to expected channel noise. This would result in more frequent errors larger than that injected by the noise simulation.
  - **Are predicted entry rates close to observed entry rates?** The overall user performance in selection, in terms of decisions per bit and the backspace-corrected rate, should be close to that of the simulator.
- *Interface usability*: Can users use the interface to select targets with noisy binary inputs?
  - **Can users control the interface effectively?** The interface is intended to provide *transparent* channel coding, where users are unaware of the error correction algorithm and are simply engaged in closed-loop control of acquiring targets. We would hope to see that users control the interface:
    - **accurately**, introducing few additional errors due to confusion;
    - **quickly**, issuing inputs at a rate that indicates insignificant cognitive delay;
  - **Can users select targets under high noise conditions?** This includes noise levels that exceed those that are normally considered usable [5] with error rates *f* > 0.2.
  - **Can users select targets in the presence of strong channel bias?** Many marginal input devices are not only noisy but biased. We wish to know if users are able to communicate reliably with a biased channel, where noise may be unevenly distributed over inputs.
  - **Can we achieve a constant factor of the theoretical bounds across all channel conditions?** We wish to approach a constant factor of the Shannon bound, and performance similar to the numerical simulations of the previous section. We would hope to obtain error-free (backspace-corrected) input rates $R^{\prime }\geq \alpha \bar{R}\left(f_{0},f_{1}\right)$, for some constant *α*.
  - **Does the entropy drop smoothly?** The interface should result in a gradual drop in entropy proportional to the information content of each decision.

**Independent variables**

We manipulate the channel properties *f*_0_ and *f*_1_ (i.e. the simulated noise levels) in different conditions. The decoder is configured to decode calibrated to these noise levels, with a fixed headroom.

**Dependent variables**

We measure three primary dependent variables:

- $\hat{R}$ the measured number of decisions (keypresses) per bit communicated;
- ${\hat{e}}_{k}$ the residual uncorrected error rate;
- $\hat{T}$ the time for one decision to be made;

and the derived variables $\hat{R^{\prime }}$ the backspace-corrected rate (using the prediction from Equation 4.1.2), and ${\hat{T}}_{b}$ the time to communicate one bit.

**Hypothesis**

We hypothesise that the human-in-the-loop performance in terms of $\hat{R},\hat{e_{k}}$ will be close to that of the Monte Carlo simulator, and that *T*_*d*_ and *T*′ do not indicate any significant cognitive delays in controlling the interface.

### 7.2 Experimental setup

#### 7.2.1 Participants

N = 20 participants (10 male, 10 female) were recruited locally. The participants were healthy adult volunteers without any relevant impairments. Each participant completed the study in a single session, while seated in laboratory conditions, controlling a laptop via keyboard commands. This research was approved by the University of Glasgow College of Science and Engineering ethics board. The ethics board approval number is CSE-01125. Written informed consent was obtained from all participants. Participants were offered a £10 reward for their participation. Each experimental trial took approximately one hour.

#### 7.2.2 Visual display

**Fig 29:**
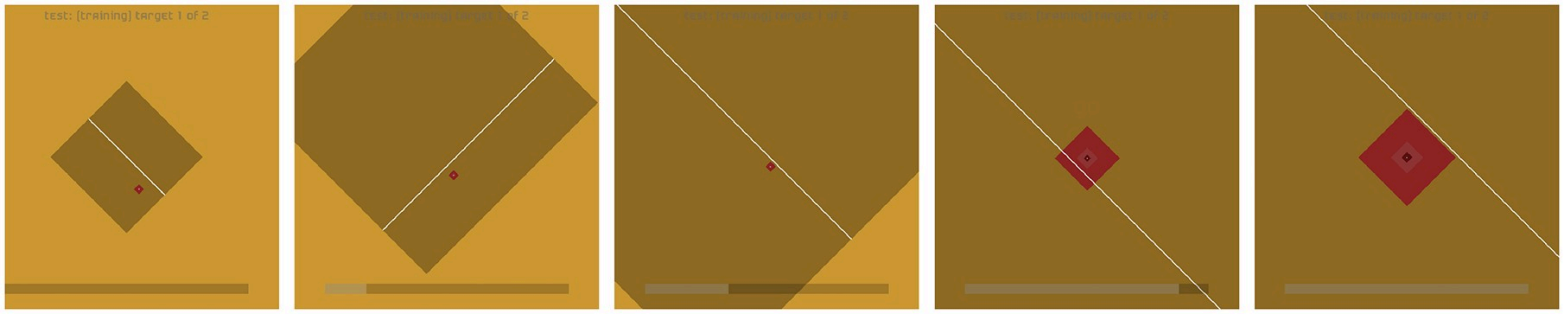
The visual display used in the experiment. A single target is shown in red, appearing at an initial random location. The target has sides of length 2^−6^. A diagonal split is used to elicit user responses and linear zooming is used. A progress bar shows the entropy drop remaining.

The visual interface appeared as the diagonal subdivision interface described in Section 5.1.2, similar to the prototype (d) in Fig 16; the exact interface layout is shown in illustrated in Fig 29. Linear zooming was used for the display, showing the smallest subsection of square such that at least of the 50% HDPI was spanned in each axis.

A single targets were represented as visually as a red square in the 2D space. We did not test the effect of searching for labelled targets. The size of the targets was fixed in terms of the information required to identify them, which corresponds to a fixed visual area in the zooming interface. The interface was configured to simulate selecting from a twelve bit alphabet of symbols (1 from 4096). A “twelve bit” target is represented as a square of sides 2^−6^ × 2^−6^ inside a unit square and requires twelve bits to reliably identify, as 2^12^ such targets will fit in a 1×1 unit square. Showing the true size of the targets in a linear zooming interface with twelve bit targets makes them very small (a few pixels across) at the initial fully zoomed out state of the interface. To make the target visible at all zoom levels, the target square was displayed as a fixed size when its true visual area would be too small to see reliably. As the display zoomed in, the target took on its true area. Fig 29 shows images of the experimental software. A progress bar showing the remaining entropy before termination was shown at the bottom of the screen.

#### 7.2.3 Trial procedure

Participants were asked to select the red square representing the target by selecting the left or right side of the visible dividing line. Participants pressed the [LEFT SHIFT] or [RIGHT SHIFT] keys to indicate a leftward or rightward movement, which would expand the space on the side of the diagonal line specified. Participants were instructed to press the key corresponding to the side of the divider the target was on; they were not given further instructions on the selection task. This process completed until the entropy of the decoder dropped by 12 bits, at which point the selection was determined to be correct if the decoder medians *m*_*xi*_, *m*_*yi*_ both lay within the target square (i.e. the correct symbol was decoded on both axes) and incorrect otherwise. There is no explicit actuation of selection in this interface; that is, there is no equivalent of a mouse click that indicates a selection happens at a particular moment. Selection is implicitly performed once the decoder is sufficiently certain. The input was user-paced (asynchronous) and participants could wait as long as desired before pressing a key, and once a key was pressed the transition happened following a 300ms delay. The transition could not be interrupted or reversed once actuated, and the screen was grayed-out during this period. An interpolated zoom was used to transition between zoom states.

#### 7.2.4 Tasks

In each condition, the participants had to select six twelve-bit targets, each target having six bits of information in the *x* and *y* axes (total of twelve bits per target), for a total of 72 bits communicated per condition. User keyboard input was randomly flipped according to the channel configuration for each condition. For example, if condition specified *f*_0_ = 0.05, *f*_1_ = 0.25, the system would actually move to the right of the dividing line 5% of the time when the user pressed [LEFT SHIFT] and move left 25% of the time when [RIGHT SHIFT] was pressed. Once the termination criteria for each target was reached, participants were invited to take a short break.

#### 7.2.5 Conditions

Before beginning the experiment, every participant performed a training condition. In the training condition, no errors were introduced and the participant was allowed to discuss what was happening with the experimenter. An onscreen label was shown to indicate where the target was, and which key to press during the training session.

Following the training session, five different conditions were presented, each with different channel properties. The full set of conditions tested are shown in Table 1. These span a range of reliabilities, including moderate error rates (C-15-15); error rates that would be very challenging for most user interfaces (C-25-25); and extremely biased inputs (C-5-45) where one control is nearly non-functional. The presentation order of the conditions following the training session was randomised for each participant to mitigate learning effects.

**Table 1:** The experimental conditions for the human-in-the-loop experiment.

Condition	*f*_0_	*f*_1_	Description
**TR-0-0**	0.0	0.0	Training condition
**C-0-0**	0.0	0.0	No error
**C-5-25**	0.05	0.25	Asymmetric, low error rate
**C-15-15**	0.15	0.15	Symmetric, low error rate
**C-25-25**	0.25	0.25	Symmetric, high error rate
**C-5-45**	0.05	0.45	Asymmetric, high error rate

#### 7.2.6 Decoder

The decoder was configured as a pair of Horstein decoders each with *k* = 6, *β* = 0 and $f_{0}^{\prime }=f_{0}+f_{h},f_{1}^{\prime }=f_{1}+f_{h}$, where *f*_0_, *f*_1_ are the simulated error levels. We set the headroom *f*_*h*_ = 0.02. For example, for C-5-45, we introduced errors to the keyboard input with *f*_0_ = 0.05 and *f*_1_ = 0.45, and configured the decoder to expect error rates of $f_{0}^{{}^{\prime }}=0.07$, $f_{1}^{{}^{\prime }}=0.47$.

#### 7.2.7 Exclusions

One participant failed to select any targets at all in the training condition, apparently due to a misunderstanding of the instructions, and was excluded from the study. The remaining 19 participants completed all tasks and their data is included in the analysis.

### 7.3 Human-in-the-loop results

#### 7.3.1 Terminology

When we report results comparing experimental results to simulations or theoretical predictions, we report the comparison of the experimentally measured decisions/bit $\hat{R}$ and uncorrected error rate $\hat{e_{k}}$ against three theoretical models. We compare against $\bar{R}\left(\hat{f_{0}},\hat{f_{1}}\right)$, the maximum possible performance at the **actual** observed channel statistics, empirically measured from the user responses, which is the most meaningful prediction; $\bar{R}\left(f_{0}^{\prime },f_{1}^{\prime }\right)$, the bound using the configured statistics (the most pessimistic model, including the headroom *f*_*h*_), and $\bar{R}\left(f_{0},f_{1}\right)$, the bound using the **simulated** statistics (i.e. the bit flip rate used to inject noise into the simulator), the most optimistic model. We use the following terms to distinguish user inputs and decoded selections: **Target**: One decoded 12 bit symbol *s*_*i*_; selecting a target is a one from 4096 choice. **Decision**: A single binary input provided by the user, corresponding to one keypress. **Bit**: One bit of the entropy used to select a target. “Decisions per bit” means the number of keypresses required to select 1/12th of a target.

### 7.4 Validation of the simulator

Table 2 shows the summary of the experimental results for each condition, including the actual measured error rates *f*_0_, *f*_1_, the number of decisions per bit measured $\hat{R}$, the uncorrected error rate $\hat{e_{k}}$, and the predicted number of decisions per bit for perfect entry using Equation 4.1.2 $\hat{R^{\prime }}$.

**Table 2:** Summary of experimental results, including observed input error rates $\hat{f_{0}},\hat{f_{1}}$, decisions/bit $\hat{R}$, uncorrected errors ${\hat{e}}_{k}$ and backspace-corrected equivalent rates ${\hat{R}}^{\prime }$.

Condition	$\hat{f_{0}}$	$\hat{f_{1}}$	Decisions $\hat{R}$	Errors ${\hat{e}}_{k}$	Backspace rate ${\hat{R}}^{\prime }$
**C-0-0**	0.01	0.01	1.52 (0.33)	0.04 (0.11)	1.61 (0.29)
**C-5-25**	0.06	0.25	4.16 (1.09)	0.16 (0.18)	2.19 (0.39)
**C-15-15**	0.14	0.16	3.98 (0.72)	0.08 (0.10)	1.77 (0.31)
**C-25-25**	0.26	0.25	8.35 (1.39)	0.11 (0.10)	1.90 (0.34)
**C-5-45**	0.05	0.45	6.64 (1.47)	0.13 (0.14)	2.03 (0.36)
**TR-0-0**	0.01	0.01	1.47 (0.26)	0.02 (0.07)	1.54 (0.27)

**Fig 30:**
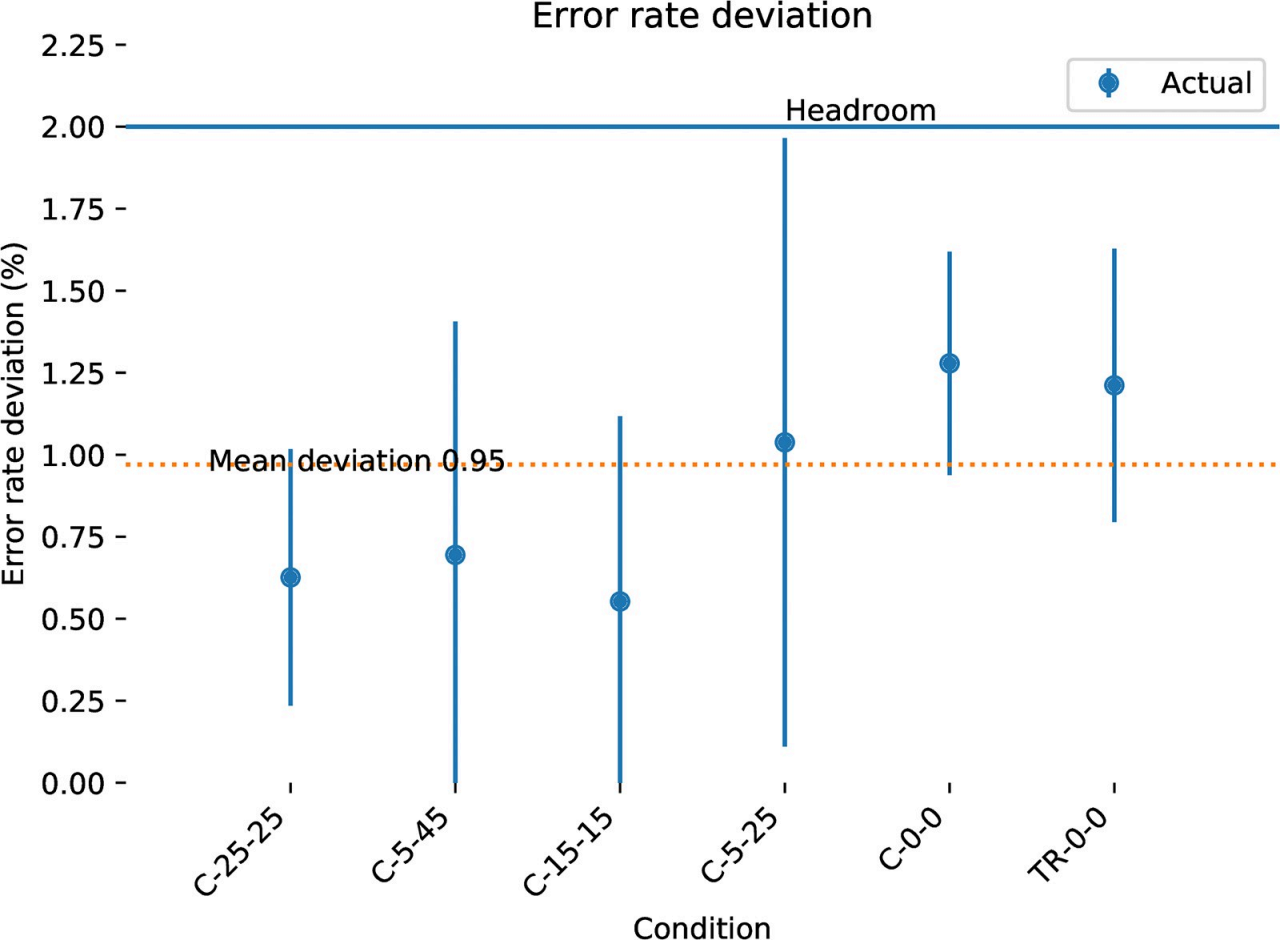
Additional input errors introduced over the simulated noise. Error bars are 95% CI. Red line indicates the headroom *f*_*h*_. Mean additional input error is 0.95%.

We verified that the actual input bit error rates observed are within the headroom, i.e. that users introduced *additional* errors at a rate less than 2% (Fig 30). An average additional error rate of ≈0.95% was observed.

Table 3 compares the human-in-the-loop experimental results to running the numerical simulator of the Horstein decoder from Section 6.1. This accurately predicts the effect of the short symbol size *k* = 6. It shows decisions (keypresses) required to communicate each (uncorrected) bit of information and uncorrected error rate across conditions, compared to a numerical simulation (*N* = 1000) trials for a *k* = 6, *β* = 0 decoder. In each case, the decoder is configured with the same values as the experimental trial. In the **actual** simulation, input errors are introduced at the same rate as empirically determined from the experiment $\hat{f_{0}},\hat{f_{1}}$; the **expected** simulation uses the induced error rate *f*_0_, *f*_1_; and the **configured** simulation introduces input errors at a rate of $f_{0}^{{}^{\prime }},f_{1}^{{}^{\prime }}$. Performance in the human trials is very similar to what would be expected from the simulated decoder running with the actual observed error rates in the input channel though the biased conditions, particularly **C-5-25**, have a higher uncorrected error rate than would be expected.

**Table 3:** Comparison of experimental results $\hat{R},{\hat{e}}_{k}$ with numerical simulations *R*_*s*_, *e*_*s*_.

Condition	$\hat{R}$	$R_{s}\left(k,\hat{f_{0}},\hat{f_{1}}\right)$	*R*_*s*_(*k*, *f*_0_, *f*_1_)	$R_{s}\left(k,f_{0}^{\prime },f_{1}^{\prime }\right)$
**C-0-0**	1.52 (0.33)	1.42	1.38	1.47
**C-5-25**	4.16 (1.09)	3.28	3.16	3.43
**C-15-15**	3.98 (0.72)	3.48	3.38	3.70
**C-25-25**	8.35 (1.39)	7.17	7.09	7.54
**C-5-45**	6.64 (1.47)	5.56	5.65	6.07
**TR-0-0**	1.47 (0.26)	1.43	1.39	1.48
	${\hat{e}}_{k}$	$e_{s}\left(k,\hat{f_{0}},\hat{f_{1}}\right)$	*e*_*s*_(*k*, *f*_0_, *f*_1_)	$e_{s}\left(k,f_{0}^{\prime },f_{1}^{\prime }\right)$
**C-0-0**	0.04 (0.11)	0.05	0.03	0.06
**C-5-25**	0.16 (0.18)	0.06	0.05	0.06
**C-15-15**	0.08 (0.10)	0.06	0.06	0.08
**C-25-25**	0.11 (0.10)	0.09	0.06	0.10
**C-5-45**	0.13 (0.14)	0.06	0.06	0.10
**TR-0-0**	0.02 (0.07)	0.04	0.03	0.06

### 7.5 Usability

We next consider the questions of usability, and whether the performance of users was compatible with effective control of an interface. Table 4 shows the mean number of targets correctly selected (all 12 bits correctly communicated) for each condition, and the mean correct bits communicated. As ${\hat{e}}_{k}$ is not zero, some residual error remains; this could have been reduced to any arbitrary level by increasing *β* at a cost of slower input.

**Table 4:** Average number of targets selected correctly by participants, and the average number of bits entered correctly, averaged across all targets in each condition. The average time to select one target is also given. Each condition has six twelve-bit targets.

Condition	Correct targets (/6)	Correct bits (/12)	Time per target (s)
**C-0-0**	5.76 (0.64)	11.64 (0.96)	93.22 (39.28)
**C-15-15**	5.53 (0.60)	11.62 (0.99)	188.68 (27.89)
**C-25-25**	5.37 (0.58)	11.46 (1.19)	402.91 (86.42)
**C-5-25**	5.05 (1.05)	11.48 (1.26)	184.33 (36.08)
**C-5-45**	5.21 (0.84)	11.44 (1.25)	289.53 (52.65)
**TR-0-0**	5.89 (0.45)	11.72 (0.92)	104.17 (24.35)

#### 7.5.1 Can users approach the Shannon bound?

**Fig 31:**
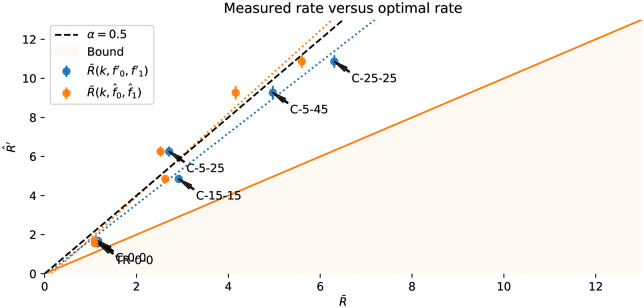
Measured performance against Shannon upper bound. $\hat{R^{\prime }}$ is plotted against $\bar{R}\left(k,f_{0},f_{1}\right)$, $\;\bar{R}\left(k,\hat{f_{0}},\hat{f_{1}}\right)$ for each condition. A line at *α* = 0:5 showing 50% of the Shannon bound is shown.

The most salient overall metric is the number of input bits required to produce one *error-free* output bit. Our simulator did not include a backspace function, but we can directly estimate the correction penalty required to get error-free output using Equation 4.1.2. This gives a directly comparable measure to the theoretical channel bounds. The key results are Table 5, which compares the backspace-corrected observed entry rates $\hat{R^{\prime }}$ against the numerical simulation using the **actual** channel statistics $R_{s}^{\prime }\left(k,f_{0},f_{1}\right)$ and the Shannon bound for the **actual** statistics $\bar{R}\left(k,\hat{f_{0}},\hat{f_{1}}\right)$. The percentage of the Shannon bound achieved is also given, showing that the interface achieves approximately 50-75% of the theoretical maximum. The backspace-corrected decision/bit rates against all three of the theoretical models are summarised in Table 6, which compares the decisions per/bit across conditions, along with the theoretical minimum from Eq 2 at each of the **actual**, **expected** and **configured** models. Fig 31 shows a regression of the observed $\hat{R}$ against the theoretical bounds for the **actual** and **configured** models. Performance is nearly linear across the full range of error rates, and is on average 54% of the theoretical upper bound for the **configured** statistics.

**Table 5:** Backspace-corrected experimental rates against simulated backspace-corrected rate and Shannon bound, and fraction of simulator performance/Shannon bound achieved.

Condition	$\hat{R^{\prime }}$	$R_{s}^{\prime }\left(k,f_{0},f_{1}\right)$	$\bar{R}\left(k,\hat{f_{0}},\hat{f_{1}}\right)$	%. sim.	% max.
**C-0-0**	1.69 (0.37)	1.65	1.09	97.7%	64.5%
**C-5-25**	6.27 (1.64)	3.71	2.46	59.2%	39.3%
**C-15-15**	4.84 (0.88)	3.81	2.56	78.6%	52.9%
**C-25-25**	10.91 (1.82)	8.23	5.53	75.4%	50.7%
**C-5-45**	9.28 (2.05)	6.48	4.09	69.8%	44.1%
**TR-0-0**	1.55 (0.27)	1.59	1.09	102.0%	70.0%

**Table 6:** Backspace-corrected rate against theoretical Shannon upper bounds.

Condition	$\hat{R^{\prime }}$	$\;\bar{R}\left(k,\hat{f_{0}},\hat{f_{1}}\right)$	$\bar{R}\left(k,f_{0},f_{1}\right)$	$\bar{R}\left(k,f_{0}^{\prime },f_{1}^{\prime }\right)$
**C-0-0**	1.69 (0.37)	1.09	1.00	1.16
**C-5-25**	6.27 (1.64)	2.46	2.36	2.71
**C-15-15**	4.84 (0.88)	2.56	2.56	2.92
**C-25-25**	10.91 (1.82)	5.53	5.30	6.31
**C-5-45**	9.28 (2.05)	4.09	4.09	4.97
**TR-0-0**	1.55 (0.27)	1.09	1.00	1.16

**Fig 32:**
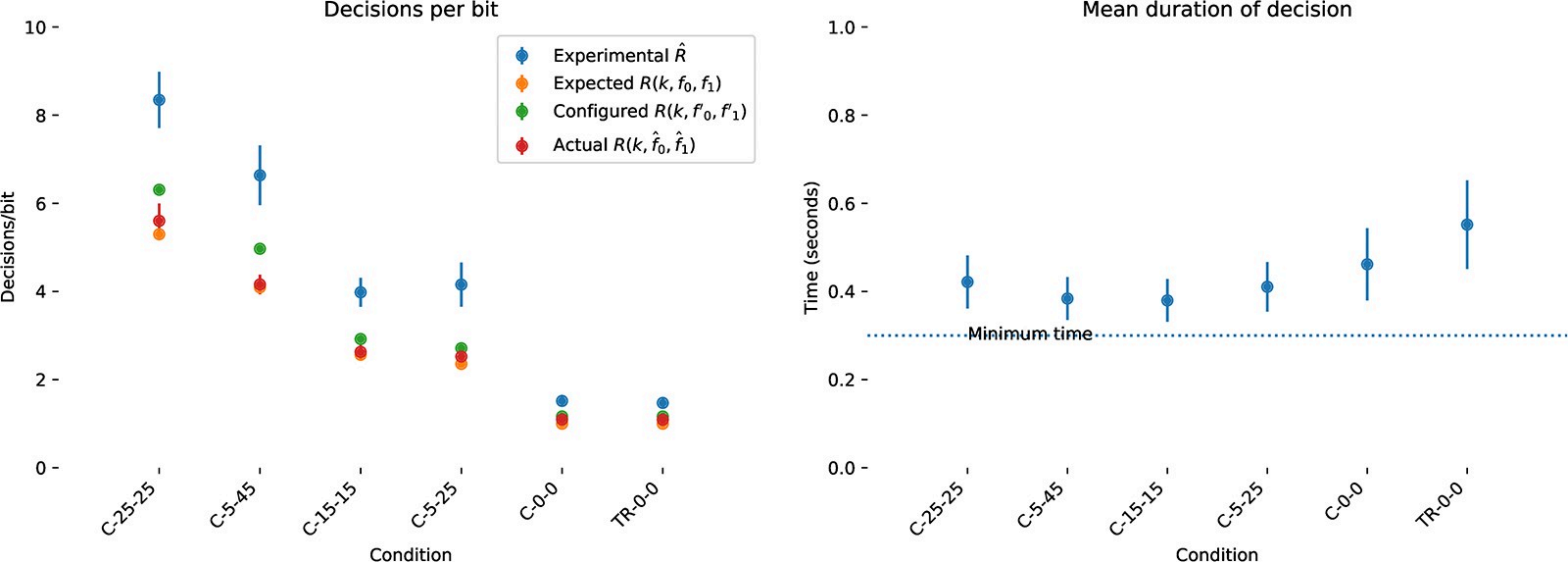
Decisions per bit and timing of decisions. (Left) The number of decisions R required for each bit (Right) The time taken for each decision $\hat{T}$, which remained approximately constant across conditions. Red line shows the minimum fixed delay.

Table 7 summarises the timing of the inputs including duration to select each 12 bit target, the number of keypresses in the whole condition (for six targets), the duration of each condition in seconds, and the average number of keypresses per second. There is no strong variation across conditions in terms of input timing. Fig 32 summarises the effort required to make each selection, including the number of binary inputs per bit successfully communicated and the mean time taken for each binary decision. Users showed little variation in generating inputs, suggesting they did not spend long pondering the correct decision to move towards the target.

**Table 7:** Timing of inputs. All numbers in seconds. $\hat{T}$ is duration of one decision (from prompt to keypress); *T*_min_ is the 300ms minimum delay enforced; ${\hat{T}}_{b}=\hat{R}\hat{T}$ is the time taken to enter one bit of information, on average.

Condition	$\hat{T}$	$\hat{T}-T_{\text{min}}$	$\frac{1}{\hat{T}}$	${\hat{T}}_{b}$
**C-0-0**	0.46 (0.26)	0.16 (0.26)	2.60 (1.01)	7.77 (3.32)
**C-15-15**	0.38 (0.11)	0.08 (0.11)	2.84 (0.77)	15.72 (2.38)
**C-25-25**	0.42 (0.13)	0.12 (0.13)	2.61 (0.81)	33.58 (7.37)
**C-5-25**	0.41 (0.13)	0.11 (0.13)	2.64 (0.73)	15.36 (3.08)
**C-5-45**	0.38 (0.11)	0.08 (0.11)	2.79 (0.76)	24.13 (4.49)
**TR-0-0**	0.55 (0.22)	0.25 (0.22)	2.12 (0.90)	8.68 (2.08)

#### 7.5.2 Illustrations of entry process

**Fig 33:**
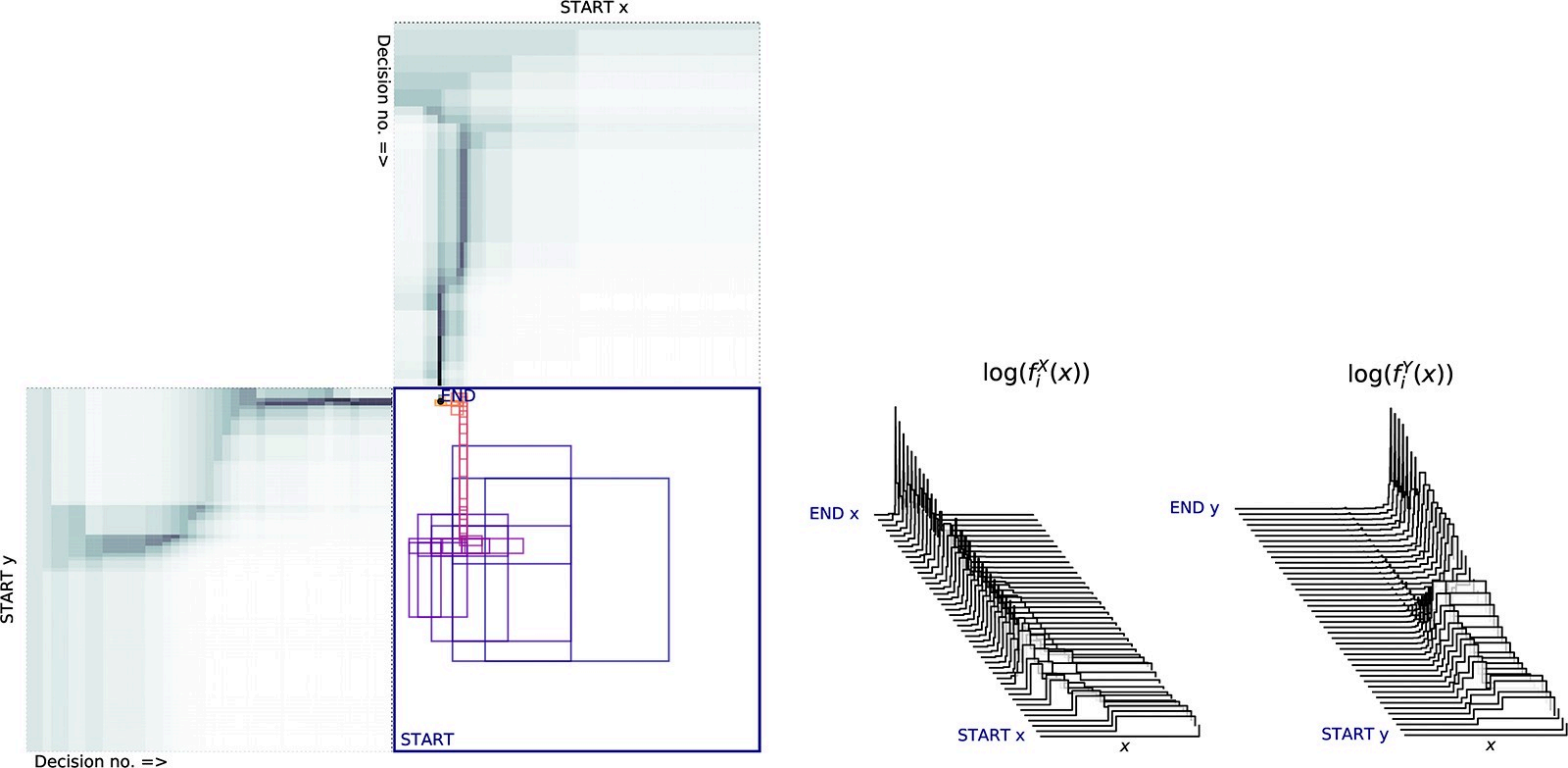
PDF evolution form a random run from C-15-15. (above) Colormap showing PDF after each input; darker indicates greater density (below) Ridge-plot of the same density sequence, where the height of the line is proportional to the log PDF; each line corresponds to a single decision. The maintenance of multiple hypotheses is visible.

**Fig 34:**
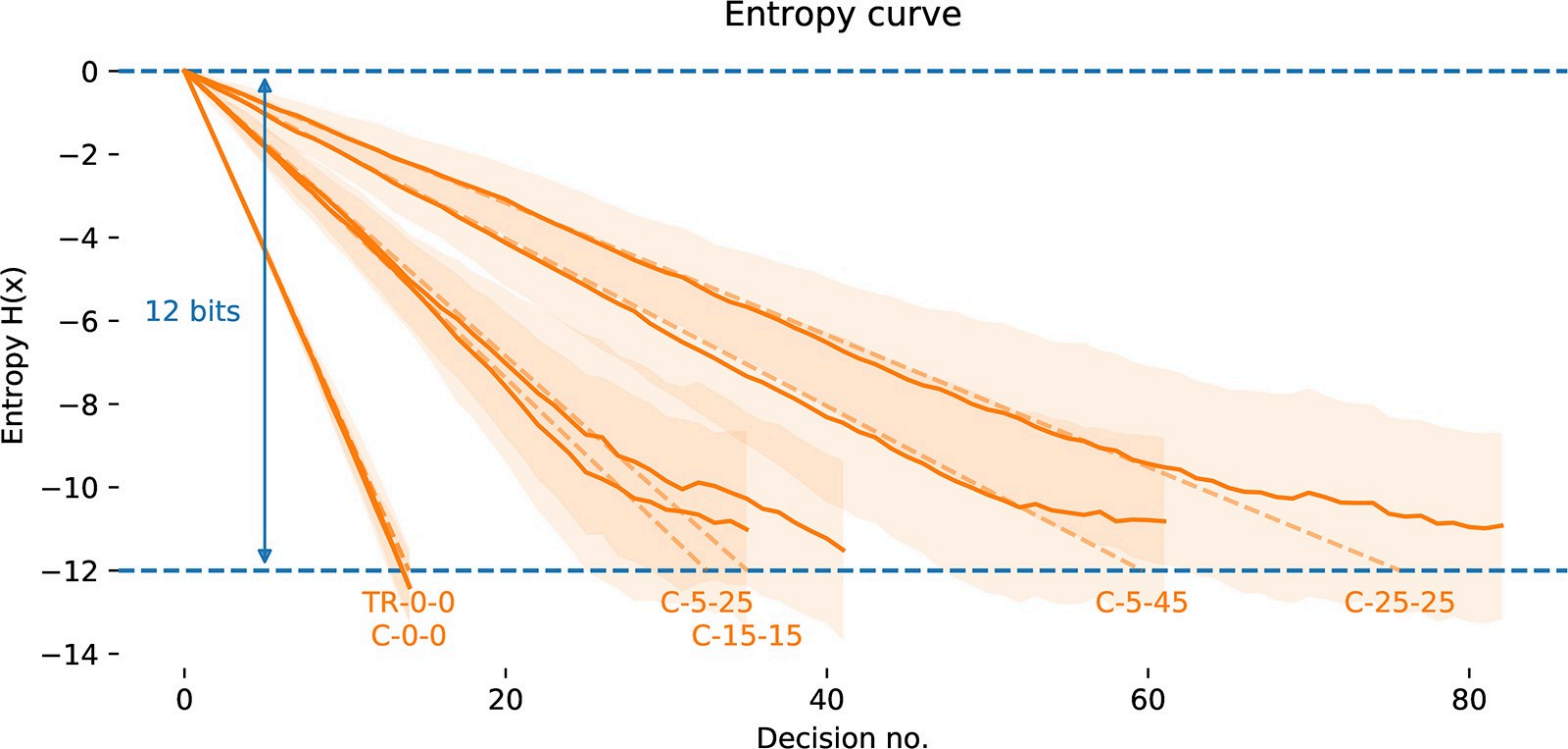
Mean entropy time series. Each plot shows the mean (solid line), one standard deviation (shaded area) and the theoretical prediction (dashed line) of the entropy of the decoder’s PDF against decision number, for each of the experimental conditions.

As a user interacts with the linear zooming interface, there is a visual expansion of the view corresponding to the concentration of probability density. Fig 33 illustrates the viewports displayed in one example trial after each keypress. The marginal probability densities *p*_*x*_(*x*), *p*_*y*_(*x*) for the *X* and *Y* axes after each keypress are shown. The gradual contraction of probability density around the target is clearly visible. Fig 34 shows how entropy of the PDFs decreases as each input is received during selection of a target, averaged across all users for each condition. As would be expected, the PDF decreases by twelve bits across the target selection process. The information rate is almost exactly the linear drop that would be predicted.

### 7.6 Discussion

- *Simulator validation*
  - **Do users introduce errors above and beyond simulated noise?** Users introduced input errors at around a rate of 0.95%, suggesting there was little confusion as to the correct action at each timestep. This is within the headroom of 2% the decoder was configured for in the human-in-the-loop experiments.
  - **Are predicted entry rates close to observed entry rates?** As Table 3 indicates, user performance is between 59%-99% of the predicted simulation performance, with lowest results in the most biased conditions (**C-5-25** 59.2% and **C-5-45** 69.8%). Other conditions acheive greater than 75% of the simulated predictions. The numerical simulation is a good but not perfect predictor of performance.
- *Interface usability*
  - **Can users control the interface effectively?** All 19 participants were able to control the interface and select targets efficiently. Table 5 indicates that users were able to select the vast majority of targets correctly in all conditions, and this is well predicted by the expected residual error *e*_*k*_ estimates from the simulator in Table 3. Overall performance was close to what would be expected from the numerical simulations of Section 6.1.
    - **Accuracy** Error rates ${\hat{e}}_{k}$ are comparable to Monte Carlo simulation, though above slightly raised in the biased conditions. This may be caused by “key-leaning” when frustrated users repeatedly hit the “bad” input without waiting for feedback.
    - **Speed** Time per decision was close to the maximum possible rate resulting from the 300ms transition time and varied little from condition to condition (Fig 32).
  - **Can users select targets under high noise conditions?** Users were able to successfully select targets in channels with in the highest noise symmetric channel *f* = 0.25, where one quarter of all inputs were reversed. While this necessarily required many keypresses to select each target, this is a very effective control under extreme corruption.
  - **Can users select targets in the presence of strong channel bias?** In the biased conditions, users were exposed to a channel with a 45% flip probability on one input, and a 5% flip probability on the other. This level of bias is common in interfaces like motor imagery BCI. Users were able control efficiently under these conditions, with performance around 50% of the theoretical optimum.
  - **Can we achieve a constant factor of the theoretical bounds across all channel conditions?** On average, users were able to select targets with around twice the minimal keypresses possible (Table 4) across all conditions. We would expect better performance with larger *k* and tighter headroom (e.g. *k* = 8, *h* = 0.01).
  - **Does the entropy drop smoothly?** The entropy drops smoothly during selections, and roughly in line with predicted behaviour, as Fig 34 illustrates.

## 8 Conclusions

We have presented a widely-applicable interface for 1-of-*n* selection for marginal reliability inputs with high-reliability displays. This is based on Horstein’s elegant feedback error correction algorithm. This approach can scavenge information from input devices that have previously been considered impractical, and allows arbitrary reliability of control with arbitrarily corrupted inputs—so long as the channel properties are reasonably well known and a low-noise feedback channel is available. In particular, this provides useful control with noisy button-like inputs with reliabilities in the range 65-90%, and heavily biased channels. Partial undo and online adaptation to changing channels are straightforward.

The combination of a nonlinear zooming interface with the Horstein feedback decoder results in an interface that can exploit asymmetric control channels close to the theoretical upper bound. The user interface is simple to implement, adaptable to many selection problems and input device types and our experiments suggest it is easy for users to operate. Our simulator can predict performance early in the design process and provides insight beyond the theoretical asymptotic properties (e.g. impact of burst-mode noise or mis-calibrated channel statistics).

### 8.1 Coding for asymmetric control channels

Good design for asymmetric low-reliability channels should be such that a user does not need to consider how errors should be protected against or recovered from, or how to most efficiently convey their inputs with their limited input budget. Our approach puts errors at the core of the interface design and works from the principle that input will always be noisy and corrupted. This is a different stance than designs which try to “fix-up” inconvenient errors with ad hoc filters and interaction mechanisms. We suggest *explicitly* designing the entropy, channel and line codings that a user must use to communicate, and designing closed-loops at each coding layer that support each of these layers transparently through feedback. Continuous feedback from the system should offer opportunities for control that are adapted to be optimal.

### 8.2 Interface components

The nonlinear zoomed view with alternating diagonal decisions is a simple but effective way of packing options into a 2D space so that they can be selected among efficiently. It reduces all interaction to binary left/right choices, but still allows complete freedom to select any region on the plane. It is transparent to users who only need to focus on their target and decide on which side of a dividing line it lies. Adapting to multi-state noisy button channels (*q*-ary inputs) is straightforward, and each input symbol can have different reliability. Incorporating undo functionality from infrequent reversal channels as found in a hybrid BCI is elegant and conveniently parameterisable in terms of information to be reversed.

### 8.3 Limitations and caveats

Closed-loop interaction allows efficient channel coding like the Horstein decoder to be used without users even being aware of its application, but comes at the cost of making users feedback-bound. This reduces opportunity for learning, since the interface structure is not stable, and has implications on the latency of the feedback channel, both in terms of the display update and the user perceptual delay. In most assistive technology contexts, the input rate is so much slower than feedback that this is not significant, but latency may be a more significant issue when applying this approach domains with frequent updates. Our approach requires a mapping of symbols to a 1D line or 2D plane, but it also requires symbols to be “bundled up” into codewords for efficient coding. This presents interface challenges in terms of labelling and logically organising targets. In some cases, this is straightforward (e.g. navigating a filesystem); in others it may be difficult to organise large numbers of symbols such that they remain identifiable. The tension between efficient bundling of decisions and latency means that some interactions cannot be meaningfully improved by this approach, such as real-time control where decisions among a small set of alternatives must be issued frequently. Similarly, efficient selection with a Horstein decoder requires that users commit to a decision until selection completes. A user changing his or her mind during selection requires more thought, but Section 6.3.2 indicates that the decoder can be configured to be surprisingly robust to a change of target partway through selection.

The Horstein algorithm is not a panacea. A binary input with 65% accuracy is technically usable, but still unbearably slow to operate for most uses. The *theoretical* best rate will require 15 inputs/bit, and a practical *k* = 8 configuration gives ≈26 inputs/uncorrected bit at this error level; this is equivalent to perhaps 120 inputs per correct English word emitted assuming an efficient entropy coder. Decoding is also sensitive to the configuration of the error level. Small changes in measured versus true channel noise can introduce severe penalties if the decoder configuration is optimistic. Although we have demonstrated online adaptive schemes which can cope with of mis-calibration, these are relatively slow to adjust and some inputs may degrade too quickly to retain effective control. The decoder can cope with user mistakes within the configured headroom, assuming errors are approximately iid However, perceptual errors may be more complex than this simple model allows for.

### 8.4 Results

#### 8.4.1 Monte Carlo simulations

Our numerical simulations demonstrate that this decoder is near-optimal across a range of real-world conditions outside of the theoretical predictions. While it is relatively sensitive to calibration with true channel characteristics, it is possible to bound the channel with sufficient headroom to cope with minor fluctuations in reliability at a cost of some loss of input rate. The Horstein decoder can reduce the error to a level that a backspace decoder can “mop up” any remaining error and still retain control very close to the theoretical optimum. Our simulations show the approach works across a full spectrum of biased channels without modification and functions effectively even in the presence of non-stationary noise. In simulation, the algorithm can adapt online to changing signal conditions, including changes in bias. Our results with heavily biased channels are particularly promising as these are frequently encountered in marginal reliability input devices and adoption of this style of interface could render many otherwise frustrating inputs usable. The ability to integrate probabilistic classifiers, and hybrid input devices with infrequent reversal channels (such as EMG-triggered undo) make this an attractive fundamental component to build reliable assistive technology interfaces.

#### 8.4.2 Human-in-the-loop user trials

The user trials indicate that the numerical simulations generalise to the human-in-the-loop case, and that the interaction design based on nonlinear zooming is sufficiently transparent that non-expert users can *immediately* control systems with extreme input corruptions. User performance is very close to that predicted by numerical simulations and suggests that the interface is transparent to users.

### 8.5 Outlook

There are many niches were interaction has been too unreliable to be useful. Some of these marginal reliability input devices are of minor importance, such as setting parameters on camera underwater in a diving suit. Some are of utmost importance to those who depend on them; control for locked-in users with unreliable BCI control. Our contribution is a technique to form these into reliable, efficient inputs with a simple visualisation that is transparent to the user. There remain many interesting design challenges in using the components we have presented to bridge the information theoretic optimal algorithms and the cognitive and ergonomic human constraints on an interface.

## Supporting information

S1 File (PY)

S1 Video (MP4)
